# Supplementary material for: Improving computational tumor ploidy estimation in complex cancer genomes through flow-cytometry-guided calibration
Source: Cell Rep Methods. 2026 Aug 31;6(10):101570. doi: 10.1016/j.crmeth.2026.101570 (PMC13721756; doi:10.1016/j.crmeth.2026.101570)
Supplement: Supplementary file 1 [file mmc1.pdf]

Cell Reports Methods, Volume *6*

Supplemental information

Improving computational tumor ploidy estimation in complex cancer genomes through flow-cytometry-guided calibration

Thomas Butters, Dahmane Oukrif, Punn Tannirandorn, Ianthe AEM van Belzen, Jianan Chen, Christopher Davies, Runtian Lin, Solange De Noon, Dalil Taher, Chloe Cutler, Mark Markov, Anna Tollit, Fatine Oumlil, Christopher D. Steele, Matthew Fittall, Yanping Guo, Isidro Cortés-Ciriano, Peter Van Loo, Adrienne M. Flanagan, Nischalan Pillay, and Maxime Tarabichi

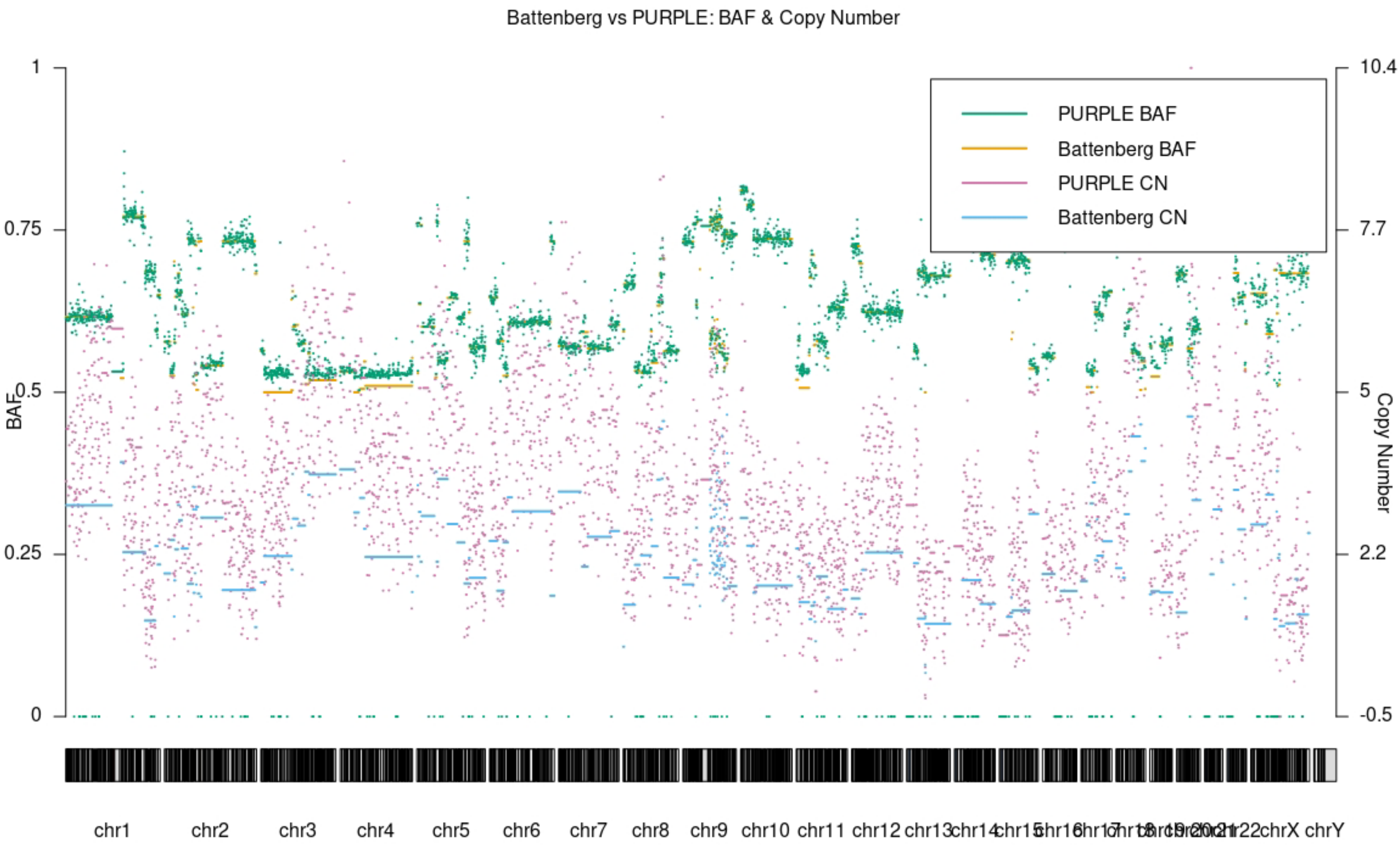

Supplementary Figure 1. Comparison of mirrored BAF and copy number between Battenberg and PURPLE on USARC-35, related to Figure 1.

The BAF are similar between the two CNA callers, however, due to the hyper-segmentation of the genome by PURPLE, the copy number is less concordant.

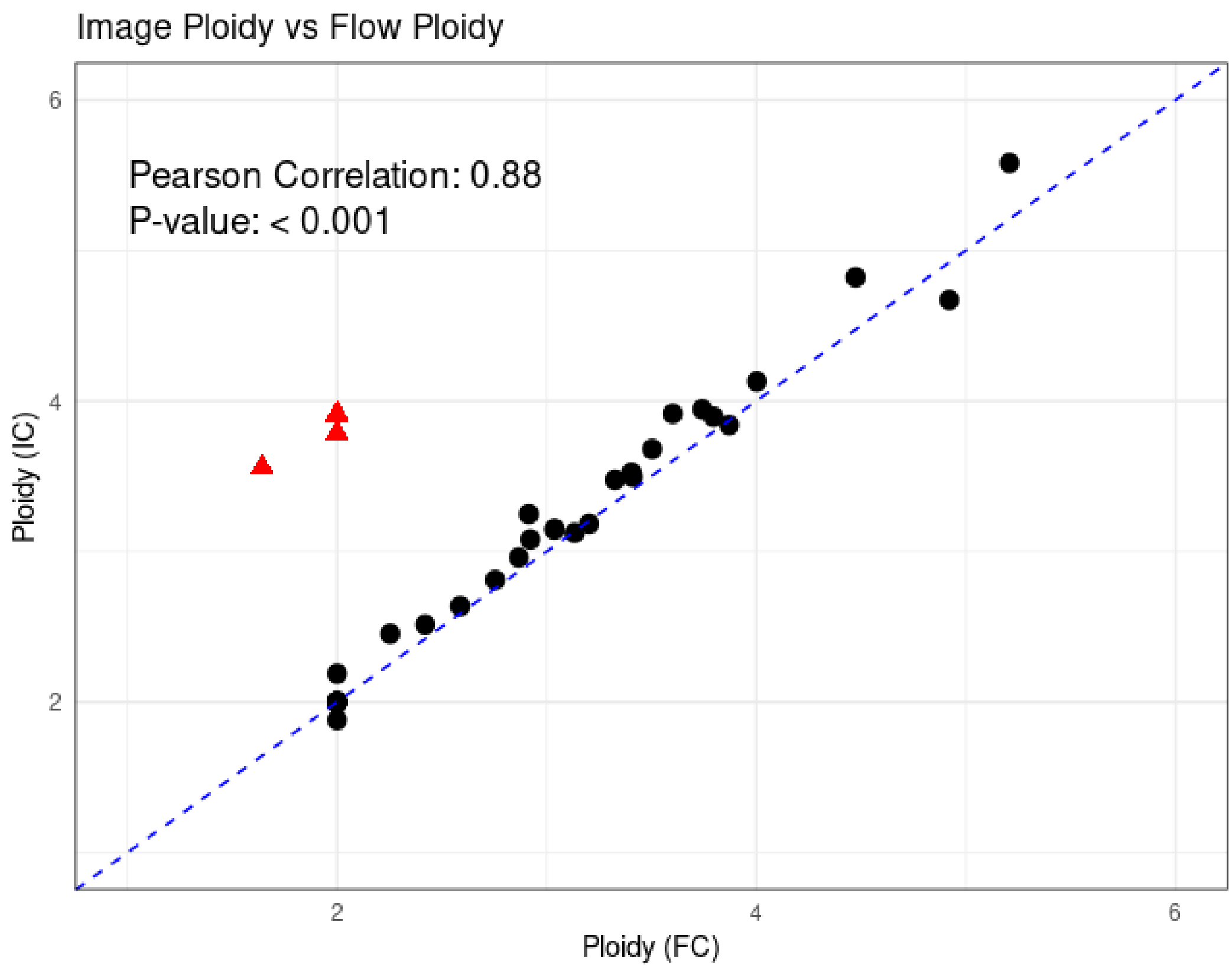

Supplementary Figure 2. Correlation between ploidy estimates derived by image cytometry (IC) and flow cytometry (FC), related to Figure 2.
Linear regression showing the correlation of ploidy estimates derived by IC vs FC of the primary tumour populations of USARC and OS samples. There is a very strong

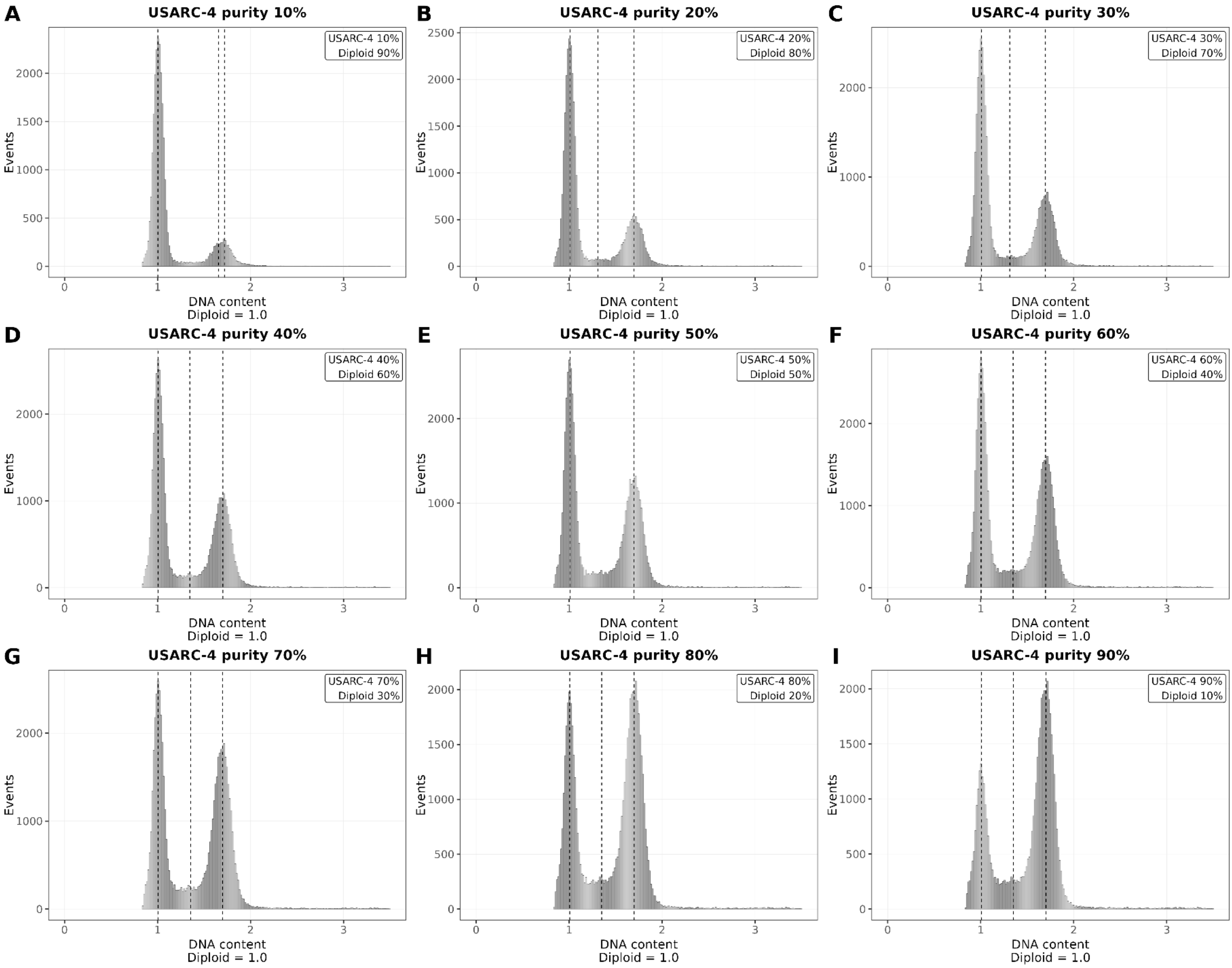

Supplementary Figure 3. Simulated USARC-4 tumour purity sweep, related to Figure 2.
Histograms show normalised DNA-content distributions generated by mixing USARC-4 tumour events with diploid control events at increasing tumour fractions from 10% to 90% (A–I). DNA content was normalised such that the diploid peak is centred at 1.0. Dashed vertical lines indicate density-detected peaks, and inset labels show the tumour and diploid proportions used for each simulated mixture.

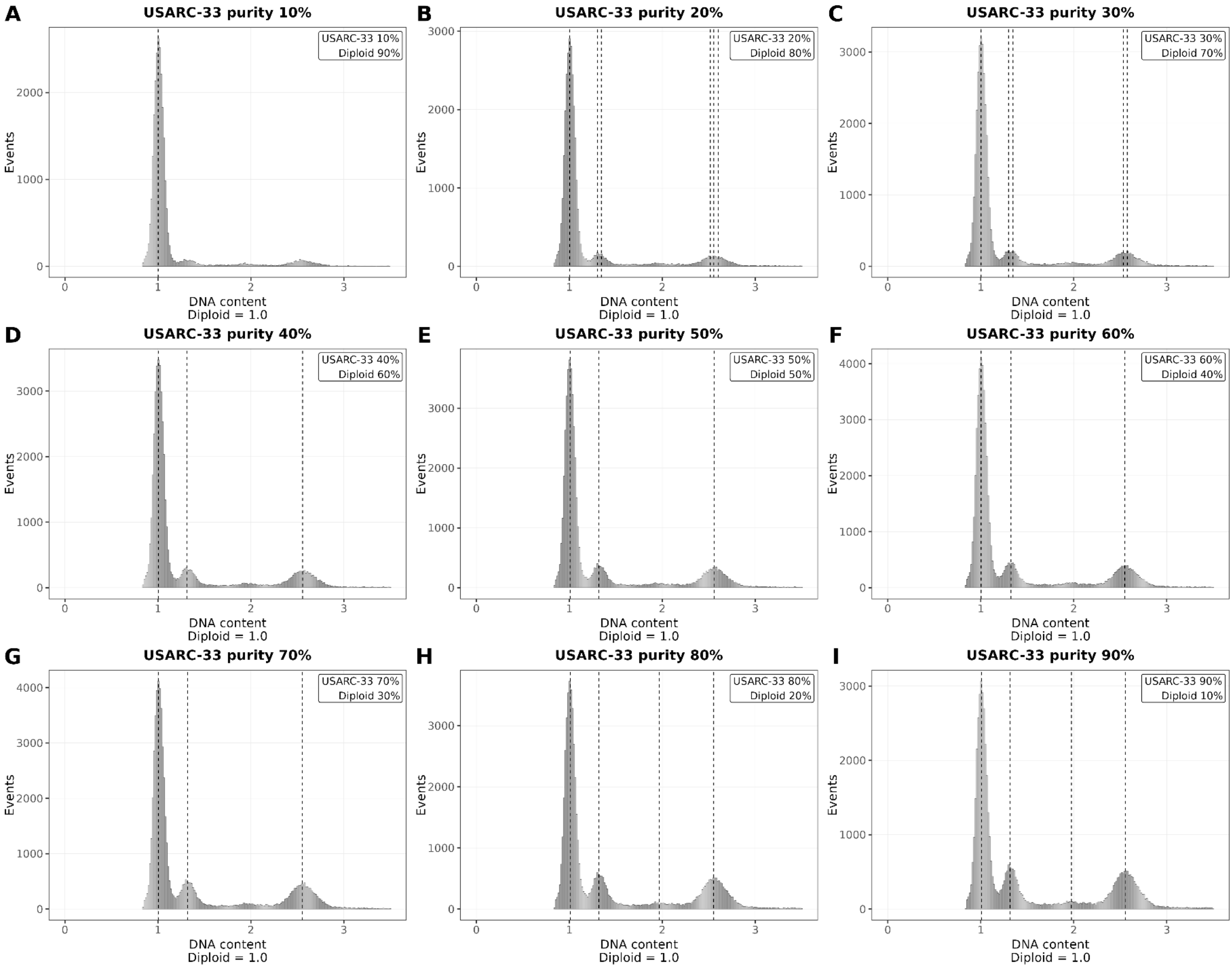

Supplementary Figure 4. Simulated USARC-33 tumour purity sweep, related to Figure 2. Histograms show normalised DNA-content distributions generated by mixing USARC-33 tumour events with diploid control events at increasing tumour fractions from 10% to 90% (A–I). DNA content was normalized such that the diploid peak is centred at 1.0. Dashed vertical lines indicate density-detected peaks, and inset labels show the tumour and diploid proportions used for each simulated mixture.

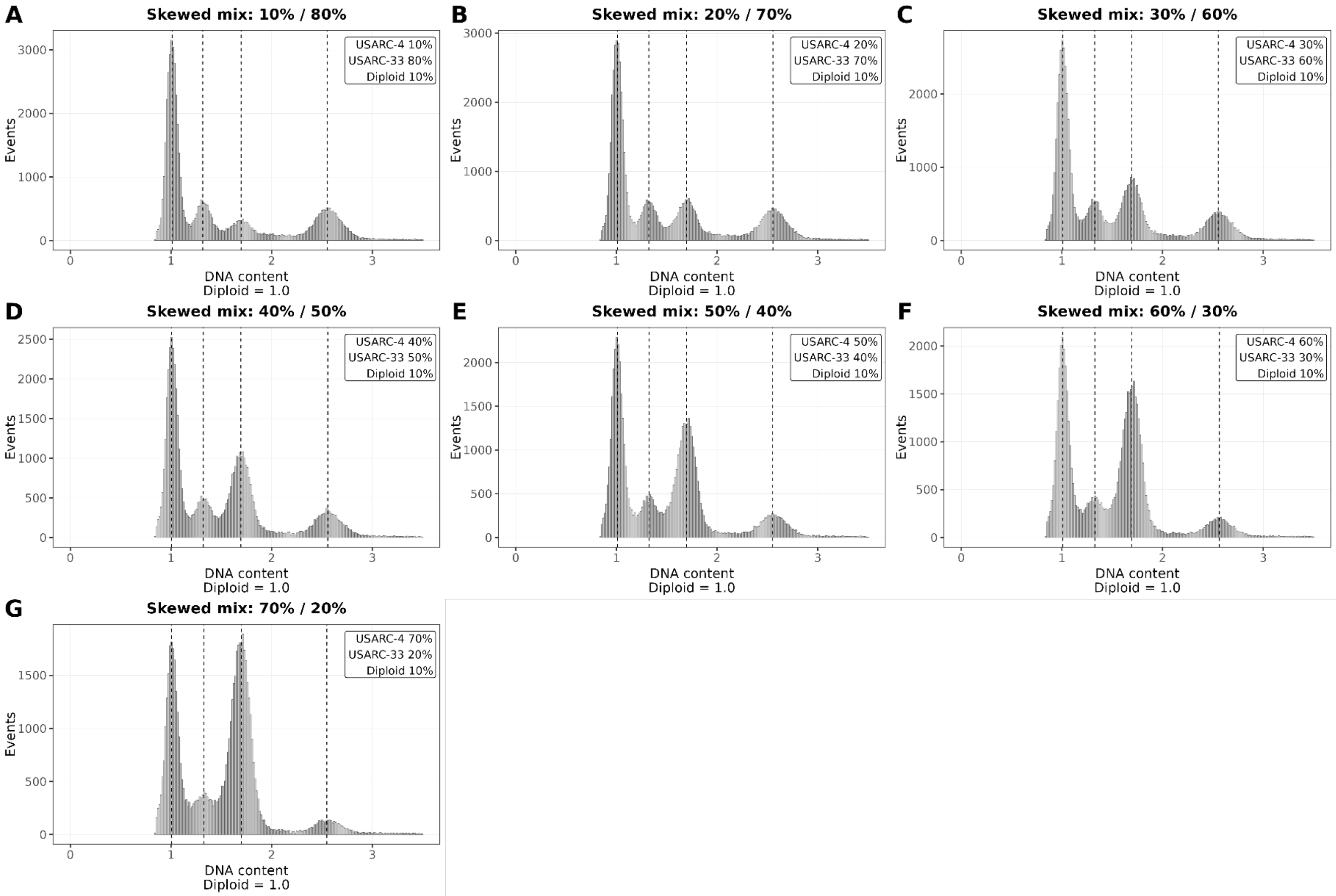

Supplementary Figure 5. Skewed simulated USARC-4 and USARC-33 tumour mixtures, related to Figure 2. Histograms show normalized DNA-content distributions generated by mixing USARC-4 and USARC-33 tumour events at reciprocal proportions while maintaining a fixed 10% diploid component (A–G). USARC-4 increases from 10% to 70%, while USARC-33 decreases from 80% to 20%. DNA content was normalized such that the diploid peak is centered at 1.0. Dashed vertical lines indicate density-detected peaks, and inset labels show the mixture proportions used for each simulated sample.

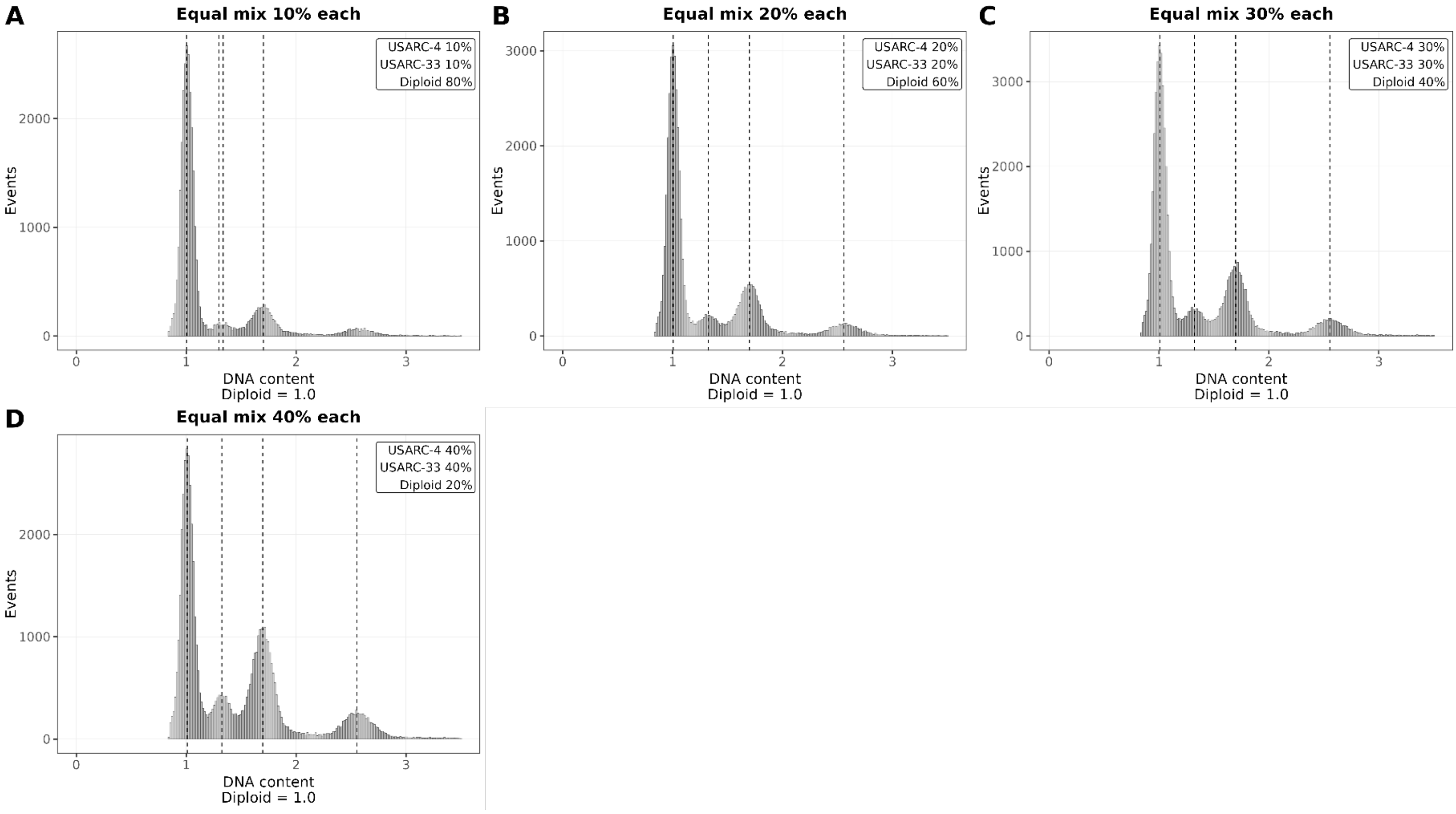

Supplementary Figure 6. Equal simulated USARC-4 and USARC-33 tumour mixtures, related to Figure 2. Histograms show normalized DNA-content distributions generated by mixing USARC-4 and USARC-33 tumour events at equal proportions, ranging from 10% each to 40% each (A–D), with the remaining events comprising the diploid component. DNA content was normalized such that the diploid peak is centered at 1.0. Dashed vertical lines indicate density-detected peaks, and inset labels show the USARC-4, USARC-33, and diploid proportions used for each simulated mixture.

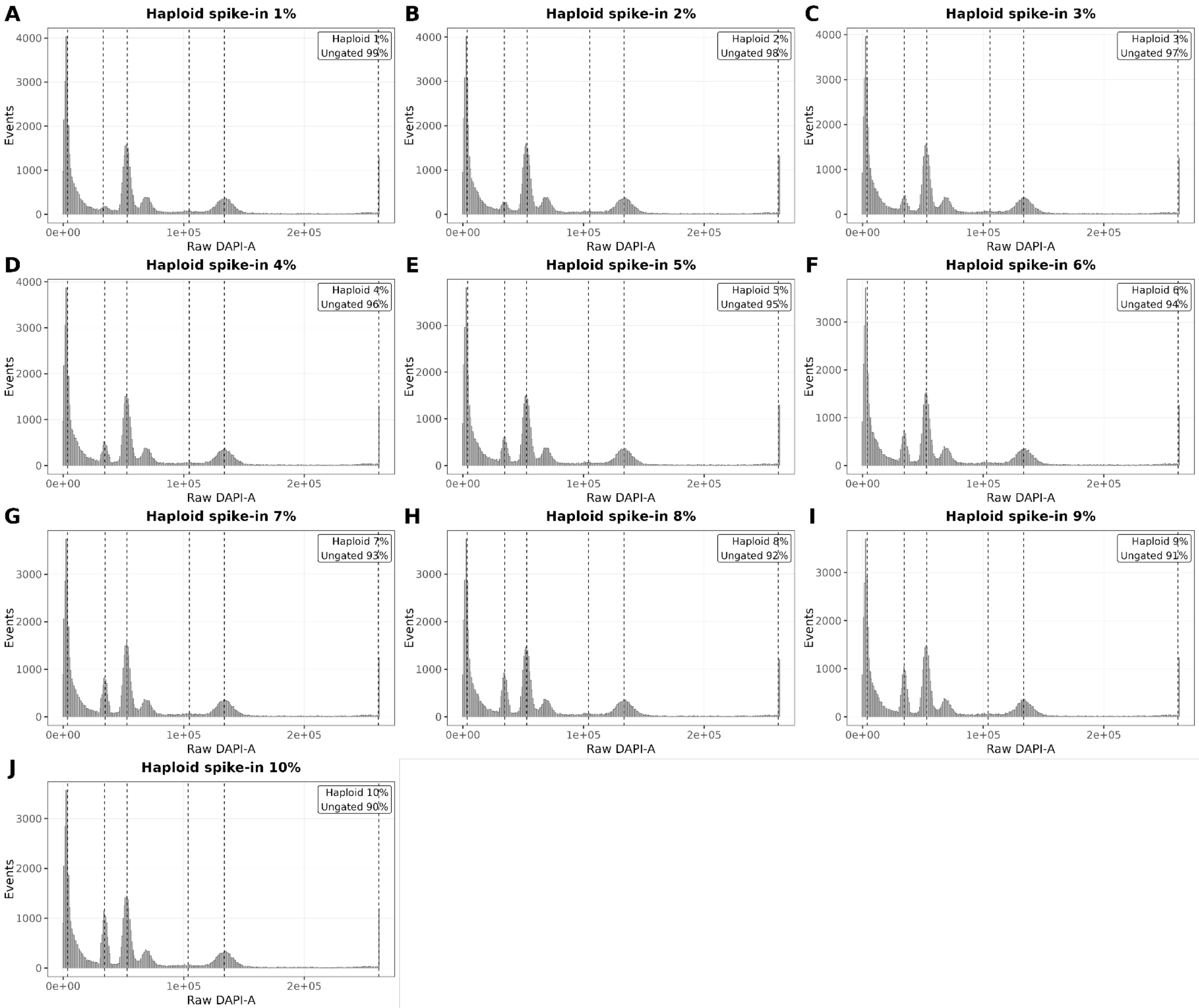

Supplementary Figure 7. Synthetic haploid spike-in sweep in ungated 3269 events, related to Figure 2. Histograms show raw DAPI-A distributions generated by adding a synthetic haploid-like population, derived by halving the first gated 3269 tumour peak, into the unmodified ungated 3269 background at spike-in fractions from 1% to 10% (A–J). Dashed vertical lines indicate density-detected peaks, and inset labels show the synthetic haploid and ungated proportions used for each mixture.

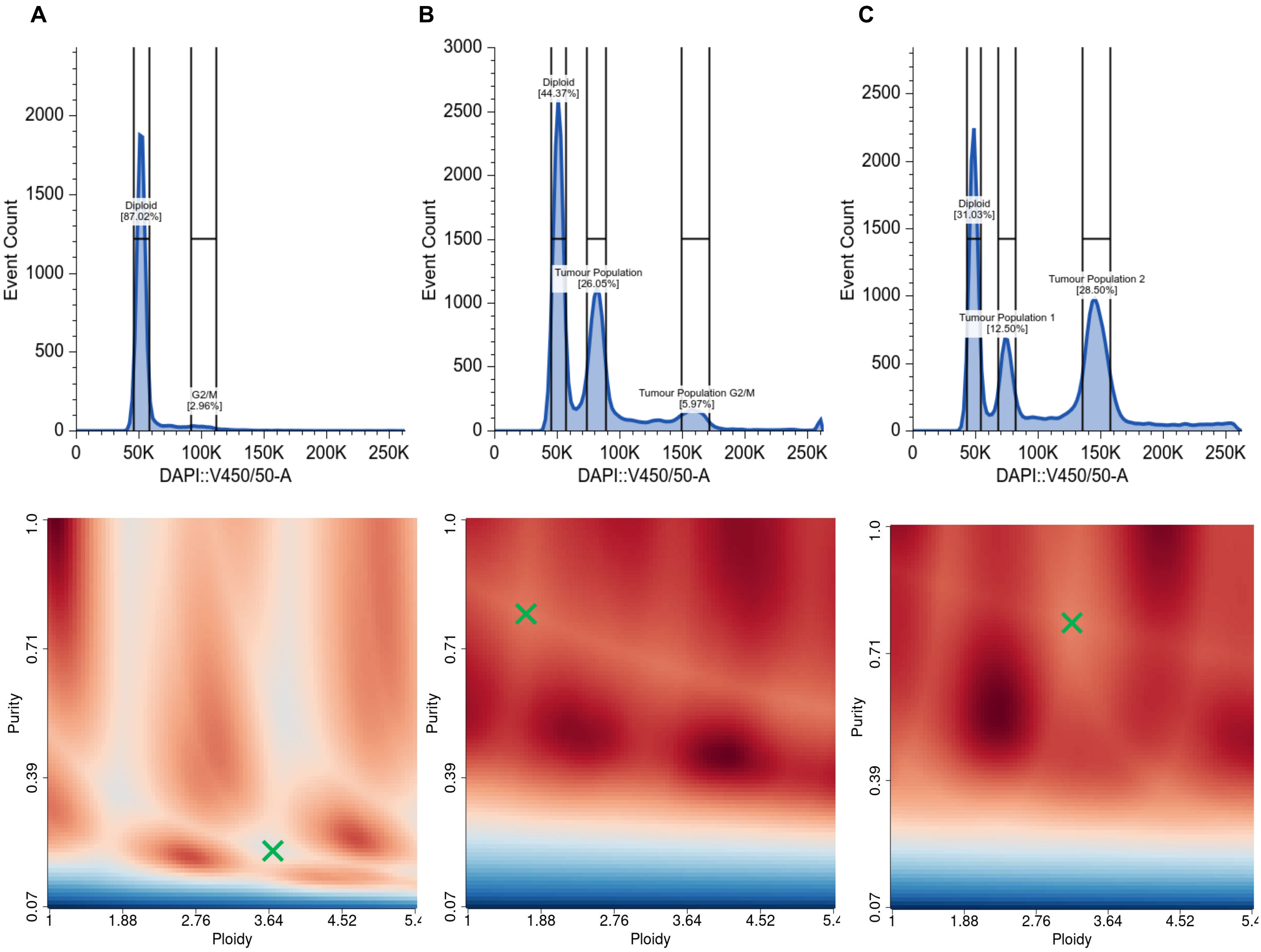

Supplementary Figure 8. Examples of discordant ploidy solutions between flow cytometry and Battenberg in USARC samples, related to Figure 2.

A) An example of a USARC sample which is near-diploid by FC (top), but has been erroneously given a ploidy of 3.68N by Battenberg on the sunrise plot (bottom) as indicated by a green cross. There is an equivalent solution at a ploidy of approximately 1.9N at a higher purity estimate. B) An example of a USARC sample which has a ploidy of 3.2N by FC (top), but has been erroneously given a ploidy of 1.92N by Battenberg on the sunrise plot (bottom). There is an equivalent solution at a ploidy of approximately 3.5N at a lower purity estimate. C) An example of a USARC sample which has multiple aneuploid tumour populations with ploidies of 3.1 and 6.0 respectively. Battenberg has chosen a ploidy solution equivalent the first aneuploid population, but not the largest tumour population. Ploidy solutions exist for this tumour at a ploidy of approximately 6.6 (not-shown).

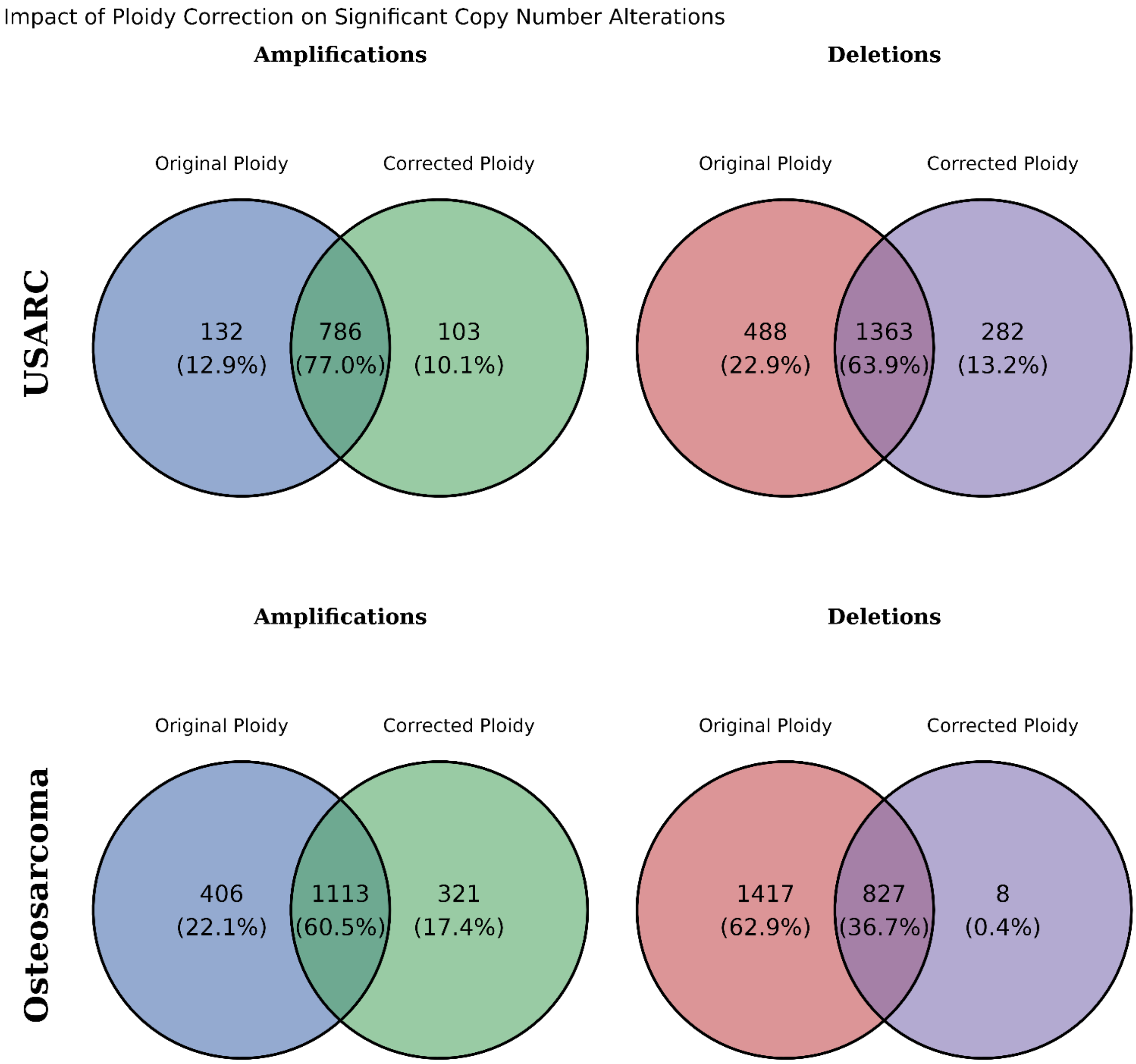

Supplementary Figure 9. Impact of ploidy correction on significant copy number alteration calls in USARC and osteosarcoma cohorts, related to Figures 3 &4.
Venn diagrams compare significant copy number alterations identified using original ploidy estimates versus ploidy-corrected estimates. Results are shown separately for amplifications and deletions in the USARC cohort (A) and the osteosarcoma cohort (B). Numbers indicate the count of significant alterations unique to the original ploidy analysis, shared between both approaches, or unique to the corrected ploidy analysis. Ploidy correction retained the majority of amplification calls in both cohorts, while a larger shift was observed for deletion calls, particularly in osteosarcoma, where most deletions were unique to the original ploidy analysis.

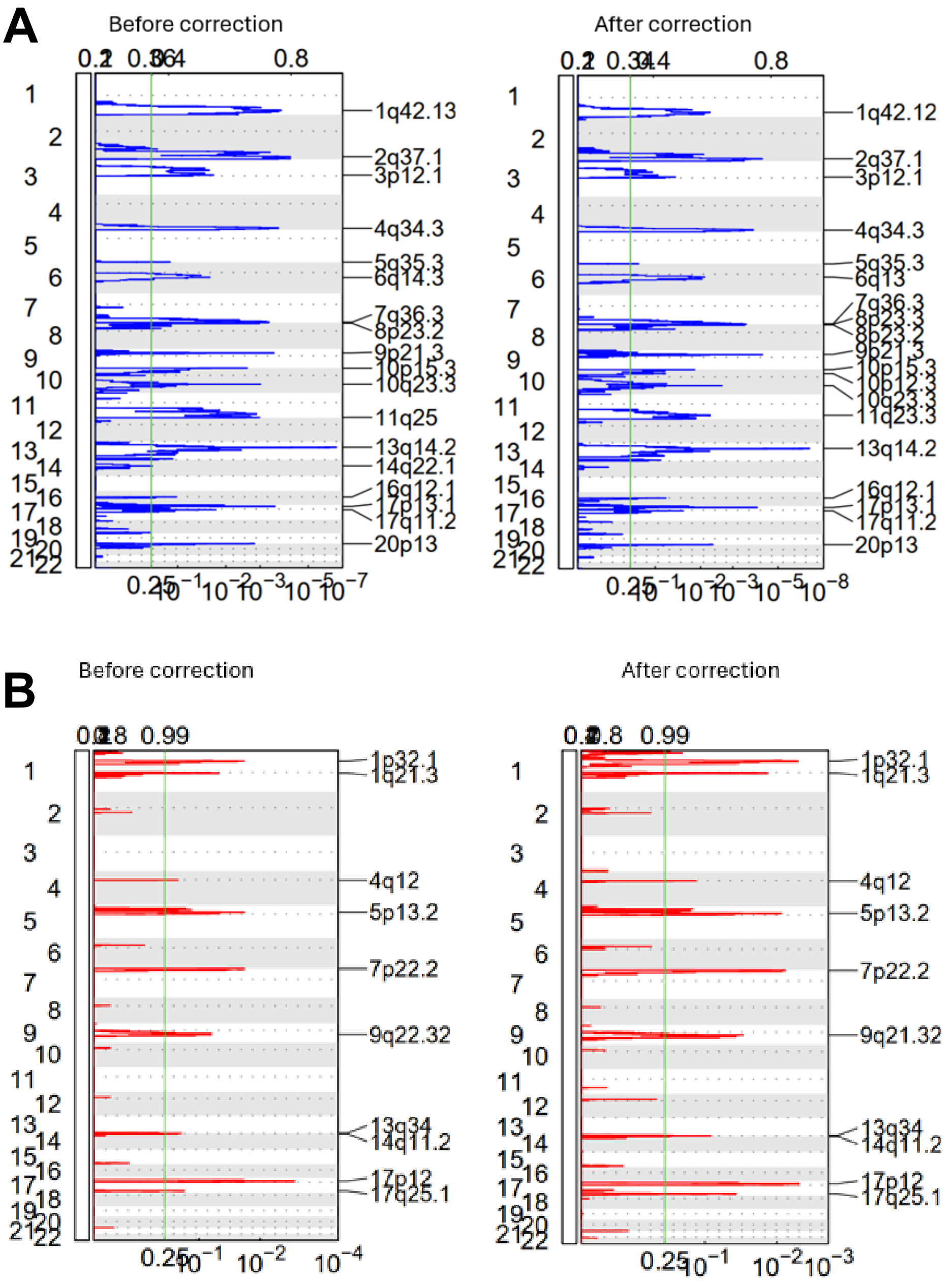

Supplementary Figure 10. Effect of ploidy correction on GISTIC copy-number calls, related to Figures 3 &4..
Genome-wide GISTIC deletion (A) and amplification (B) profiles are shown before and after ploidy correction. The x-axis shows GISTIC significance, reported as −log10(q-value), and the y-axis shows chromosomal position across autosomes 1–22. Vertical green lines indicate the significance threshold, and selected significant peaks are labelled by cytoband. Ploidy correction altered the significance of some recurrent events, with additional arm-level or focal peaks crossing the GISTIC threshold after correction.

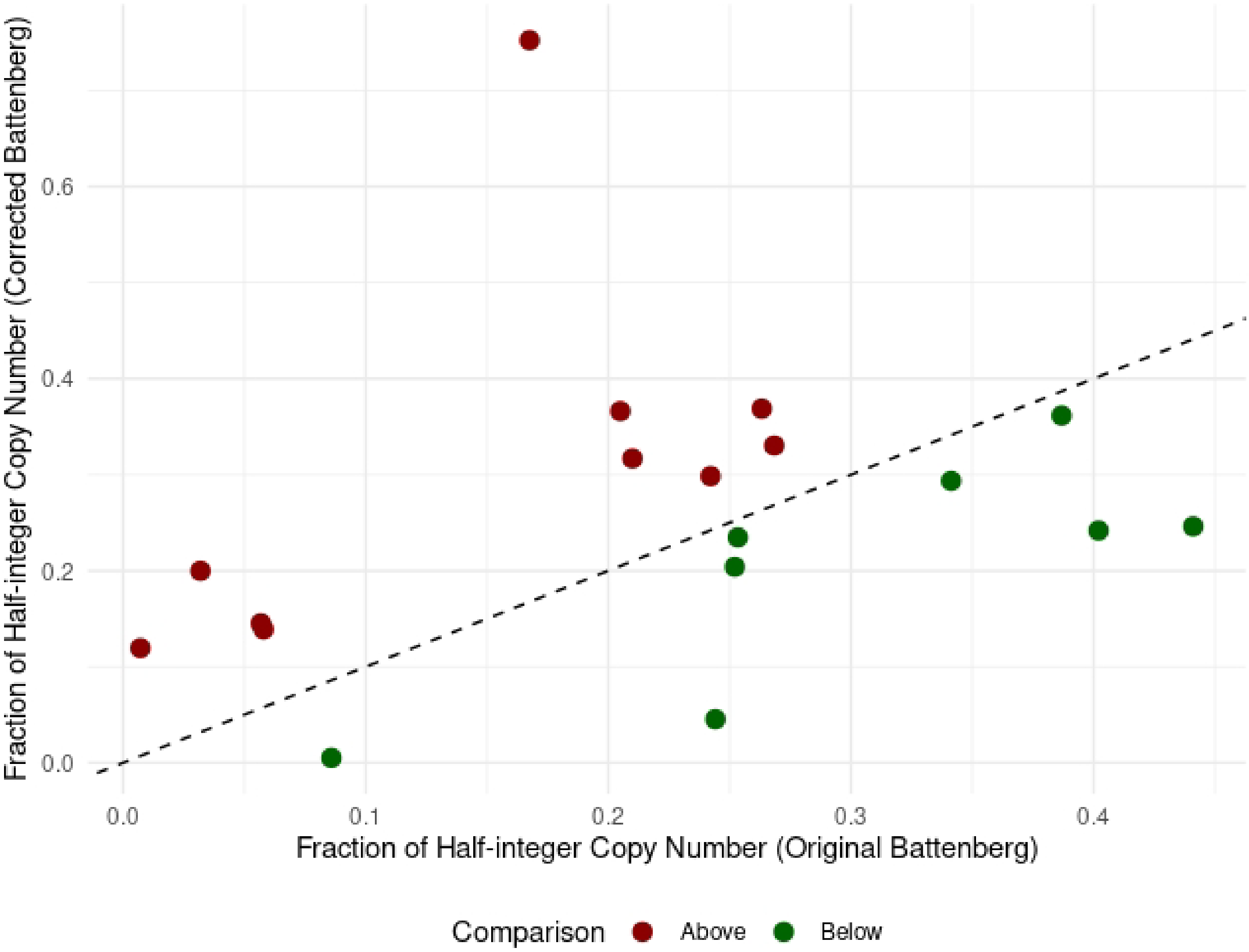

Supplementary Figure 11. Impact of ploidy correction on the proportion of half-integer copy number states, related to Figure 4.

Scatter plot comparing the proportion of each tumour genome lying at half-integer values using Battenberg copy number modelling before and after ploidy correction. Green points lying below the y = x line represent samples where the original Battenberg profiles harbour a greater proportion of half-integer values. Red points lying above the y = x line represent samples where the corrected Battenberg profiles harbour a greater proportion of half-integer values. The plot demonstrates that using the presence of half-integer values to correct ploidy in-silico, is uninformative for the USARC dataset.

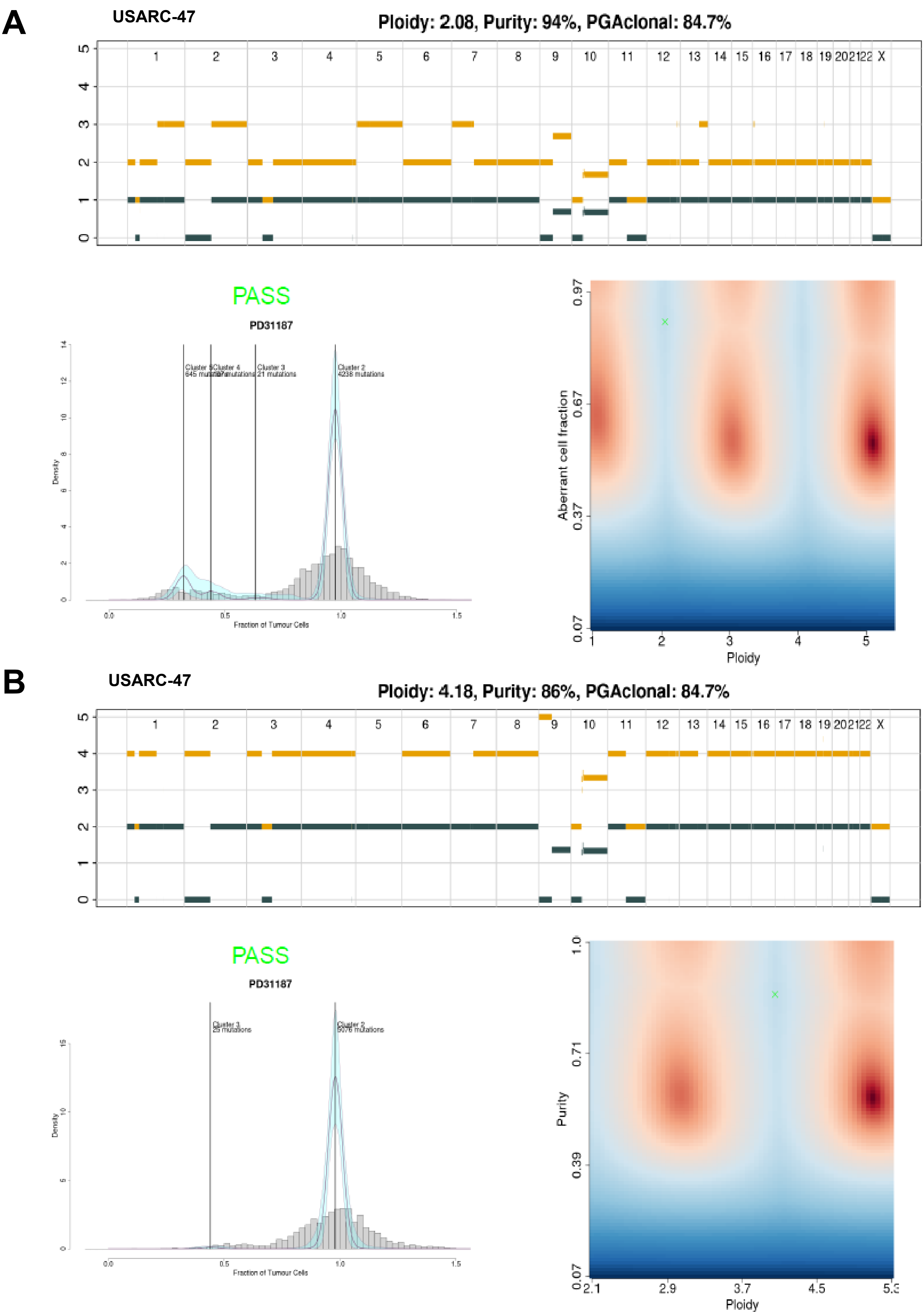

Supplementary Figure 12. SNV clustering analysis of USARC sample USARC-47 before and after flow cytometry ploidy correction, related to Figure 4..

SNV clustering analysis of USARC sample USARC-47 before (A) and after (B) ploidy correction by FC. Each figure consists of copy number profile (top), DPClust SNV clustering analysis (bottom left), and sunrise plot (bottom right). The CCF of the clonal SNV peak does not change before and after correction, and this is most likely due to the presence of multiple possible purity values for each ploidy solution (evident on sunrise plot).

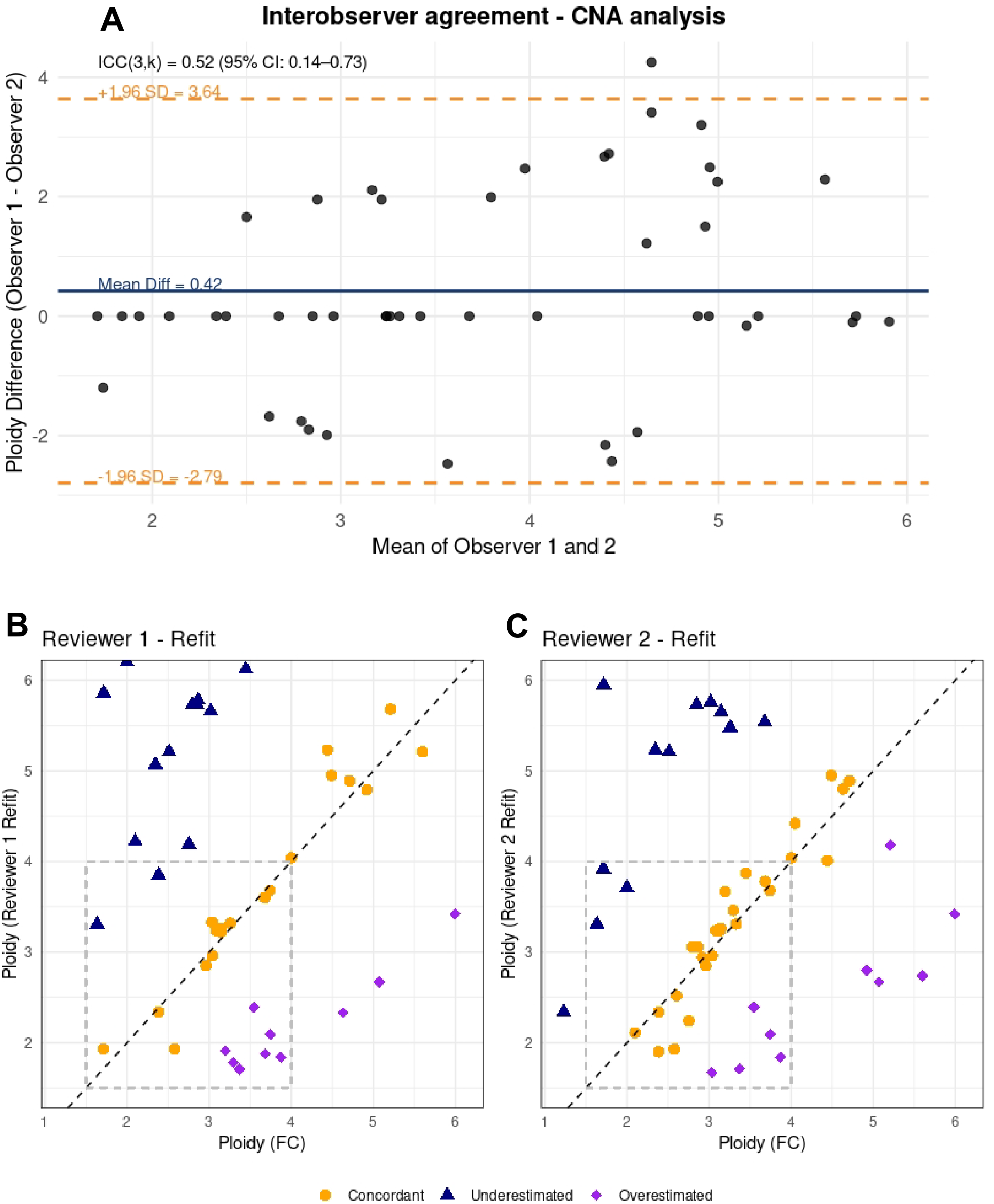

Supplementary Figure 13. Interobserver agreement and correlation with flow cytometry for refitted Battenberg ploidy estimates, related to Figure 4..

(A) Bland-Altman plot showing low-moderate interobserver agreement between refitted Battenberg ploidy estimates generated by reviewer 1 and reviewer 2. (B) Correlation between FC and refitted Battenberg ploidy estimates generated by expert 1 (B) and expert 2 (C). There is a discrepancy of 51% and 38% respectively between reviewers 1 and 2 and the FC ploidy.

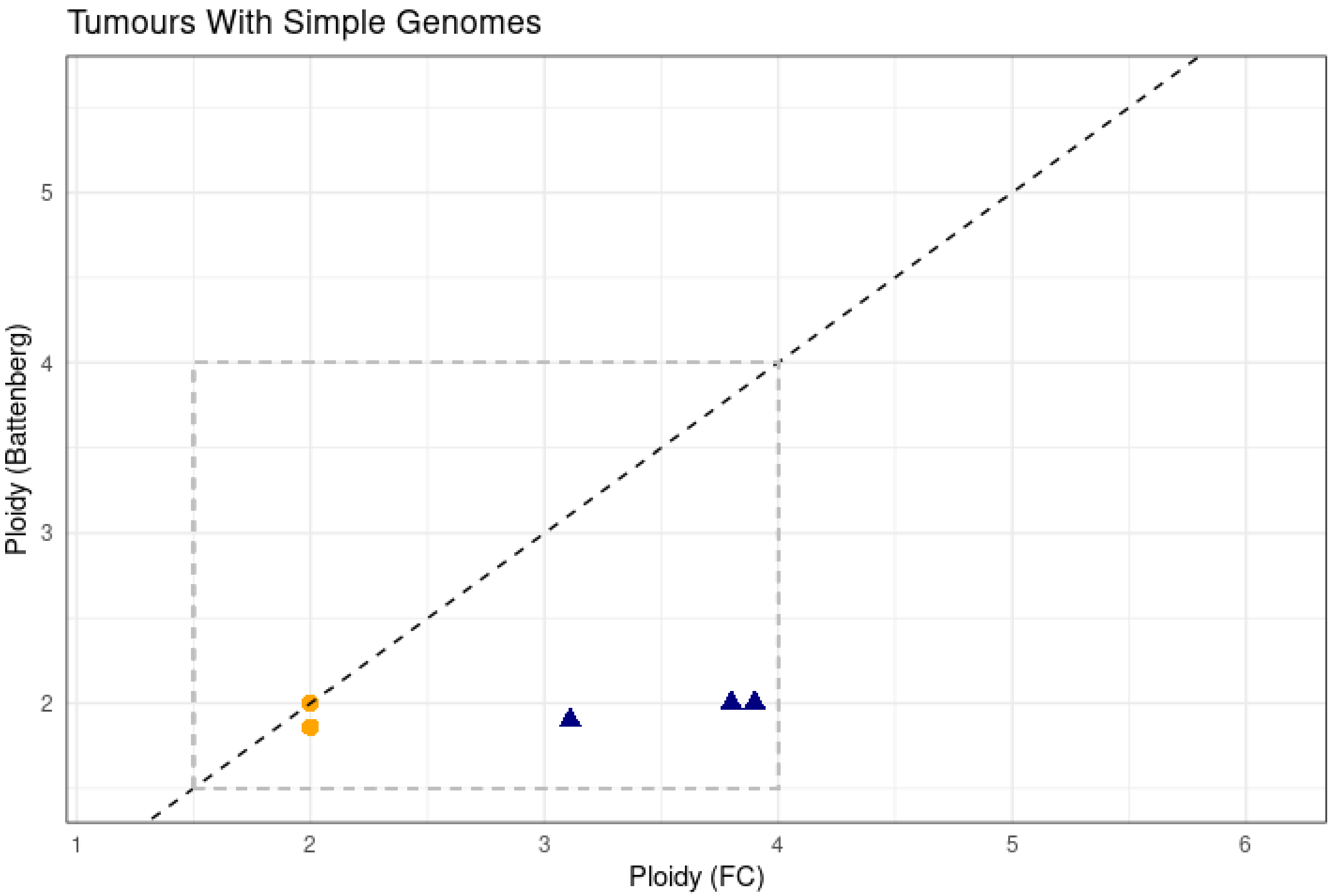

Supplementary Figure 14. Correlation between flow cytometry and Battenberg ploidy estimates in tumours with simple genomes, related to Figure 4..

The figures shows the correlation between FC-derived ploidy estimates of the primary tumour peaks, and Battenberg-derived estimates of tumour ploidy in tumours with relatively simple genomes including SS, LGFMS, SFT, and LG-OS. There is a 11% discrepancy. 3/27 samples (navy) were underestimated compared with the FC ploidy.

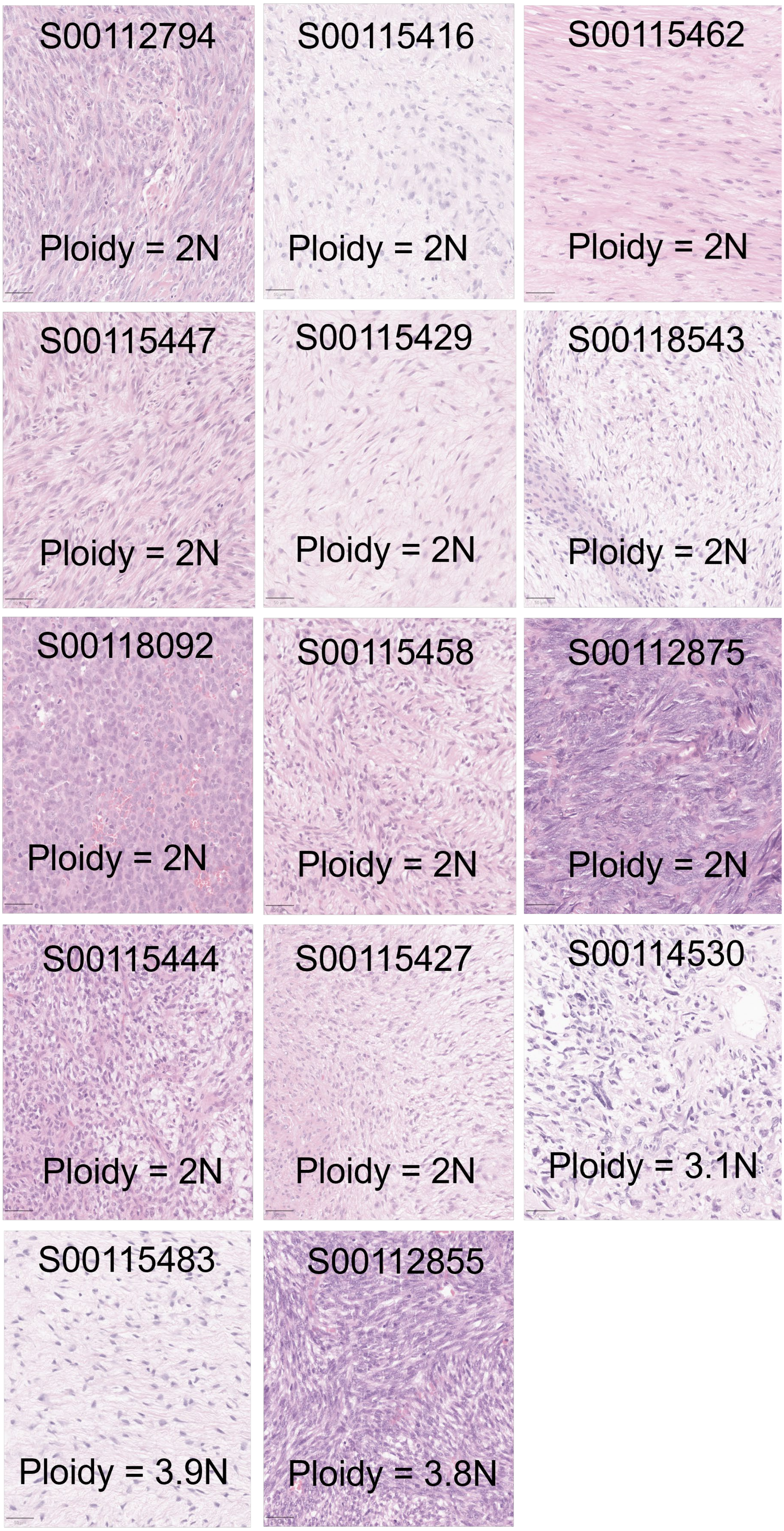

Supplementary Figure 15. Example tiles from H&E scanned whole-slide images of chromosomally simple tumours, related to Figure 4..

Example tiles from H&E-scanned WSI of chromosomally simple tumours at x40 magnification, with relevant FC-derived ploidy measurements. Scale bars in the bottom left of each tile measure 50 µm.

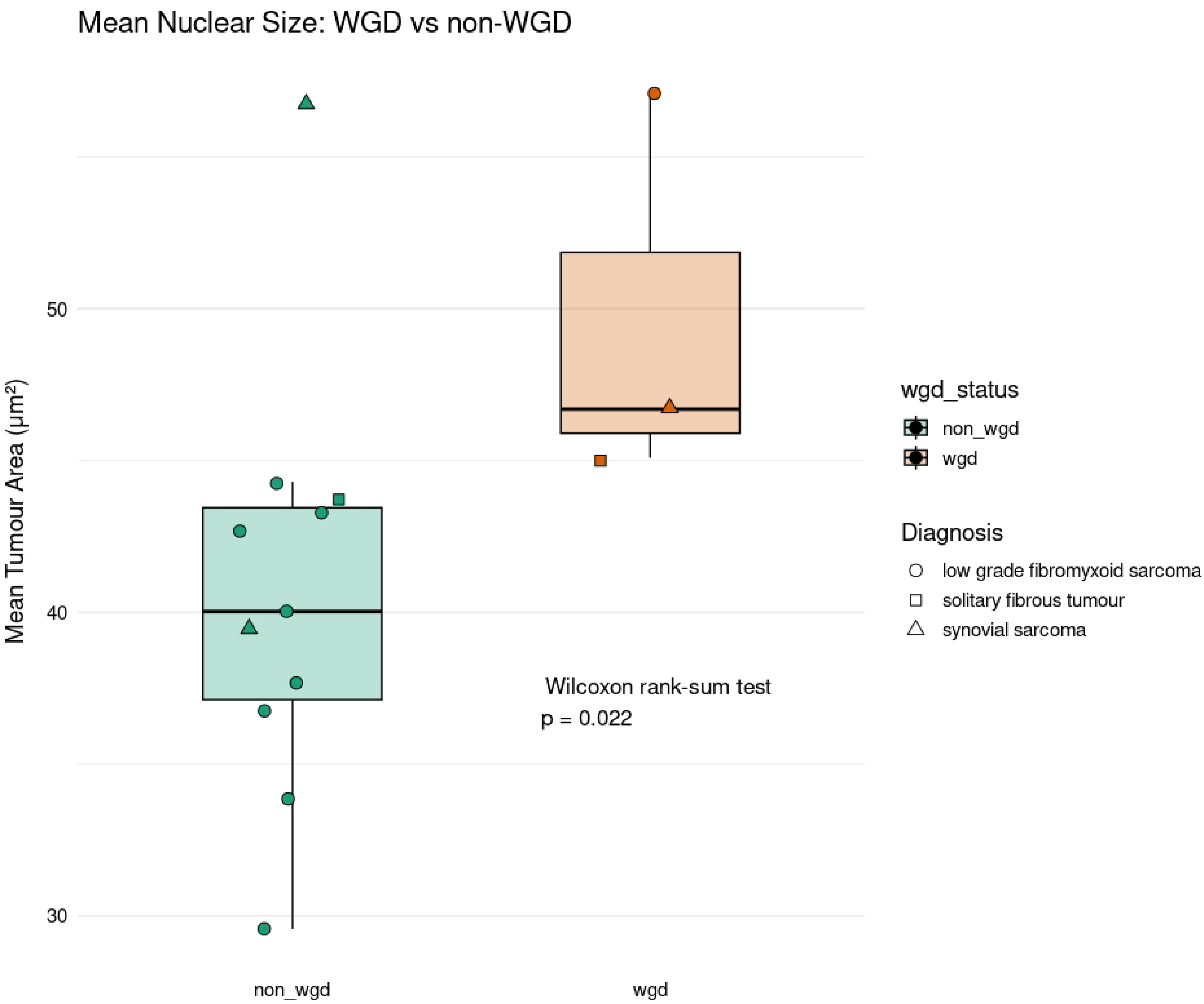

Supplementary Figure 16. Mean nuclear size of translocation-associated sarcomas grouped by whole-genome doubling status, related to Figure 4..

The figures shows the mean nuclear size of a subset of 14 translocation-associated sarcomas with relatively simple genomes. By CellVit analysis, the WGD tumours (identified through FC) have a greater mean nuclear size than all but one diploid sarcoma.

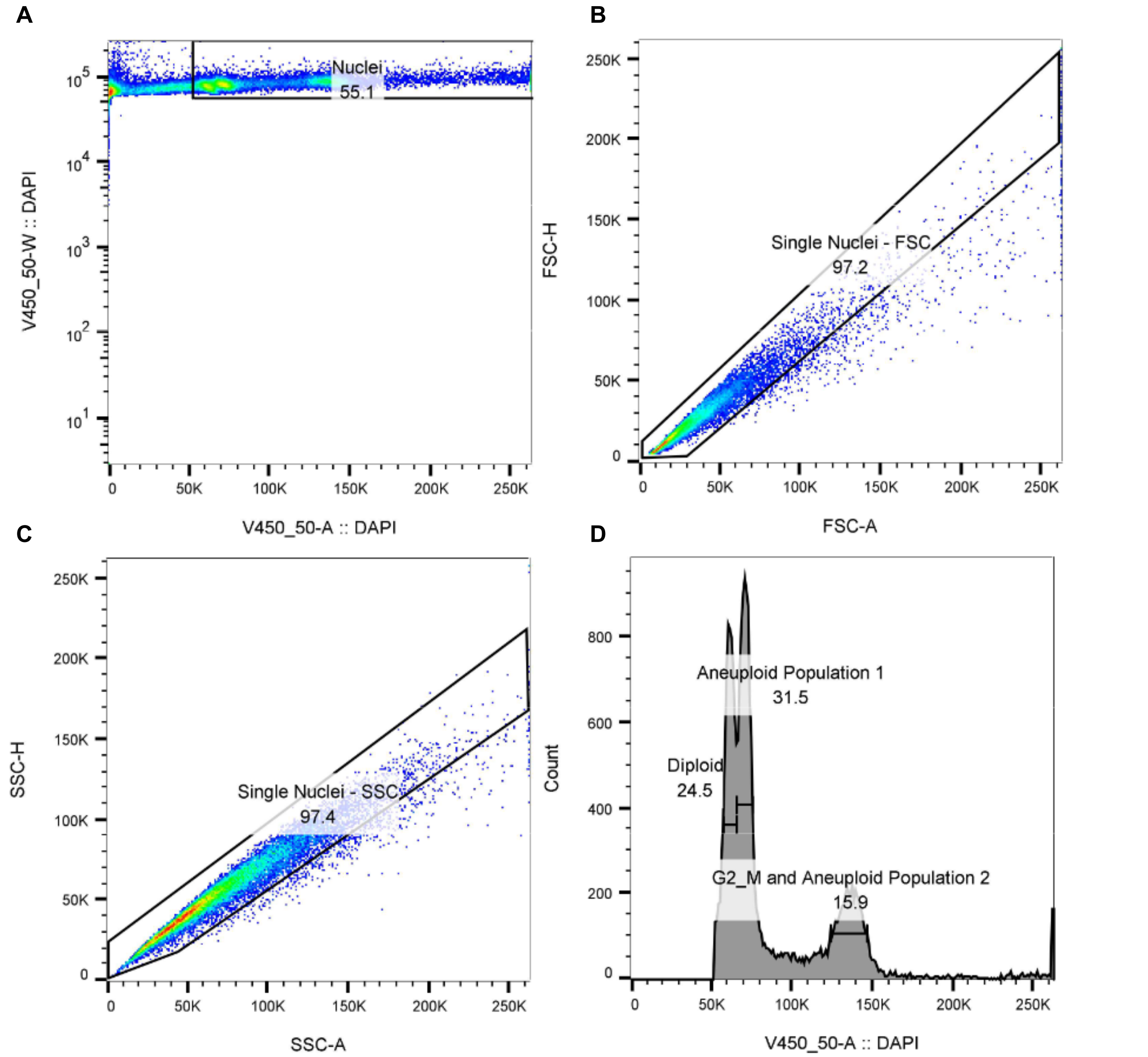

Supplementary Figure 17. Flow cytometry gating strategy for isolating nuclei and excluding debris and doublets, related to STAR Methods.

A) Using DAPI.width plotted against DAPI.area, the debris is excluded from the nuclei by identifying the first 'hotspot' which does not touch the y-axis. B+C) Doublets are excluded by creating hierarchical gates with FSC and SSC using the previously gated 'nuclei' population. Nuclei which fall off a linear relationship between area and height are considered as doublets. D) Ploidy histogram showing an aneuploid tumour sample. The first peak is the internal diploid control population. The second peak is the first aneuploid population. The third peak is the G2/M phase of the first aneuploid population. As the third peak has a population of greater than half of the aneuploid population, and a relatively small S-phase, it is possibly a mixture of G2/M nuclei, and a second aneuploid population.

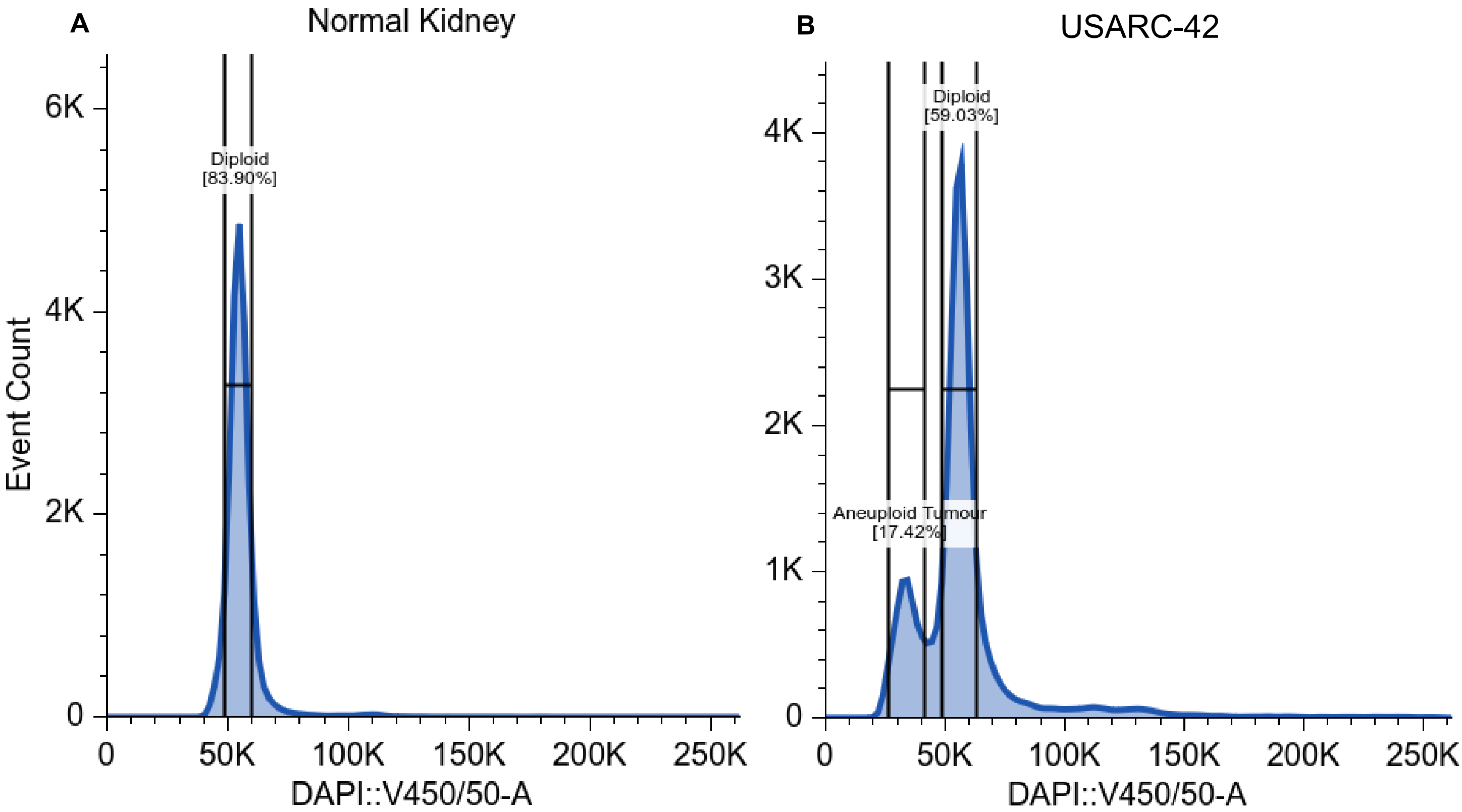

Supplementary Figure 18. Identification of a pseudo-haploidised tumour population in USARC-42 using an external diploid control, related to STAR methods.
Ploidy histogram of USARC-42 which demonstrates a pseudo-haploidised population of tumour nuclei at a ploidy of 1.2N. This haploid tumour exemplifies the importance of using an adequate and standardised external control. At first glance, the aneuploid population may be perceived as the diploid population in the tumour sample as it lies leftmost on the histogram. However, the second peak in the tumour sample lies at a MFI of 57,083AU which is very similar to the external diploid control sample (MFI 55,468AU) compared with the pseudo-haploidised peak (MFI 34,707AU).
