# Supplementary material for: Improving computational tumor ploidy estimation in complex cancer genomes through flow-cytometry-guided calibration
Source: Cell Rep Methods. 2026 Aug 31;6(10):101570. doi: 10.1016/j.crmeth.2026.101570 (PMC13721756; doi:10.1016/j.crmeth.2026.101570)
Supplement: Supplementary file 2 [file mmc3.pdf]

Cell Reports Methods

Article

Improving computational tumor ploidy estimation in complex cancer genomes through flow-cytometry-guided calibration

Graphical abstract

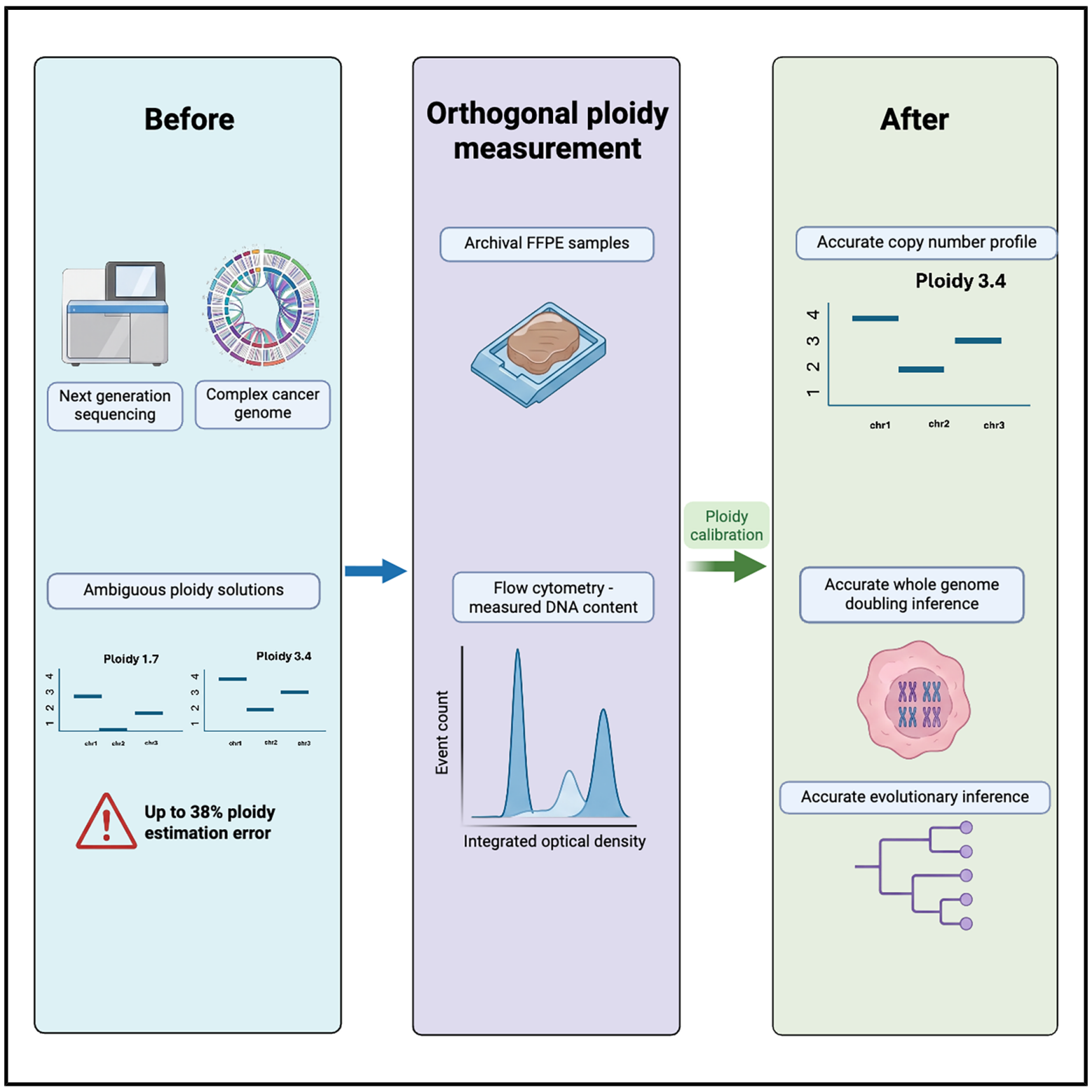

Highlights

- Computational ploidy estimates are inaccurate in up to 38% of complex tumors
- Flow cytometry using FFPE tissue accurately calibrates WGS-based ploidy estimates
- Ploidy correction changes whole-genome doubling calls and copy-number results
- Genome doubling can be missed by sequencing alone in sarcomas with "quiet" genomes

Authors

Thomas Butters, Dahmane Oukrif, Punn Tannirandorn, ..., Adrienne M. Flanagan, Nischalan Pillay, Maxime Tarabichi

Correspondence

a.flanagan@ucl.ac.uk (A.M.F.),
n.pillay@ucl.ac.uk (N.P.),
maxime.tarabichi@ulb.be (M.T.)

In brief

Butters et al. show that sequencing-based estimates of tumor DNA content can be incorrect in genomically complex cancers, distorting interpretation of genome doubling and cancer-gene copy number. Flow cytometry of archival FFPE tissues provides a practical experimental reference, improving genomic analysis and revealing hidden genome doubling in genomically "quiet" sarcomas.

Butters et al., 2026, Cell Reports Methods *6*, 101570
October 19, 2026 © 2026 The Author(s). Published by Elsevier Inc.
https://doi.org/10.1016/j.crmeth.2026.101570

CellPress

Cell Reports Methods

CellPress
OPEN ACCESS

Article

Improving computational tumor ploidy estimation in complex cancer genomes through flow-cytometry-guided calibration

Thomas Butters,[1] Dahmane Oukrif,[1] Punn Tannirandorn,[1,2] Ianthe AEM van Belzen,[5] Jianan Chen,[1] Christopher Davies,[1] Runtian Lin,[1] Solange De Noon,[1,7] Dalil Taher,[8] Chloe Cutler,[2] Mark Markov,[2] Anna Tollit,[7] Fatine Oumlil,[7] Christopher D. Steele,[11] Matthew Fittall,[3] Yanping Guo,[4] Isidro Cortés-Ciriano,[5,6] Peter Van Loo,[9,10] Adrienne M. Flanagan,[1,7,12,*] Nischalan Pillay,[1,7,12,13,*] and Maxime Tarabichi[2,8,12,*]

[1]Research Department of Pathology, Cancer Institute, University College London, London, UK
[2]University College London Medical School, London, UK
[3]Research Department of Oncology, University College London, London, UK
[4]Flow Cytometry TTP, Cancer Institute, University College London, London, UK
[5]European Molecular Biology Laboratory, European Bioinformatics Institute, Hinxton, Cambridge, UK
[6]Wellcome Sanger Institute, Cambridge, UK
[7]Department of Pathology, Royal National Orthopaedic Hospital, Stanmore, UK
[8]IRIBHM Jacques E. Dumont, Université Libre de Bruxelles, Brussels, Belgium
[9]Department of Genetics, The University of Texas MD Anderson Cancer Center, Houston, TX, USA
[10]Department of Genomic Medicine, The University of Texas MD Anderson Cancer Center, Houston, TX, USA
[11]University of California, San Diego, San Diego, CA, USA
[12]These authors contributed equally
[13]Lead contact
*Correspondence: a.flanagan@ucl.ac.uk (A.M.F.), n.pillay@ucl.ac.uk (N.P.), maxime.tarabichi@ulb.be (M.T.)
https://doi.org/10.1016/j.crmeth.2026.101570

MOTIVATION Despite the established role of tumor ploidy in cancer genomics, widely used computational methods for ploidy estimation exhibit high error rates. Although the measurement of cellular DNA content by FC has long been established as a gold standard, the integration of FC performed on formalin-fixed paraffin-embedded (FFPE) tissue into modern genomic workflows remains poorly documented. This paper bridges this gap, providing a clear framework for the use of FC as an orthogonal and complementary technique in genomic analysis.

SUMMARY

DNA ploidy is an important predictor of tumor behavior and prognosis, and its accurate estimation is essential for robust genomic analysis in translational cancer research and diagnostics. However, the most common *in silico* methods for ploidy estimation using next-generation-sequencing-based copy-number aberration (CNA)-calling algorithms are often inaccurate due to inherent ambiguity in fitting ploidy solutions. This study evaluates the accuracy of state-of-the-art CNA callers using whole-genome sequencing by comparing their ploidy estimates with gold-standard ploidy measurements derived by flow cytometry (FC). We demonstrate that CNA callers are up to 38% inaccurate in cancers with complex genomes, which impacts the accurate estimation of copy number of cancer genes and could have clinical implications and impacts on inferences of tumor evolution. Critically, flow-cytometry-based calibration of CNA callers yields highly accurate ploidy estimates ($\rho_{Pearson} = 0.92$, $p < 0.001$), providing a robust solution to the substantial inaccuracies that compromise clinical decision-making and evolutionary inference in complex cancers.

INTRODUCTION

Tumors are complex tissues comprised of multiple tumor subclones, extracellular matrix components, vasculature, stromal cells, and immune cells. DNA extracted from bulk tumor tissue therefore represents a heterogeneous mixture of DNA from tumor and non-neoplastic cells.[1] Ploidy can be defined as the total DNA content of a cell, expressed as the number sets of 23 chromosomes, otherwise known as genome equivalents (N), such that a normal, diploid cell will have 2N ploidy. By contrast,

© 2026 The Author(s). Published by Elsevier Inc.
This is an open access article under the CC BY license (http://creativecommons.org/licenses/by/4.0/).

many tumor cells have an abnormal number of chromosomes or parts of chromosomes and are defined as being aneuploid.[2] In bulk tissue, the tumor ploidy is the average ploidy across all tumor cells and can range from >1N for near haploid cells to >5N for some tumors. Tumor ploidy is a critical factor in the evolution of cancer. It serves as a predictor of tumor behavior and is both a prognostic[3,4] and diagnostic biomarker.[5]

Tumor ploidy can be determined from genome-wide copy-number aberration (CNA) profiles through DNA sequencing or copy-number microarrays and presented as the average number of chromosomal copies. Accurate copy-number and DNA ploidy estimation is essential for downstream analyses of cancer genomes, including estimating tumor purity in bulk samples, subclonal reconstruction, evolutionary life histories of cancer, driver gene identification, copy-number mutational signatures, and gene dosage.[6–8]

Whole-genome doubling (WGD), the duplication of a cell's chromosomal content, is associated with aggressive tumor behavior and poor prognosis in many cancers.[9,10] In DNA-sequencing studies, WGD estimation commonly relies on the determination of genome-wide copy-number profiles, which provide different measures for WGD inference, including tumor DNA ploidy, the fraction of the genome with loss-of-heterozygosity (LOH),[11] the mode of the major allele,[12] or the minimum event distance from a diploid state.[13]

Though CNA analysis for the assessment of WGD using next-generation sequencing has recently painted the landscape of WGD,[11,14,15] literature published over a decade ago had already demonstrated that ploidy estimation derived *in silico* from CNA profiles, albeit from single-nucleotide polymorphism arrays, is often incorrect (20%–30%).[16,17] More recent benchmarking studies have confirmed that CNA-based approaches remain inconsistent, with performance varying across methods and datasets.[18] This is largely due to the existence of multiple mathematically equivalent solutions for ploidy, which could match different tissue purities.[6] For example, a diploid sample could be equally well modeled at any multiple of ploidy 2N. Indeed, attributing reads as coming from N tumor copies in a fraction ρ of tumor cells is mathematically equivalent to attributing them as coming from N*2 tumor copies in a fraction of $\rho/(2*(1-\rho))$ of tumor cells. These equivalent solutions are two times the ploidy away from one another, that is one WGD event apart.

There are heuristic methods that are sometimes used to manually review ploidy solutions from CNA callers,[11] which is a time-consuming task, such as matching purity estimates from single-nucleotide variants (SNVs) in balanced regions,[19] the absence of non-integer copy-number states or presence of subclonal events at 50% cancer cell fraction, as well as the impossibility of anti-phasing SNVs in haploid regions.[11] However, there is no definitive *in silico* method to identify reliably inaccurate ploidy estimates. This becomes particularly challenging in genomically complex tumors or those with large sub-clonal populations.

Others, including ourselves, have compared *in silico* ploidy estimates to experimental ploidy estimations and have found similar to published discrepancy rates.[12,20,21] Despite this evidence, most cancer genomic studies do not use orthogonal methods to determine tumor DNA ploidy when using *in silico* methods, particularly in cancers that demonstrate complex genomes.

Experimental ploidy analyses, such as flow cytometry (FC) or image cytometry (IC), coupled with DNA staining, provide ground truths. The stoichiometric DNA-binding dyes used in these experiments allow high reproducibility and high-throughput capability.[22–27] These ploidy measurements have been used to benchmark *in silico* estimates in previous studies but could also be leveraged more systematically to inform *in silico* analyses by correcting ploidy fits from CNA callers.[12,20,28]

Here, starting from whole-genome sequencing (WGS) data of chromosomally complex tumors, we first demonstrate that ploidy estimation by various CNA callers is inherently prone to disagreement and error when compared with experimental ploidy measurements. Second, we show that classical "hints" in the data used to correct ploidy are unreliable, and these incorrect ploidy calls lead to substantial differences in downstream analyses that are reliant on CNA profiles. Third, applying our method to different cancer types, we identify WGD and tetraploidy in genomically simple tumor genomes ("diploid-like"), which would not have been identified by sequencing alone. Finally, we propose and benchmark a method of accurate ploidy estimation leveraging clinical formalin-fixed paraffin-embedded (FFPE) cancer samples (surplus to diagnostic requirement) using FC—a relatively inexpensive technique available in most biomedical research facilities and clinical diagnostic laboratories. In the future, this method could be used systematically to identify reliable and correct ploidy estimates obtained through analysis of DNA-sequencing data.

RESULTS

High rates of disagreement in ploidy estimation between three state-of-the-art CNA callers

To assess the concordance of ploidy estimation between three state-of-the-art CNA callers, the outputs of Battenberg, PURPLE, and FACETS, were compared across 51 USARC and 53 high-grade bone tumors (HGOS) where WGS had been performed (Figures 1A and 1B; Star Methods). These tumor types show extreme genome complexity and demonstrate high levels of WGD and chromothripsis.[12,20]

In USARC, concordance was highest between PURPLE and Battenberg ($\rho_{Pearson}$ = 0.34; p = 0.022), with 39/51 samples (76%) showing concordant estimates. For concordant samples, the copy-number segmentation was consistent across CNA callers (Figure 1C). Among 12 discrepant cases, PURPLE predicted seven samples with ploidy <2N, including six with near-haploid content (<1.5N), whereas Battenberg predicted only two such samples. PURPLE also predicted WGD in 73% (37/51), vs. 84% (43/51) by Battenberg.

FACETS showed lower concordance with Battenberg ($\rho_{Pearson}$ = 0.18; p = 0.25) and PURPLE ($\rho_{Pearson}$ = 0.05; p = 0.725), primarily due to over-segmentation. Modifying FACETS settings improved ploidy modeling in 46/51 samples by reducing hyper-segmentation, although at the cost of segmental resolution.

As expected, most USARC samples (9/12) with disagreement between Battenberg and PURPLE corresponded to

CellPress
OPEN ACCESS

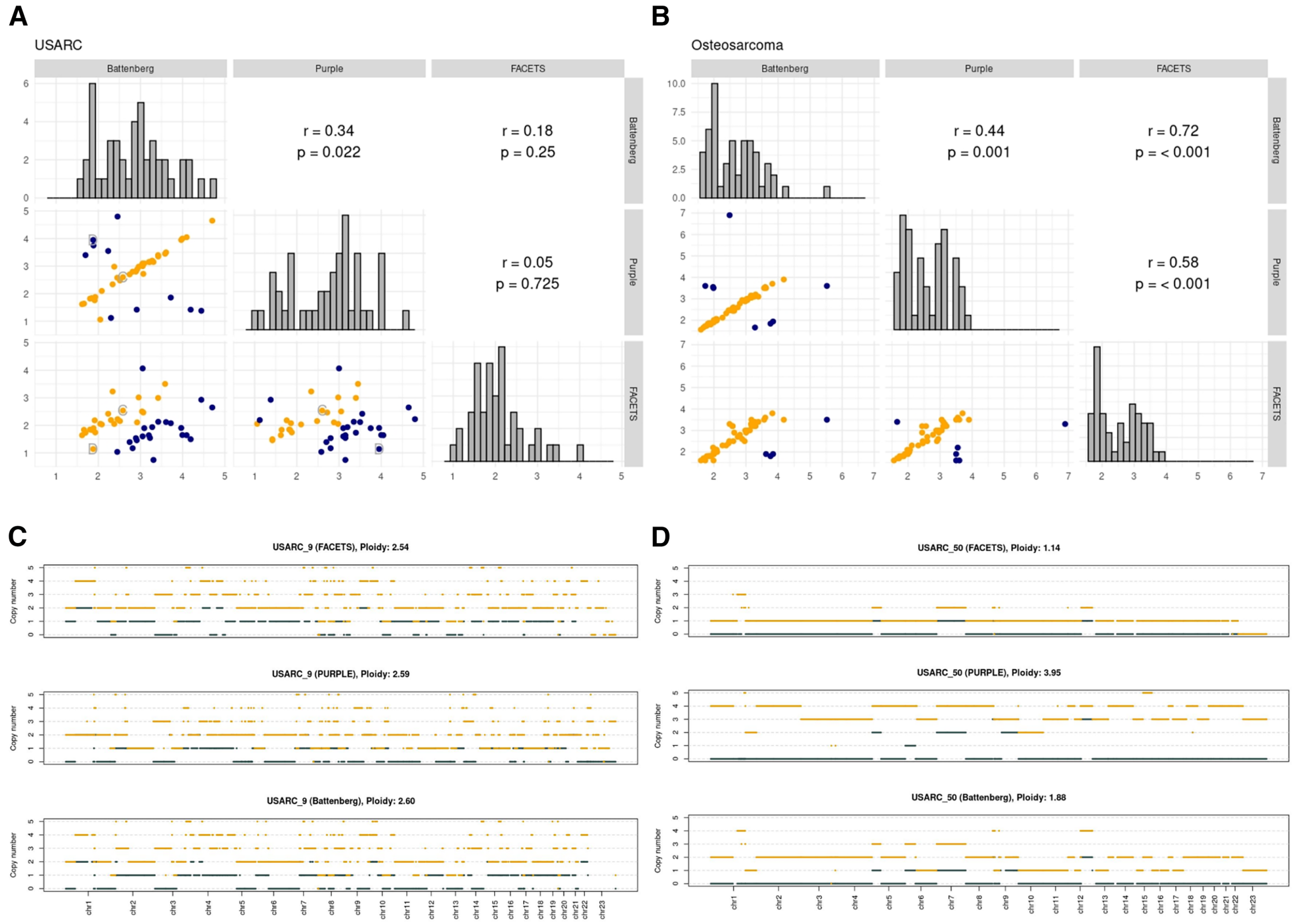

Figure 1. Comparison of ploidy estimates from three CNA callers in USARC and high-grade OS samples

(A and B) Pairplots of USARC and high-grade OS samples comparing ploidy estimates from three copy-number callers: Battenberg, Purple, and FACETS. Scatterplots show pairwise correlations, orange points indicate concordant ploidy calls (<1.0N difference), and blue indicate discrepancies (>1.0N). Diagonal histograms show ploidy distributions per algorithm; upper-right triangle shows Pearson correlation coefficients.

(C and D) Example of USARC cases with concordant (C) and discordant (D) ploidy estimates across algorithms.

two local ploidy/purity optima and differed by almost exactly a factor of two, thus biologically different by one WGD (Figure 1D). In two cases, the difference equated to two WGD events. This suggests the discrepancy mostly stems from the selection of optimal purity/ploidy solutions rather than inconsistencies in fitting copy-number segments. However, for USARC-35, the discrepancy was not limited to a factor of two, suggesting another source of ambiguity, potentially the BAF/LogR segmentation or interpretation of subclonality. To investigate this, the genome was divided into 1Mb bins, and the weighted mean BAF per segment was calculated. While there were no large differences in BAF (USARC-35 mean absolute BAF difference: 14.0% vs. overall mean across samples: 9.9% ± 5.6%), PURPLE had hyper-segmented USARC-35 into 3,539 segments, compared with just 237 segments by Battenberg (Figure S1). Given the biological implausibility of such fine-scale oscillations in copy number across the entire genome, the discrepancy likely reflected an artifact rather than genuine biology. In HGOS, Battenberg and FACETS showed the strongest agreement ($\rho_{Pearson}$ = 0.72; p < 0.001), followed by PURPLE and FACETS ($\rho_{Pearson}$ = 0.58; p < 0.001), and then PURPLE and Battenberg ($\rho_{Pearson=}$0.44; p = 0.001). FACETS default settings remained appropriate for HGOS due to its relatively less complex/segmented genome.

The observed discrepancies in ploidy estimation among the three CNA callers highlight the inconsistencies in estimating tumor ploidy using WGS data and the value of using orthogonal methods such as FC to inform WGS-based CNA profiling. These discrepancies between CNA callers underscore the limitations of WGS alone for ploidy estimation.

Optimization of FC as a method to measure DNA content

FC for DNA ploidy measurement is well established.[24,27,29,30] However, its systematic use to correct ploidy estimates from CNA callers is not. Therefore, we benchmarked three important parameters of FC, i.e., stoichiometry of the stain with DNA content, the impact of staining duration on the ploidy estimates, and the impact of the concentration of nuclei.

First, we validated whether mean fluorescence intensity (MFI) of DAPI is linearly proportional to DNA content in FFPE samples. For this, we identified human testis as an ideal benchmark as it contains haploid (mature sperm), diploid (spermatogonia, stromal), and tetraploid (primary spermatocytes, mitotic cells) populations. We performed FC on three FFPE sections of testis on separate occasions and validated that the MFI of each peak

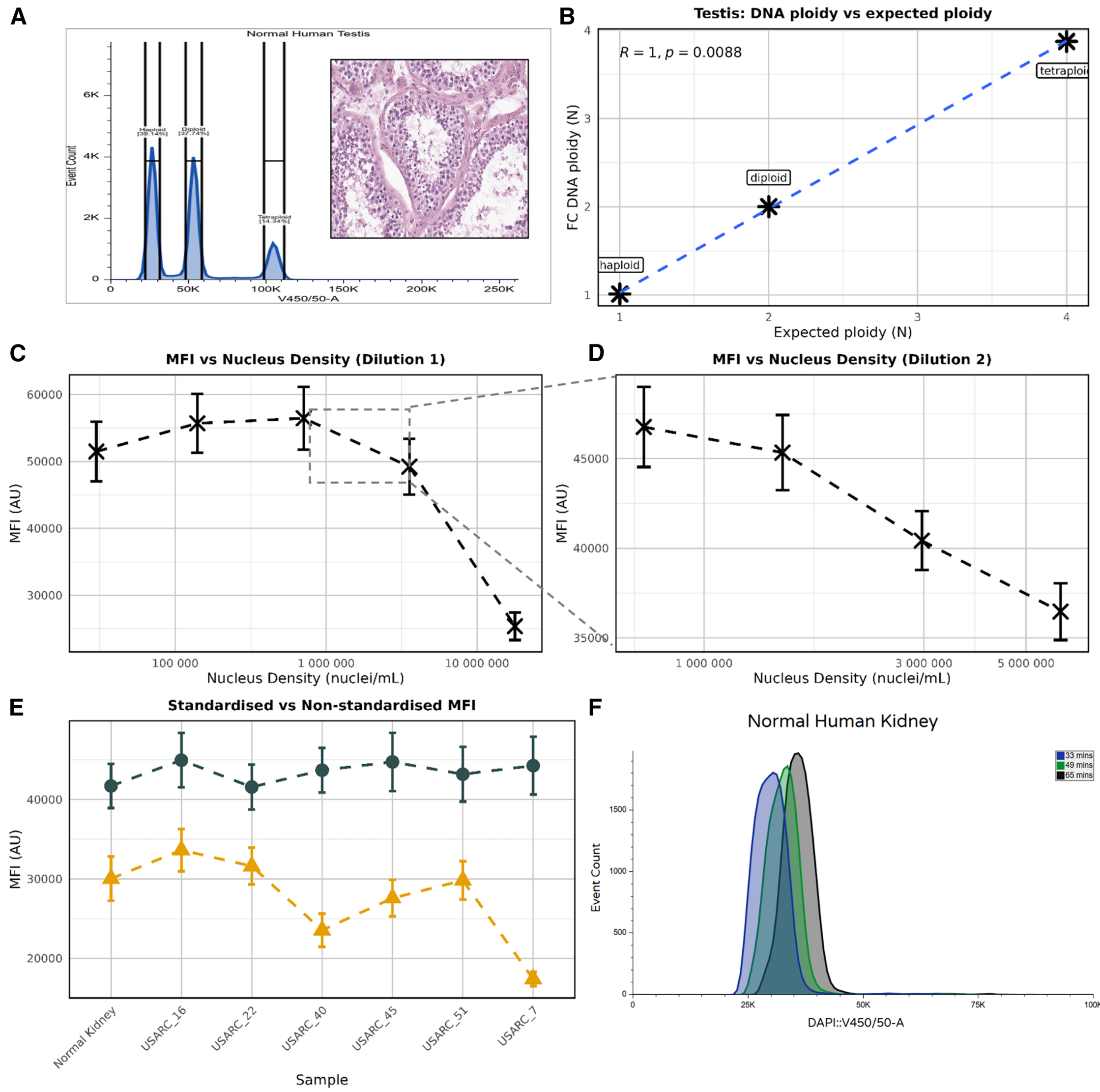

Figure 2. Validation of FC for DNA ploidy estimation using non-neoplastic human tissues

(A) DNA ploidy histogram of FFPE non-neoplastic human testis with embedded H&E image; peaks correspond to haploid, diploid, and tetraploid content.

(B) Using diploid MFI as a reference, the linear regression of MFI across these populations in three samples shows strong correlation with DNA content ($R^2 = 1$, $p < 0.001$) and a small loss of stoichiometry in the tetraploid population (predicted ploidy = 3.87N vs. expected ploidy of 4.0N).

(C) MFI of FFPE normal kidney across serial nucleus dilutions (approx. 17,000,000 to 35,000 nuclei/mL); error bars represent single sample standard deviation.

(D) MFI across a narrower dilution range (approx. 5,000,000 to 500,000 nuclei/mL).

(E) MFI of internal diploid control populations in FFPE tumor samples vs. normal kidney external control, grouped by standardized (approx. 1.5M nuclei/mL, blue) and non-standardized (red) densities; standardized samples show improved concordance between controls.

(F) MFI of FFPE kidney stained for increasing durations; MFI rises slightly with time, but G1/G2-M proportionality remains stable.

was directly proportional to expected DNA content using the diploid peak as a reference ($r^2 = 1.0$, $p < 0.001$) (Figures 2A and 2B). There was only a very small loss of stoichiometry in the tetraploid peak with a mean ploidy of 3.87N.

Second, to assess the effect of DAPI saturation on tumor fluorescence, a diploid control was stained with the same volume of DAPI across serial dilutions (15,000,000 to 30,000 nuclei/mL). Single nuclei suspensions showed reduced MFI with increasing nuclei density (Figures 2C and 2D). To reliably differentiate internal diploid control peaks from tumor peaks, the MFI of external and internal controls needed to closely match. Standardization of nuclei density was trialed in a subset of USARC tumor samples. After dissociation, half of each sample was standardized, and the other half was unadjusted. MFI of internal diploid peaks was more consistent in standardized samples (Figure 2E).

Last, we tested whether staining duration could increase MFI. To assess this, normal human kidney underwent FC at three different time points after staining. MFI increased at increasing stain duration (Figure 2F). The effect of staining time is important when comparing the MFI of the external control to the internal

controls. External diploid controls were run before and after tumor samples to control for added staining time during processing.

FC ploidy estimates were also benchmarked against image cytometry (IC)[31] in a subset of 40 USARC and OS. There was a very strong correlation between the ploidy measurements of the primary tumor population by the two modalities ($\rho_{Pearson}$ = 0.88, $p < 0.001$), with just 4/40 samples showing discrepant results (Figure S2).

To test the sensitivity of FC-based ploidy assessment in low-purity and heterogeneous samples, *in silico* mixing experiments were performed using experimentally derived FCS files from tumors with known WGD populations. Synthetic mixtures were generated by varying tumor and diploid cell fractions, as well as the relative proportions of distinct tumor populations. Across these simulations, tumor-associated DNA-content peaks remained detectable at low tumor fractions, including peaks representing approximately 5% of gated events in mixed samples (Figures S3–S6). In a separate haploid spike-in experiment, a simulated sub-diploid tumor population representing as little as 1% of total ungated events was identifiable both visually and by automated peak detection (Figure S7). Although some minor peaks were partially obscured by debris tails or neighboring populations, they remained discernible. These findings indicate that FC-based ploidy assessment is sensitive to WGD, subclonal ploidy states, and small sub-diploid populations, even in samples with low tumor purity or substantial intratumoral heterogeneity.

In conclusion, our benchmarking shows that FC of FFPE tumor tissue provides accurate DNA ploidy measurement if nuclei density and stain timing are standardized.

Discrepancy between WGS-derived ploidy and experimental ploidy

After establishing our FC ploidy protocol, we set out to compare ploidy estimation between FC and CNA callers. FC was performed on 53 HGOS and 47 USARC for which WGS was available (see STAR Methods), and ploidy estimates from FC were compared with ploidy estimates from a state-of-the-art CNA caller, i.e., Battenberg.[28] Thirty-two estimates were discrepant due to either under- or overestimation of ploidy by the CNA caller (Figures S8A and S8B). A third type of discrepancy was identified, in which the major (largest) tumor population by FC was discrepant from the WGS-derived tumor ploidy estimate. However, an additional, smaller subclonal population was also identified on DNA content histogram, which matched the WGS-estimated ploidy (Figure S8C). As the largest tumor population ploidy did not match the WGS estimate, this was deemed as a discrepancy between FC and WGS; this phenomenon was observed in 16.5% of total samples (21/127).

Of the HGOS samples, 28% (15/53) showed discrepant ploidy estimates between Battenberg and FC (Figures 3A and 3C). Of these, 10/15 were underestimated, and 5/15 were overestimated *in silico*. In both groups, ploidy had been misestimated by almost exactly a factor of two, i.e., a WGD event, indicating a systematic error in calling ploidy resulting in incorrect WGD estimation. Notably, four discrepant samples showed a small subclonal tumor population by FC consistent with the WGS-derived ploidy; these lower ploidy populations were considered putative ancestral populations.

Similarly, 38% (18/47) of USARC samples showed discrepancies between Battenberg and FC (Figure 3A). Just 3/18 were overestimated, compared with 15/18 underestimated *in silico*. Two of these had a small ancestral population on FC matching the WGS-derived ploidy (example shown in Figure S8C).

Comparisons using PURPLE and FACETS were also made. Battenberg and PURPLE had similar discrepancy rates of 38% and 34% in USARC and 29% and 35%, respectively, in HGOS. FACETS showed a higher discrepancy (70% in USARC and 31% in HGOS). Given Battenberg's relatively higher agreement with FC, further analyses focused on this comparison. Interestingly, 12/18 miscalled USARC samples by Battenberg were also miscalled by PURPLE. This suggests shared factors behind incorrect calls, either technical (e.g., similar methodologies favoring lower ploidy solutions) or biological (e.g., subclonal copy-number events in 50% of cells[16]).

Altogether, this shows that while the majority of ploidy estimates from CNA callers agree with ground-truth FC estimates, a substantial proportion do not and that part of the discrepancies is algorithm-specific, while others might be common due to shared technical or biological aspects. This highlights the need for experimental ground-truth ploidy estimates.

Correction of WGS-derived ploidy using experimental ploidy validation

Having obtained the ground-truth ploidy from FC, we reran Battenberg with updated ploidy estimates on the discrepant samples (18/47 USARC; 15/53 HGOS). Ploidy estimates might slightly differ between FC and WGS for two reasons: first, there are sources of noise in both estimates; second, the nuclei used for the FC ploidy estimation and WGS came from different sections of tissue. Therefore, we fed the CNA algorithm a ploidy range around the FC measurement rather than restricting to the exact ploidy.

Following correction, there was a strong correlation between corrected Battenberg ploidy and FC in the USARC and HGOS datasets ($\rho_{Pearson}$ = 0.92 and 0.95, respectively; $p < 0.001$) (Figures 3B and 3D). Only one USARC sample (USARC-26) could not be refitted to a copy-number state aligning with FC. A reason for this may lie in the tumor's subclonality. Using Battenberg, the sample showed a large clonal proportion of genome altered (PGAClonal) (58%), while FC revealed a large subclonal population with lower ploidy (2.73N) compared to the largest population (5.21N). This difference may have caused Battenberg to originally fit a solution between subclones.

Within the HGOS dataset, four samples could not be refitted to a ploidy concordant with FC. OS-47 was diploid by FC but appeared aneuploid (2.9N) by Battenberg, likely due to tissue heterogeneity between sequencing and FC. OS-17 and OS-33 were diploid by FC but had Battenberg ploidies of 2.5N and 2.6N; image cytometry revealed very small >5N subclones, undetected by FC, possibly skewing Battenberg estimates. OS-66 was refit from 3.0N to 6.2N; FC identified a major 4.9N population and a minor 2.8N subclone, possibly explaining the initial intermediate Battenberg call.

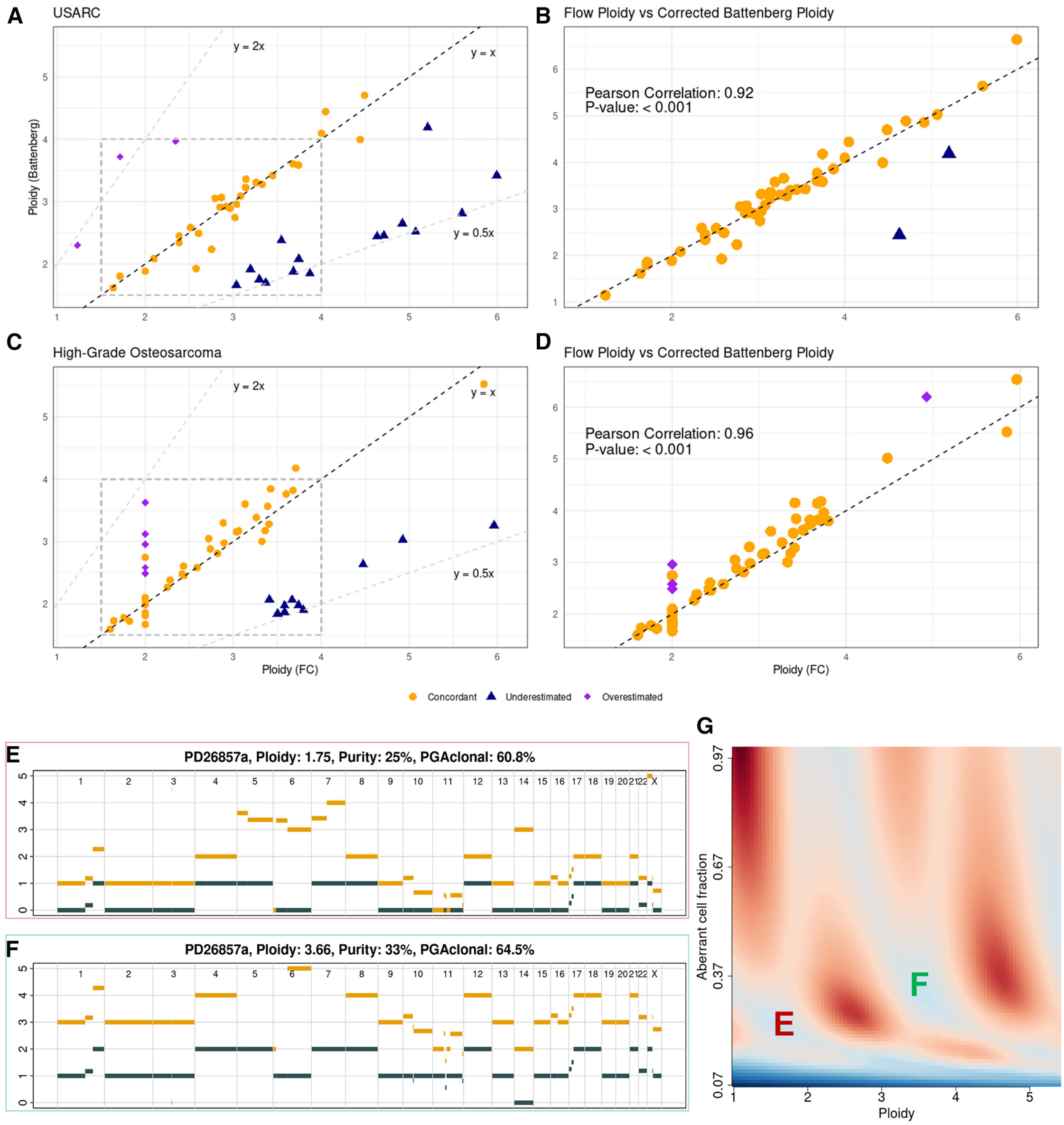

Figure 3. Correlation and correction of *in silico* ploidy estimates using FC

(A) USARC: correlation between FC-derived and Battenberg-derived tumor ploidy estimates. A 38% discrepancy was observed: 18/48 cases underestimated (navy) and 3/48 overestimated (pink) by Battenberg.

(B) Same samples after ploidy correction using FC. Pearson correlation co-efficients were used.

(C) Osteosarcoma: correlation between FC and Battenberg ploidy estimates shows a 28% discrepancy; 10/53 underestimated (navy) and 5/53 overestimated (pink).

(D) Same OS samples post-FC correction. Pearson correlation co-efficients were used.

(E and F) Example of USARC case where Battenberg initially underestimated ploidy (red box), corrected using FC-derived estimate (green box).

(G) Corresponding sunrise plot showing ploidy/purity optima; letters indicate (E) and (F).

FC estimates enabled correction of WGD status in the tumor datasets (Figures 4A and 4B). In USARC, the majority of tumors demonstrated additional WGD events post-correction and were concordant with the histological analysis. The correction of WGD states in the HGOS aligned with expected biological behaviors. Of seven HGOS originally mislabeled as non-WGD, six were high-grade central OS and one a grade 2 extra-skeletal OS.

Alterations in downstream copy-number analysis following ploidy correction

Ploidy correction not only changes the baseline number of copies genome-wide; it can also make a substantial difference to the subclonal copy-number profile of the tumors (Figure 4C), which therefore could lead to important changes in downstream analyses and drastically different interpretations of the CNA

CellPress
OPEN ACCESS

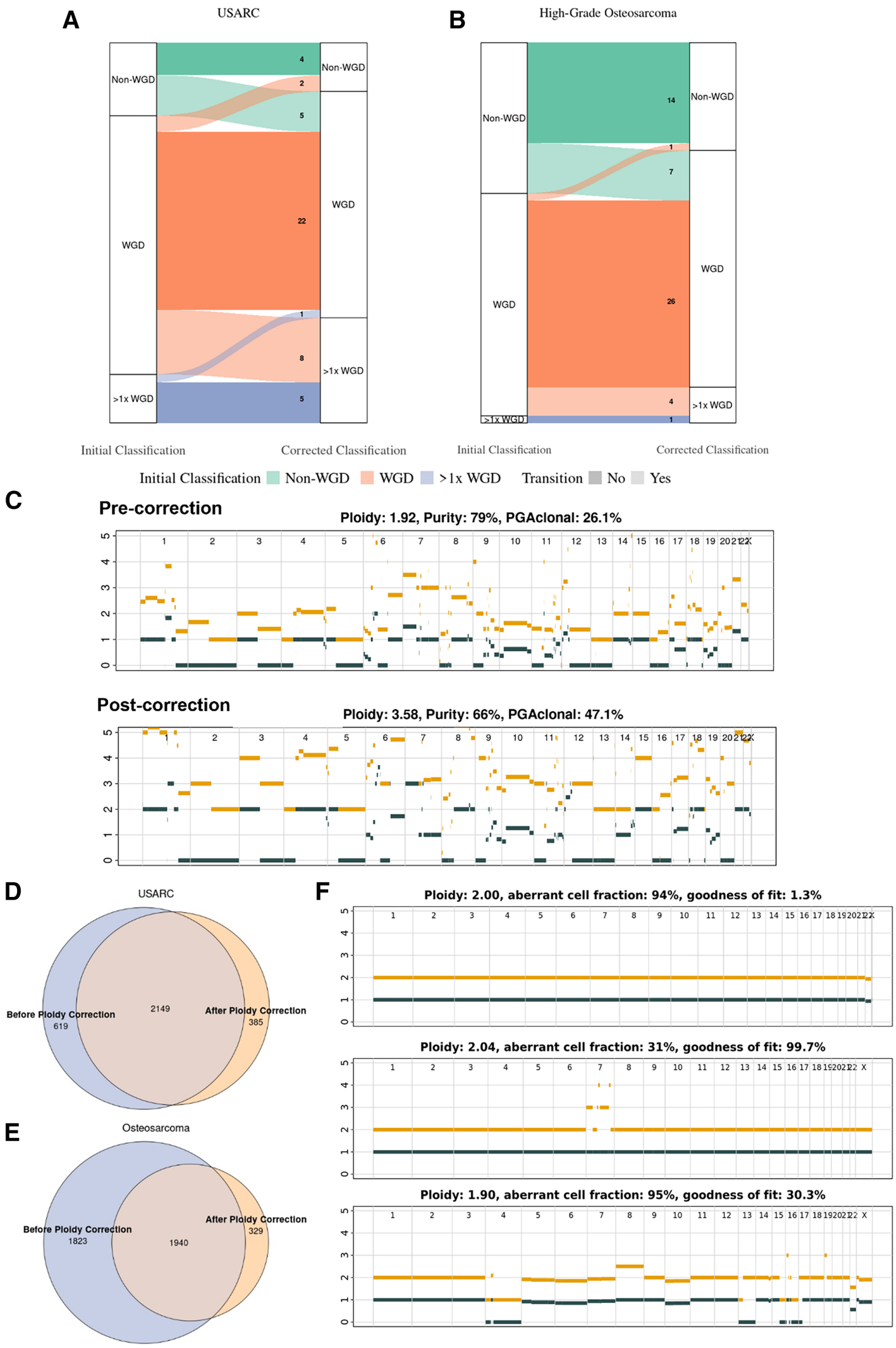

Figure 4. Impact of ploidy correction on WGD classification and subclonal copy-number estimation

(A and B) WGD status of USARC and OS samples before and after FC-based ploidy correction; 16/47 and 12/53 cases changed classification respectively.

(C) Example showing how ploidy correction alters subclonal copy-number estimation in Battenberg, with clonal PGA more than doubling post-correction.

(D and E) Venn diagrams showing GISTIC-identified amplifications and deletions before and after ploidy correction in USARC (D) and OS (E). Prior to correction, 620 (USARC) and 1,823 (high-grade central OS) unique events were detected; correction uncovered 385 (USARC) and 329 (OS) additional unique recurrent events.

(F) Copy-number plots generated by Battenberg for tetraploid LGFMS, tetraploid SS, and WGD SFT (top to bottom). The plots show relatively "quiet" genomes with near-diploid DNA content.

profiles of a cohort. For example, in HGOS OS-20, the ploidy correction helped correctly determine regions of known amplifications in cancer genes such as *MYC* and *MET* that were otherwise missed[32,33] and have both prognostic and therapeutic relevance.

To assess the effect of the ploidy correction on downstream genomic analysis of tumors in a more systematic way, we chose the GISTIC2 module, which is often used to identify recurrent chromosome and gene amplifications and deletions in cancer. We ran the module across the USARC and high-grade central OS datasets before and after correction (Figures 4D and 4E; Figures S9 and S10). In the USARC dataset, before correction, 620 genes were identified as recurrently altered, which were not identified after correction. After correction, 385 additional genes were correctly identified, including *CDK3* (amplification) and *DCLRE1C/Artemis* (deletion), which are significant in other cancer types but not previously highlighted in USARC.[34,35] Similarly, in the HGOS dataset, 1,823 alterations were initially misclassified, and correction revealed 329 additional changes. Altogether, this underscores the necessity of ploidy correction for downstream genomic analyses.

In silico methods to correct erroneous ploidy estimates may be unreliable

There are hints in CNA profiles that are commonly used to manually refit the ploidy from CNA callers.[36] First, large copy-number segments at half-integer values are often used to flag incorrect ploidy estimates, as doubling the profiles would make those half-integer values become integer, i.e., the expected values of clonal states. We tested this in the USARC dataset by comparing the proportion of half-integer segments in Battenberg profiles before and after *in silico* ploidy correction using FC. In 9/17 cases, correction increased the proportion of half-integer segments (Figure S11). This demonstrates that this metric is unreliable for refitting ploidy in USARC, likely due to genome complexity and high subclonality.

Second, CNA-based ploidy estimates are associated with a purity value. Purity can also be obtained from the cancer cell

fraction of clonal SNVs, allowing comparison of purity estimates to flag bad CNA fits. We thus compared the purity attached to our ploidy estimates before and after correction. This revealed that this method is unreliable because (1) SNV-based purity estimates are themselves noisy and (2) different ploidies often produce nearly identical purity and CCF distributions (e.g., case USARC-47 in Figures S12A and S12B; see also Figures 3E, 3F, and4C). Thus, while this method might help choose between ploidy estimates in some cases, it will rarely guarantee the correct solution.

Given the challenges associated with *in silico* ploidy refitting, we assessed the accuracy of expert-derived CNA-based ploidy estimates. Two experienced reviewers in CNA analysis independently refit Battenberg ploidy estimates for the USARC dataset, blinded to experimental ploidy measurements. Interobserver agreement between the specialists was low to moderate [ICC(3,k) = 0.052, p = 0.007], highlighting inconsistency in manual ploidy refitting for chromosomally complex tumors (Figure S13). Furthermore, the mean discrepancy between specialist estimates and FC was higher than that of the original Battenberg calls (51% vs. 38%).

Taken together, these findings suggest that heuristic "hints" in CNA profiles used for manual refitting can be unreliable and may not improve overall accuracy.

Ploidy estimates in genomically "quiet" tumors

Another biological "hint" for choosing between ploidy solutions comes from genome complexity. "Quiet" genomes, characterized by the presence of few CNA, are typically assumed to be diploid.[11,17,37] However, since ploidy is typically estimated *in silico*, this assumption is rarely validated. To test for misclassified, genomically quiet tumors, we compared FC ploidy with Battenberg for 15 translocation-associated tumors including four synovial sarcomas (SSs), nine low-grade fibromyxoid sarcomas (LGFMSs), and two solitary fibrous tumors (SFTs). Surprisingly, 3/15 (20%) Battenberg calls consistent with diploidy were discrepant by FC (Figure S14). Strikingly, one SS and one LGFMS showed near-tetraploidy with FC ploidies of 3.8N and 3.9N, vs. original Battenberg estimates of 2N. Their copy-number profiles were almost "silent," with no other indication of incorrect fitting. One SFT had an FC ploidy of 3.4N vs. 1.9N by Battenberg, with evidence of post-WGD chromosomal loss (Figure 4F).

Histopathologist review revealed that all three cases appeared to have a relatively large nuclei compared to diploid counterparts (Figures S15 and S16). This was confirmed by nuclear segmentation on WSI, which showed increased mean nuclear sizes: SS, 46.7 μm^2; LGFMS, 57.0 μm^2; and SFT, 45.1 μm^2, compared to 11 diploid controls (overall mean: 40.7 μm^2). Notably, one diploid SS had particularly large nuclei (56.7 μm^2). The findings reveal hidden tetraploid tumors with unexpectedly quiet CNA profiles, which highlights a common recurrent blind-spot in cancer genomics studies relying solely on *in silico* ploidy estimation.

DISCUSSION

Our results demonstrate that *in silico* ploidy estimates, generated by state-of-the-art computational methods, can be wrong in up to 38% samples.

CNA fitting is inherently ambiguous, yielding multiple equivalent local optima for ploidy and purity. Because these optima are exactly one or multiple WGDs apart, this technical ambiguity in ploidy fitting leads to profound differences in biological interpretation. Furthermore, fitting at incorrect ploidy in turn leads to inaccurate purity estimates,[6] another important factor in many downstream analyses.[38] Although this is already recognized, most cancer genomics studies persist on relying on these estimates sometimes using additional "hints" in the data to refit ploidy. We show that these hints may not consistently improve accuracy, an exemplar being the biological assumption—almost dogma—that quiet genomes, typically fusion-driven sarcomas, are typically diploid, which we have shown is not always true.

We observed large discrepancies between three commonly used CNA callers and between their outputs and experimental ploidy. However, CNA callers can yield accurate estimates of ploidy and WGD status if supplemented with experimental validation.

Overall, these findings have profound clinical and experimental implications. Inaccurate ploidy estimation can substantially affect downstream analyses, as seen in our GISTIC results. It may also lead to underestimation of WGD events, which are increasingly recognized as prognostic markers. TCGA data suggest WGD is linked to poor prognosis in most cancers, though it may be beneficial in some, like bladder carcinoma.[39] With error rates up to 38%, the true role of WGD and its clinical relevance remains uncertain. We recommend pairing genomic analyses with orthogonal experimental ploidy estimates wherever possible, especially in cancers with complex genomes.

Correcting WGS-derived ploidy estimates in both USARC and HGOS samples led to a significant shift in their WGD classification. Accurate identification of WGD events enables more robust classification and analysis of tumors. FC analysis further clarified true ploidy heterogeneity, with multiple tumors exhibiting once and twice WGD clones within the same sample. While Battenberg can detect subclonal copy-number states, it does not model states more than one copy number apart from the clonal population, making cytometric analysis essential for identifying true ploidy. Without it, bioinformatic interpretations may be flawed. The data show that the copy-number profile of the discrepant samples can undergo extensive changes post-correction. This has important implications for the downstream analysis of these tumors, particularly regarding their subclonal deconvolution and reconstruction into phylogenetic trees. A lack of accurate ploidy data also limits the ability to form accurate conclusions related to genomic instability and WGD in these tumors and ultimately leads to the misinterpretation of cancer evolution and progression.

Recent evidence has demonstrated that WGD is a common event (25%) in alveolar rhabdomyosarcoma,[40] originally reported as being tetraploid 20 years ago in FC studies.[41,42] Here, we expand the subtypes of fusion-driven sarcomas in which WGD is present.[43–45] The identification of WGD in these fusion-driven sarcomas is complicated by their frequently "chromosomally quiet" genomes that bioinformatically show no indication of WGD. The implication is that WGD will be consistently missed by *in silico* analysis alone. The potential impact is that current genomic studies in rarer sarcomas may

be neglecting important oncogenic pathways in a proportion of cases.

Our results support the accuracy of FC in measuring DNA ploidy. Formalin fixation affects many nuclear dyes, but DAPI is minimally impacted and requires no tissue pretreatment. Further, while chromatin condensation can reduce dye binding,[25–27] DAPI is relatively unaffected. Standardizing nuclei density and stain timing was key to this protocol's reliability.

As genomic research shifts increasingly toward single-cell DNA and RNA sequencing studies to gain more granular insights than with bulk WGS, the old issues of discrepancy are likely to persist as the underlying ploidy and purity modeling algorithms remain largely the same in the single-cell analysis pipelines. In these situations, FC remains crucial for accurately classifying and studying single tumor cells. Ultimately, as spatial profiling on tumors becomes more informative, driving spatial studies to become more widespread, FC may become limited in its capacity to assess ploidy. Addressing these new challenges will call for computational algorithms to accurately assess tumor ploidy at a regional level while preserving spatial information.

Limitations of the study

A key limitation of using cytometry in correcting WGS-derived ploidy is that the cells undergoing sequencing are not the same as those undergoing FC. Our results assume that closely related tissues are more likely to share genomic features. But the experimental ploidy may not represent precisely the sequenced tissue, and this difference could potentially extend as far as a WGD event. Another limitation is the presence of large subclonal populations within some tumors. Current CNA algorithms model subclones at similar ploidy and cannot account for substantially different ploidy states. Forcing the algorithms to model the ploidy based on the largest tumor population may seem reasonable but still risks introducing errors in downstream analysis.

RESOURCE AVAILABILITY

Lead contact

Further information and requests should be directed to lead contact, Prof. Nischalan Pillay (n.pillay@ucl.ac.uk).

Materials availability

This study did not generate new unique reagents.

Data and code availability

- For the USARC dataset, WGS data are deposited in EGA database at EMBL-EBI (https://ega-archive.org/) under accession number EGAD00001004162 as described by Steele et al.[12]
- For the osteosarcoma (OS) dataset, genomic data were obtained from Genomics England and the TCGA/ICGC Pan-Cancer Analysis of Whole Genomes Project (PCAWG) as described by Valle-Inclan et al.[20]
- For translocation-associated sarcoma dataset, genomic data were obtained from Genomics England.[46] Research on the de-identified patient data used in this publication was carried out in the Genomics England Research Environment. Data are available subject to approval; interested researchers may apply via the Genomics England website (https://www.genomicsengland.co.uk).
- Unless otherwise specified, computational tools were run with default parameters.
- Any additional information required to re-analyze the data reported in this study is available from the lead contact upon request.

ACKNOWLEDGMENTS

Funding for this study was received from The Tom Prince Cancer Trust (A.M.F. and N.P.), the Bone Cancer Research Trust infrastructure grants (A.M.F.), RNOH NHS R&D grant (A.M.F.), and the Jean Shanks and Pathological Society Clinical PhD Fellowship grant (T.B.). The project was also supported by the National Institute for Health Research, UCLH Biomedical Research Centre, and the UCL Experimental Cancer Center (N.P. and A.M.F.). I.A.E.M.v.B. and I.C.-C. thank EMBL for funding. N.P. has received funding from Cancer Research UK (award number 18387) and holds a Cancer Research UK Career Establishment award (RCCCEA-Nov23/100003). We are grateful to the Biobank Team at the RNOH and all healthcare workers who cared for the patients. We thank the patients for engaging in our research. This research was made possible through access to data in the National Genomic Research Library, which is managed by Genomics England Limited (a wholly owned company of the Department of Health and Social Care). The National Genomic Research Library holds data provided by patients and collected by the NHS as part of their care and data collected as part of their participation in research. The National Genomic Research Library is funded by the National Institute for Health Research and NHS England. The Wellcome Trust, Cancer Research UK, and the Medical Research Council have also funded research infrastructure. The graphical abstract was created in BioRender. Pillay, N. (2026) https://BioRender.com/7hxf7xv.

AUTHOR CONTRIBUTIONS

T.B., P.V.L., A.M.F., N.P., and M.T. conceptualized the study; T.B., D.O., Y.G., A.M.F., N.P., and M.T. developed the methodology; T.B., D.O., P.T., C.D., R.L., C.C., and M.M. conducted the experiments; A.T. and F.O. curated and prepared the FFPE sample materials; T.B., D.O., P.T., I.A.E.M.v.B., J.C., S.D.N., C.D.S., and M.F. performed the formal data analysis; T.B. and D.O. wrote the original manuscript draft; and I.A.E.M.v.B., J.C., D.T., I.C.-C., P.V.L., A.M.F., N.P., and M.T. reviewed and edited the manuscript.

DECLARATION OF INTERESTS

The authors declare no competing interests.

DECLARATION OF GENERATIVE AI AND AI-ASSISTED TECHNOLOGIES IN THE WRITING PROCESS

Generative AI including ChatGPT V5 was used to assist in generating code for the purpose of data analysis and statistics. Where code was generated by AI, it was thoroughly interrogated by the researcher.

STAR★METHODS

Detailed methods are provided in the online version of this paper and include the following:

- KEY RESOURCES TABLE
- EXPERIMENTAL MODEL AND STUDY PARTICIPANT DETAILS
  - Ethics approval
- METHOD DETAILS
  - Buffers and solutions
  - Experimental ploidy measurement by FC and IC in FFPE tissues
  - WGS protocol and data processing
- QUANTIFICATION AND STATISTICAL ANALYSIS

SUPPLEMENTAL INFORMATION

Supplemental information can be found online at https://doi.org/10.1016/j.crmeth.2026.101570.

Received: December 30, 2025
Revised: May 6, 2026
Accepted: July 30, 2026
Published: August 31, 2026

REFERENCES

1. Aguadé-Gorgorió, G., Anderson, A.R.A., and Solé, R. (2024). Modeling tumors as complex ecosystems. iScience *27*, 110699. https://doi.org/10.1016/j.isci.2024.110699.
2. Ben-David, U., and Amon, A. (2020). Context is everything: aneuploidy in cancer. Nat. Rev. Genet. *21*, 44–62. https://doi.org/10.1038/s41576-019-0171-x.
3. Zhao, Z., Zhang, X., Li, Z., Gao, Y., Guan, X., Jiang, Z., Liu, Z., Yang, M., Chen, H., Ma, X., et al. (2021). Automated assessment of DNA ploidy, chromatin organization, and stroma fraction to predict prognosis and adjuvant therapy response in patients with stage II colorectal carcinoma. Am. J. Cancer Res. *11*, 6119–6132.
4. Ersvær, E., Hveem, T.S., Vlatkovic, L., Brennhovd, B., Kleppe, A., Tobin, K.A.R., Pradhan, M., Cyll, K., Wæhre, H., Kerr, D.J., and Danielsen, H.E. (2020). Prognostic value of DNA ploidy and automated assessment of stroma fraction in prostate cancer. Int. J. Cancer *147*, 1228–1234. https://doi.org/10.1002/ijc.32832.
5. Bechstedt, N., Pomjanski, N., Schramm, M., and Remmerbach, T.W. (2022). Evaluation of Static DNA Ploidy Analysis Using Conventional Brush Biopsy-Based Cytology Samples as an Adjuvant Diagnostic Tool for the Detection of a Malignant Transformation in Potentially Oral Malignant Diseases: A Prospective Study. Cancers *14*, 5828. https://doi.org/10.3390/cancers14235828.
6. Tarabichi, M., Salcedo, A., Deshwar, A.G., Ni Leathlobhair, M., Wintersinger, J., Wedge, D.C., Van Loo, P., Morris, Q.D., and Boutros, P.C. (2021). A practical guide to cancer subclonal reconstruction from DNA sequencing. Nat. Methods *18*, 144–155. https://doi.org/10.1038/s41592-020-01013-2.
7. Dentro, S.C., Wedge, D.C., and Loo, P.V. (2017). Principles of Reconstructing the Subclonal Architecture of Cancers. Cold Spring Harb. Perspect. Med. *7*, a026625. https://doi.org/10.1101/cshperspect.a026625.
8. Liu, L.Y., Bhandari, V., Salcedo, A., Espiritu, S.M.G., Morris, Q.D., Kislinger, T., and Boutros, P.C. (2020). Quantifying the influence of mutation detection on tumour subclonal reconstruction. Nat. Commun. *11*, 6247. https://doi.org/10.1038/s41467-020-20055-w.
9. Lambuta, R.A., Nanni, L., Liu, Y., Diaz-Miyar, J., Iyer, A., Tavernari, D., Katanayeva, N., Ciriello, G., and Oricchio, E. (2023). Whole-genome doubling drives oncogenic loss of chromatin segregation. Nature *615*, 925–933. https://doi.org/10.1038/s41586-023-05794-2.
10. Kikutake, C., and Suyama, M. (2022). Pan-cancer analysis of whole-genome doubling and its association with patient prognosis. BMC cancer *23*, 619. https://doi.org/10.21203/rs.3.rs-2282908/v1.
11. Dentro, S.C., Leshchiner, I., Haase, K., Tarabichi, M., Wintersinger, J., Deshwar, A.G., Yu, K., Rubanova, Y., Macintyre, G., Demeulemeester, J., et al. (2021). Characterizing genetic intra-tumor heterogeneity across 2,658 human cancer genomes. Cell *184*, 2239–2254.e39. https://doi.org/10.1016/j.cell.2021.03.009.
12. Steele, C.D., Tarabichi, M., Oukrif, D., Webster, A.P., Ye, H., Fittall, M., Lombard, P., Martincorena, I., Tarpey, P.S., Collord, G., et al. (2019). Undifferentiated Sarcomas Develop through Distinct Evolutionary Pathways. Cancer Cell *35*, 441–456.e8. https://doi.org/10.1016/j.ccell.2019.02.002.
13. Kaufmann, T.L., Petkovic, M., Watkins, T.B.K., Colliver, E.C., Laskina, S., Thapa, N., Minussi, D.C., Navin, N., Swanton, C., Van Loo, P., et al. (2022). MEDICC2: whole-genome doubling aware copy-number phylogenies for cancer evolution. Genome Biol. *23*, 241. https://doi.org/10.1186/s13059-022-02794-9.
14. Zack, T.I., Schumacher, S.E., Carter, S.L., Cherniack, A.D., Saksena, G., Tabak, B., Lawrence, M.S., Zhsng, C.Z., Wala, J., Mermel, C.H., et al. (2013). Pan-cancer patterns of somatic copy number alteration. Nat. Genet. *45*, 1134–1140. https://doi.org/10.1038/ng.2760.
15. Martínez-Jiménez, F., Movasati, A., Brunner, S.R., Nguyen, L., Priestley, P., Cuppen, E., and Van Hoeck, A. (2023). Pan-cancer whole-genome comparison of primary and metastatic solid tumours. Nature *618*, 333–341. https://doi.org/10.1038/s41586-023-06054-z.
16. Van Loo, P., Nordgard, S.H., Lingjærde, O.C., Russnes, H.G., Rye, I.H., Sun, W., Weigman, V.J., Marynen, P., Zetterberg, A., Naume, B., et al. (2010). Allele-specific copy number analysis of tumors. Proc. Natl. Acad. Sci. *107*, 16910–16915. https://doi.org/10.1073/pnas.1009843107.
17. Carter, S.L., Cibulskis, K., Helman, E., McKenna, A., Shen, H., Zack, T., Laird, P.W., Onofrio, R.C., Winckler, W., Weir, B.A., et al. (2012). Absolute quantification of somatic DNA alterations in human cancer. Nat. Biotechnol. *30*, 413–421. https://doi.org/10.1038/nbt.2203.
18. Song, Y., Mei, Z., Zheng, Q., Yuan, Q., Liang, Y., Gao, J., Zhou, L., Wu, S., and Wu, W. (2025). Benchmarking Ploidy Estimation Methods for Bulk and Single-Cell Whole Genome Sequencing. Adv. Sci. *12*, e07839. https://doi.org/10.1002/advs.202507839.
19. Antonello, A., Bergamin, R., Calonaci, N., Househam, J., Milite, S., Williams, M.J., Anselmi, F., d'Onofrio, A., Sundaram, V., Sosinsky, A., et al. (2024). Computational validation of clonal and subclonal copy number alterations from bulk tumor sequencing using CNAqc. Genome Biol. *25*, 38. https://doi.org/10.1186/s13059-024-03170-5.
20. Espejo Valle-Inclan, J., De Noon, S., Trevers, K., Elrick, H., van Belzen, I.A.E.M., Zumalave, S., Sauer, C.M., Tanguy, M., Butters, T., Muyas, F., et al. (2025). Ongoing chromothripsis underpins osteosarcoma genome complexity and clonal evolution. Cell *188*, 352–370.e22. https://doi.org/10.1016/j.cell.2024.12.005.
21. Cortes-Ciriano, I., Steele, C.D., Piculell, K., Al-Ibraheemi, A., Eulo, V., Bui, M.M., Chatzipli, A., Dickson, B.C., Borcherding, D.C., Feber, A., et al. (2023). Genomic patterns of malignant peripheral nerve sheath tumor (MPNST) evolution correlate with clinical outcome and are detectable in cell-free DNA. Cancer Discov. *13*, 654–671. https://doi.org/10.1158/2159-8290.cd-22-0786.
22. Chen, T.L., Luo, I., Mikhail, N., Rasková, J., and Raŝka, K. (1995). Comparison of flow and image cytometry for DNA content analysis of fresh and formalin-fixed, paraffin-embedded tissue in breast carcinoma. Cytometry *22*, 181–189. https://doi.org/10.1002/cyto.990220305.
23. Lee, H., Rabinovitch, P.S., Mattis, A.N., Kakar, S., and Choi, W.-T. (2020). DNA flow cytometric analysis of paraffin-embedded tissue for the diagnosis of malignancy in bile duct biopsies. Hum. Pathol. *99*, 80–87. https://doi.org/10.1016/j.humpath.2020.04.002.
24. Blanco, R., Rengifo, C.E., Cedeño, M., Frómeta, M., and Rengifo, E. (2013). Flow Cytometric Measurement of Aneuploid DNA Content Correlates with High S-Phase Fraction and Poor Prognosis in Patients with Non-Small-Cell Lung Cancer. ISRN Biomarkers *2013*, 1–8. https://doi.org/10.1155/2013/354123.
25. Overton, W.R., and McCoy, J.P. (1994). Reversing the effect of formalin on the binding of propidium iodide to DNA. Cytometry *16*, 351–356. https://doi.org/10.1002/cyto.990160410.
26. Bedner, E., Burfeind, P., Gorczyca, W., Melamed, M.R., and Darzynkiewicz, Z. (1997). Laser scanning cytometry distinguishes lymphocytes, monocytes, and granulocytes by differences in their chromatin structure. Cytometry *29*, 191–196.
27. Hedley, D.W., Friedlander, M.L., Taylor, I.W., Rugg, C.A., and Musgrove, E.A. (1983). Method for analysis of cellular DNA content of paraffin-embedded pathological material using flow cytometry. J. Histochem. Cytochem. *31*, 1333–1335. https://doi.org/10.1177/31.11.6619538.
28. Nik-Zainal, S., Van Loo, P., Wedge, D.C., Alexandrov, L.B., Greenman, C.D., Lau, K.W., Raine, K., Jones, D., Marshall, J., Ramakrishna, M., et al. (2012). The Life History of 21 Breast Cancers. Cell *149*, 994–1007. https://doi.org/10.1016/j.cell.2012.04.023.

29. Darzynkiewicz, Z., and Huang, X. (2004). Analysis of Cellular DNA Content by Flow Cytometry. Curr. Protoc. Im. *5*, 5.7. https://doi.org/10.1002/0471142735.im0507s60.

30. Choi, W.-T., Tsai, J.-H., Rabinovitch, P.S., Small, T., Huang, D., Mattis, A.N., and Kakar, S. (2018). Diagnosis and risk stratification of Barrett's dysplasia by flow cytometric DNA analysis of paraffin-embedded tissue. Gut *67*, 1229–1238. https://doi.org/10.1136/gutjnl-2017-313815.

31. Dunn, J.M., Mackenzie, G.D., Oukrif, D., Mosse, C.A., Banks, M.R., Thorpe, S., Sasieni, P., Bown, S.G., Novelli, M.R., Rabinovitch, P.S., and Lovat, L.B. (2010). Image cytometry accurately detects DNA ploidy abnormalities and predicts late relapse to high-grade dysplasia and adenocarcinoma in Barrett's oesophagus following photodynamic therapy. Br. J. Cancer *102*, 1608–1617. https://doi.org/10.1038/sj.bjc.6605688.

32. Noon, S.D., Ijaz, J., Coorens, T.H., Amary, F., Ye, H., Strobl, A., Lyskjær, I., Flanagan, A.M., and Behjati, S. (2021). MYC amplifications are common events in childhood osteosarcoma. J. Pathol.: Clin. Res. *7*, 425–431. https://doi.org/10.1002/cjp2.219.

33. Zeng, M., Liu, C., Gong, H., Tang, Z., Wen, J., Wang, S., and Xiao, S. (2024). Therapeutic potential of tyrosine-protein kinase MET in osteosarcoma. Front. Mol. Biosci. *11*, 1367331. https://doi.org/10.3389/fmolb.2024.1367331.

34. Parsyan, A., Bhat, V., Athwal, H., Goebel, E.A., and Allan, A.L. (2025). Artemis and its role in cancer. Transl. Oncol. *51*, 102165. https://doi.org/10.1016/j.tranon.2024.102165.

35. Teo, T., Kasirzadeh, S., Albrecht, H., Sykes, M.J., Yang, Y., and Wang, S. (2022). An overview of CDK3 in cancer: clinical significance and pharmacological implications. Pharmacol. Res. *180*, 106249. https://doi.org/10.1016/j.phrs.2022.106249.

36. Consortium, T.I.P.-C.A., of, W.G., Aaltonen, L.A., Abascal, F., Abeshouse, A., Aburatani, H., Adams, D.J., Agrawal, N., Ahn, K.S., Ahn, S.-M., et al. (2020). Pan-cancer analysis of whole genomes. Nature *578*, 82–93. https://doi.org/10.1038/s41586-020-1969-6.

37. Gounder, M.M., Agaram, N.P., Trabucco, S.E., Robinson, V., Ferraro, R.A., Millis, S.Z., Krishnan, A., Lee, J., Attia, S., Abida, W., et al. (2022). Clinical genomic profiling in the management of patients with soft tissue and bone sarcoma. Nat. Commun. *13*, 3406. https://doi.org/10.1038/s41467-022-30496-0.

38. Aran, D., Sirota, M., and Butte, A.J. (2015). Systematic pan-cancer analysis of tumour purity. Nat. Commun. *6*, 8971. https://doi.org/10.1038/ncomms9971.

39. Sanz-Gómez, N., González-Álvarez, M., Rivas, J.D.L., and Cárcer, G. de (2023). Whole-Genome Doubling as a source of cancer: how, when, where, and why? Front. Cell Dev. Biol. *11*, 1209136. https://doi.org/10.3389/fcell.2023.1209136.

40. Nacev, B.A., Sanchez-Vega, F., Smith, S.A., Antonescu, C.R., Rosenbaum, E., Shi, H., Tang, C., Socci, N.D., Rana, S., Gularte-Mérida, R., et al. (2022). Clinical sequencing of soft tissue and bone sarcomas delineates diverse genomic landscapes and potential therapeutic targets. Nat. Commun. *13*, 3405. https://doi.org/10.1038/s41467-022-30453-x.

41. Kilpatrick, S.E., Teot, L.A., Geisinger, K.R., Martin, P.L., Shumate, D.K., Zbieranski, N., Russell, G.B., and Fletcher, C.D. (1994). Relationship of DNA ploidy to histology and prognosis in rhabdomyosarcoma. Comparison of flow cytometry and image analysis. Cancer *74*, 3227–3233. https://doi.org/10.1002/1097-0142(19941215)74:12<3227::aid-cncr2820741223>3.0.co;2-x.

42. Shapiro, D.N., Parham, D.M., Douglass, E.C., Ashmun, R., Webber, B.L., Newton, W.A., Hancock, M.L., Maurer, H.M., and Look, A.T. (1991). Relationship of tumor-cell ploidy to histologic subtype and treatment outcome in children and adolescents with unresectable rhabdomyosarcoma. J. Clin. Oncol. *9*, 159–166. https://doi.org/10.1200/jco.1991.9.1.159.

43. Priestley, P., Baber, J., Lolkema, M.P., Steeghs, N., de Bruijn, E., Shale, C., Duyvesteyn, K., Haidari, S., van Hoeck, A., Onstenk, W., et al. (2019). Pan-cancer whole-genome analyses of metastatic solid tumours. Nature *575*, 210–216. https://doi.org/10.1038/s41586-019-1689-y.

44. Bolli, N., Avet-Loiseau, H., Wedge, D.C., Van Loo, P., Alexandrov, L.B., Martincorena, I., Dawson, K.J., Iorio, F., Nik-Zainal, S., Bignell, G.R., et al. (2014). Heterogeneity of genomic evolution and mutational profiles in multiple myeloma. Nat. Commun. *5*, 2997. https://doi.org/10.1038/ncomms3997.

45. Hames-Fathi, S. (2023). The Immunogenomic Landscape of Soft Tissue Sarcoma (UCL).

46. Prendergast, S.C., Strobl, A.C., Cross, W., Pillay, N., Strauss, S.J., Ye, H., Lindsay, D., Tirabosco, R., Chalker, J., Mahamdallie, S.S., et al. (2020). Sarcoma and the 100,000 Genomes Project: our experience and changes to practice. J. Pathol. Clin. Res. *6*, 297–307. https://doi.org/10.1002/cjp2.174.

47. Hedley, D.W., Shankey, T.V., and Wheeless, L.L. (1993). DNA cytometry consensus conference. Cytometry *14*, 471. https://doi.org/10.1002/cyto.990140502.

48. Cossarizza, A., Chang, H., Radbruch, A., Acs, A., Adam, D., Adam-Klages, S., Agace, W.W., Aghaeepour, N., Akdis, M., Allez, M., et al. (2019). Guidelines for the use of flow cytometry and cell sorting in immunological studies (second edition). Eur. J. Immunol. *49*, 1457–1973. https://doi.org/10.1002/eji.201970107.

49. Rabinovitch, P.S. (1994). Chapter 18 DNA Content Histogram and Cell-Cycle Analysis. Methods Cell Biol. *41*, 263–296. https://doi.org/10.1016/s0091-679x(08)61723-9.

50. Menzel, M., Endris, V., Schwab, C., Kluck, K., Neumann, O., Beck, S., Ball, M., Schaaf, C., Fröhling, S., Lichtner, P., et al. (2023). Accurate tumor purity determination is critical for the analysis of homologous recombination deficiency (HRD). Transl. Oncol. *35*, 101706. https://doi.org/10.1016/j.tranon.2023.101706.

51. Kim, S., Scheffler, K., Halpern, A.L., Bekritsky, M.A., Noh, E., Källberg, M., Chen, X., Kim, Y., Beyter, D., Krusche, P., and Saunders, C.T. (2018). Strelka2: fast and accurate calling of germline and somatic variants. Nat. Methods *15*, 591–594. https://doi.org/10.1038/s41592-018-0051-x.

52. Cibulskis, K., Lawrence, M.S., Carter, S.L., Sivachenko, A., Jaffe, D., Sougnez, C., Gabriel, S., Meyerson, M., Lander, E.S., and Getz, G. (2013). Sensitive detection of somatic point mutations in impure and heterogeneous cancer samples. Nat. Biotechnol. *31*, 213–219. https://doi.org/10.1038/nbt.2514.

53. Hörst, F., Rempe, M., Heine, L., Seibold, C., Keyl, J., Baldini, G., Ugurel, S., Siveke, J., Grünwald, B., Egger, J., and Kleesiek, J. (2024). CellViT: Vision Transformers for precise cell segmentation and classification. Med. Image Anal. *94*, 103143. https://doi.org/10.1016/j.media.2024.103143.

STAR★METHODS

KEY RESOURCES TABLE

REAGENT or RESOURCE	SOURCE	IDENTIFIER
**Biological samples**		
FFPE tissue samples	UCL/UCLH Biobank RNOH Satellite Site	IRAS: 272816 ; UCL/UCLH project no. EC17.14
**Chemicals, peptides, and recombinant proteins**		
Proteinase from *Bacillus licheniformis* (proteinase XXIV)	Sigma-Aldrich	P5380
4′,6-diamidino-2-phenylindole (DAPI)	Thermo Scientific™	62247
2 mM EDTA	Invitrogen	15575-038
Bovine serum albumin	Sigma-Aldrich	A7906-100G
**Deposited data**		
Undifferentiated WGS dataset	EGA at EMBL-EBI as described by Steele et al.[12]	https://ega-archive.org/; EGAD00001004162
Osteosarcoma WGS dataset	Genomics England, and the TCGA/ICGC Pan-Cancer Analysis of Whole Genomes Project (PCAWG) as described in Valle-Inclan et al.[20]	https://www.genomicsengland.co.uk; https://portal.gdc.cancer.gov/
Translocation-associated tumor WGS dataset	Genomics England, as described in Prendergast et al.;[46] available subject to Genomics England approval	https://www.genomicsengland.co.uk
**Software and algorithms**		
Floreada.io	SIMD	https://floreada.io/analysis
FCS Express 7 Research Edition	*De Novo* Software	https://denovosoftware.com/full-access/download-landing/
Battenberg (2.2.10)	Wedge Lab	https://github.com/Wedge-lab/battenberg
AMBER	HMF tools	https://github.com/hartwigmedical/hmftools/tree/master/amber
COBALT	HMF tools	https://github.com/hartwigmedical/hmftools/tree/master/cobalt
PURPLE (3.8)	HMF tools	https://github.com/hartwigmedical/hmftools/blob/master/purple/README.md
FACETS (0.6.2)	MSKCC	https://github.com/mskcc/facets
GISTIC 2.0.3	Broad Institute	https://broadinstitute.github.io/gistic2/
DPClust v2.2.8	Wedge Lab	https://github.com/Wedge-lab/dpclust
CellViT	TIO-IKIM	https://github.com/TIO-IKIM/CellViT
Varscan v2.4.6	Daniel C. Koboldt	https://varscan.sourceforge.net/
**Other**		
EZ-Cytofunnel	Thermo Scientific	A78710004
Corning Cell Strainer	Corning	431751
BD Fortessa ×20 flow cytometer	BD Biosciences	https://www.bdbiosciences.com/en-gb/products/instruments/flow-cytometers
Shandon Cytospin 2	Shandon	Not available
Hamamatsu C4742-95 camera	Hamamatsu Photonics	https://camera.hamamatsu.com/jp/en.html
Fairfield DNA Ploidy system	Fairfield Imaging	N/A
Zeiss Axioplan microscope	Zeiss	https://www.zeiss.com/

EXPERIMENTAL MODEL AND STUDY PARTICIPANT DETAILS

FFPE samples of 47 undifferentiated soft tissue sarcomas (USARC) (Age range 36–87 years, Male:Female 51%:49%), 53 high-grade osteosarcomas (HGOS) (Demographic data available for all but one case; Age range 5–85 years, Male:Female 44%:56%), 12 low-grade osteosarcomas (LGOS) (Age range 9–83 years, Male:Female 42%:58%), and 15 translocation-associated sarcomas (demographic data unavailable), including four synovial sarcomas (SS), nine low grade fibromyxoid sarcomas (LGFMS), and two solitary fibrous tumors (SFT), were selected for analysis based on review of haematoxylin and eosin (H&E) glass slides (and for all of which WGS data were also available). Cases were considered suitable for analysis if there was >20% viable tumor tissue on histology. FFPE blocks of tumor were obtained for resection specimens to ensure sufficient cells for FC analysis could be obtained.

Where samples had undergone decalcification, only samples decalcified using the chelating agent, ethylenediaminetetraacetic acid (EDTA), were considered suitable for analysis; decalcification with lower pH such as formic acid or nitric acid leads to depurination and DNA damage such that 4′,6-diamidino-2-phenylindole (DAPI) binding is markedly reduced.

Non-neoplastic diploid FFPE human tissues were selected to act as external control samples for FC. These samples underwent FC before and after a set of tumor samples were run, and their purpose was to identify the approximate fluorescence which diploid cells within the tumor samples would exhibit. FFPE blocks of non-neoplastic human tissue, including testis, kidney or colon were used as external controls following confirmation of normal histology under light microscopy. In tumor samples, diploid cell constituents such as lymphocytes and macrophages acted as internal diploid controls.

Ethics approval

The Royal National Orthopedic Hospital (RNOH) Biobank is approved by the National Research Ethics Committee of the Health Research Committee (reference: Integrated Research Application System (IRAS) project identifier: 272816). This study was approved by the National Research Ethics Committee approved UCL/UCLH Biobank Ethics Committee (project no: EC17.14).

METHOD DETAILS

Buffers and solutions

FACS buffer: Dissolve BSA in PBS to create a 1% solution – gently agitate the solution until all BSA granules are dissolved. Add EDTA solution (0.5M) for a final concentration of 2mM. Store at 4°C for up to 48 h (or longer if sterile filtered).

Protease solution: Dissolve 500μg of protease per mL of PBS. Gently agitate solution until all the granules are dissolved. Use immediately after creation.

Experimental ploidy measurement by FC and IC in FFPE tissues

Tissue cutting

The tumor blocks were cut using a rotary microtome. First, a 4 μm section was cut and an H&E slide was created to ensure the tissue was representative of the tumor. Then two 50 μm tissue curls were cut and placed in separate 1.5 mL Eppendorf tubes. Finally, an additional 4 μm section was cut and an H&E slide was created to ensure that the tumor tissue had not cut out or substantially changed in morphology during sectioning.

Tissue dissociation

Samples were dissociated in batches of either 8 or 16 including diploid controls. One tissue curl was transferred to a 15 mL Falcon tube. To deparaffinise the tissue, 5 mL xylene was added to each tube, samples were shaken vigorously for approximately 20 s and vortexed, then incubated for 10 min at room temperature. The sample was centrifuged at 4,400 RPM for one minute, after which the supernatant was removed. The deparaffination step was repeated once more.

Samples were then washed with alcohol and rehydrated. To do this, 5 mL of >98% industrial denatured alcohol (IDA) was added to each sample, vortexed, and incubated for 3 min at room temperature. The sample was centrifuged at 4,400 RPM for one minute and the supernatant was removed. The wash step was repeated at decreasing concentrations of IDA in distilled water (80%, 67%, 50%). Samples were then washed with distilled water, followed by phosphate buffered saline (PBS).

3 mL of protease solution (500 μg/mL) was added to each sample, then incubated at room temperature for two hours under continuous, vigorous agitation using a magnetic stir bar. It is important that the stir bar operates at a high rotational speed and that the tissue fragments are constantly agitated as insufficient tissue agitation may result in a significant reduction in the release of nuclei. The reaction was quenched by adding 3 mL of cold phosphate-buffered saline (PBS) (4°C). Samples were filtered using 40–70 μm cell strainers and then were centrifuged at 2,400 RPM for 5 min. The supernatant was removed, and the tissue was resuspended in fluorescence-activated cell sorting (FACS) buffer. The nuclei were stored for up to 24 h at 4°C prior to staining and FC.

Standardisation of nuclei density

Nuclei were counted using conventional glass haemocytometry. For each batch of samples, nuclei density (nuclei/mL) was standardised to that of the sample with the lowest nuclei density (which exceeded 1,000,000 nuclei/mL) by dilution with FACS buffer. Samples were diluted to a final volume of 500 μL. An ideal nuclei density for use in FC was 1,000,000–4,000,000 nuclei/mL. Because samples with higher densities have proportionally more debris, ensuring equivalent DAPI staining between samples becomes

challenging. Samples with low nuclei density run slowly on the flow cytometer, and this increases the relative incubation time between samples run at the start and samples run at the end of the experiment.

Staining of nuclei

Single nuclei samples were stained with DAPI at a final concentration of 1 μg/mL and incubated at room temperature for 60 min in the dark. Samples can be stained for between 30 and 60 min if the staining time is consistent within each batch. Prolonged staining time can increase the mean fluorescence intensity (MFI) of the sample.

Flow cytometry

Samples were run on the BD Fortessa ×20 flow cytometer. DAPI was excited by violet laser (405 nm), and emissions were captured with a 450/50 nm bandpass filter. Events were gated using the following gating-strategy: the first population of nuclei were identified on a density dot plot by plotting DAPI.area fluorescence vs. DAPI.width fluorescence, and objects below threshold were excluded as debris/necrosis. Importantly, this first population was not necessarily diploid but may have represented hypodiploid tumor populations – this was clarified by comparing the fluorescence intensity of the internal diploid peak with that of the external control. Doublet nuclei were then excluded by plotting forward scatter area (FSC.area) vs. FSC.height and side scatter area (SSC.area) vs. SSC.width or SSC.height (Figure S17). Voltage was determined by plotting a histogram of DAPI.area and centring the presumed diploid peak of the first sample at approximately 50,000 AU – this allowed capture of putative polyploid populations.

Samples were transferred to FC test tubes and pipette-mixed thoroughly using a P1000 pipette. Samples were run in batches of up to eight to minimise variation in incubation time between the first sample run and the last.

An external control sample was run immediately prior to the tumor samples so that the diploid peaks of the tumor samples could be reliably identified (Figure S18). The external control sample was also run immediately following the tumor samples to control for any additional incubation time caused by waiting for the other tumor samples to be processed. Where possible, 20,000 gated events were captured for each sample.

FC data was overall of high quality, with mean CV of the diploid peak across all samples of 4.79% ± 1.48% (StDev).[47,48]

DNA ploidy analysis

FCS files obtained from FC were analyzed by Floreada.io SIMD, and FCS Express 7 Research Edition. Each sample was gated as per the gating strategy described above, then DNA content (ploidy) was calculated. To identify each population of nuclei in the sample, a histogram was plotted using DAPI.area, and for delineation of nearby populations, a further plot of DAPI.area vs. side-scatter.height or side-scatter.width was examined. Distinct peaks on the histogram were identified by eye as discrete populations and comprised ≥6% of the total gated events. For peaks which lay within a tetraploid DNA range (3.8*N*–4.2N), they were considered aneuploid tumor populations rather than just G2/M populations if the population size was ≥15% of the total gated events, or if there was an identifiable corresponding S-phase or G2/M peak, consistent with previous consensus literature.[49] With respect to peak definition, gates were applied manually at the points where the histogram returned to baseline on either side of each distribution. Additionally, tumor ploidy assignment was supported by cross-comparison of flow cytometry–derived purity estimates with histological assessment and CNA-based estimates. In cases of discrepancy, results were re-evaluated. For example, a tumor inferred as diploid by flow cytometry but appearing tetraploid with low purity by sequencing may reflect either an undetected minor WGD population or misfitting of the sequencing data, as described by Tarabichi et al. In such cases, histological estimates of tumor purity, while not perfectly precise, typically fall within a 10–20% margin of error and can help resolve the discrepancy.[50]

For robust calculation of the coefficient of variation (CV), samples were analyzed using multicycle modeling with FCS Express 7 Research Edition. For DNA content analysis and population size, each peak was manually gated in Floreada.io SIMD after doublet discrimination, and the MFI was calculated. The internal diploid control (comprising constituent diploid cells with ploidy 2N) was identified by comparing the MFI of the peak with that of the external diploid control sample.

The ploidy for each tumor population within the sample was calculated as:

$$Relative\ Ploidy = \frac{MFI_{tumour}}{MFI_{diploid}} \times 2$$

where:

MFI*tumour* is the mean fluorescence intensity of the tumor sample.

MFI*diploid* is the mean fluorescence intensity of a diploid reference sample.

In-silico flow cytometry validation

Flow cytometry standard (FCS) files were obtained from two tumor samples and one diploid control. Tumour-associated events, including a broad range of debris, were extracted using Floreada.io to minimise bias from restrictive gating. Based on prior flow cytometry analysis, USARC-4 contained a single tumor population with a ploidy of 3.4N, while USARC-33 contained two tumor populations with ploidies of 2.6N and 5.1N respectively.

Flow cytometry event data were exported as CSV files and analyzed in R (v4.4.1), with packages dplyr (v1.2.1) and ggplot2 (v4.0.2). For each dataset, the DNA content channel (DAPI-A) was extracted, converted to numeric format, and filtered to retain finite, positive values only.

Synthetic populations were generated *in-silico* by randomly sampling events and combining them at defined proportions across a range of purities (defined here as purity sweeps). Mixture series included: tumor purity values (10–90%), equal tumor mixtures with

≥10% diploid, skewed tumor mixtures with fixed diploid proportion, haploid spike-in series generated by halving tumor DNA values (yielding an effective ploidy of approximately 1.7N). Simple kernel density estimation was used to detect peaks using base R functions.

Image cytometry

FFPE blocks were sectioned into 50μm scrolls, the procedure used was a modification of Hedley et al.[27] After being dewaxed in xylene and rehydrated progressively in ethanol and water, the sections were digested for two hours at room temperature using proteinase XXIV (Sigma-Aldrich, Dorset, UK) at a concentration of 0.5 mg/mL.

Following digestion, the samples were put on ice and filtered through 40 μm mesh (BD Biosciences, California, USA). The samples were then centrifuged, the pellets of nuclei were resuspended in PBS according to the pellet size. Appropriate number of nuclear suspensions were pipetted into a Shandon single-use EZ-Cytofunnel (Thermo Scientific, Basingstoke, UK) and spun at 225 × g for five minutes using a Shandon Cytospin 2 onto a Superfrost Plus microscope slide (Thermo Scientific).

The monolayers were left to dry at room temperature for at least 1 h, before being fixed in 10% formalin overnight. The following day, the monolayers had their DNA hydrolyzed in 5M HCl for one hour, then treated with Schiff's reagent for two hours.

Using the automated image cytometry analyzer Fairfield DNA Ploidy system (Fairfield Imaging, Kent, UK), which is made up of a high-resolution digital camera (model C4742-95, Hamamatsu Photonics, Japan), a 546-nm green filter, and a Zeiss Axioplan microscope (Zeiss, Jena, Germany).

For every nucleus, the integrated optical density (IOD) was calculated. Every cell type was represented by four galleries: lymphocytes, plasma cells, fibroblasts, and nuclei of relevance for measurement. A minimum of 2000 nuclei were automatically grabbed and sorted using a rule file. The lymphocytes served as the diploid peak's internal control (2c).

The galleries were then manually edited, any sliced or overlapping nuclei missed by the rule file were discarded. The measurement of integrated optical density produces a histogram of DNA content.

A specimen was defined as diploid when there was only one peak (which was 2c, or DI (0.9–1.1). A specimen was defined as tetraploid when there was a population of 4C nuclei (DI (1.9–2.1). A specimen was defined as tetraploid when the percentage of 4C fraction exceeds 10% of the total number.

For comparison between IC and FC, data from IC param files was extracted using Python3 code. The IOD of single nuclei were then plotted on a DNA content histogram using R-Studio and Shiny Server. For the purposes of consistency and comparability, these DNA content histograms were analyzed using the same population rules as applied to the FC data.

WGS protocol and data processing

Whole genome sequencing data analysis

For the USARC dataset, WGS was performed on frozen tumor samples with the Xten instrument (Illumina, Chesterford, UK). The average coverage of tumor DNA was at least 70× and at least 30× for normal DNA. The fastq files were aligned to human genome version GRCh37 using BWA mem as described in Steele et al.[12]

For the osteosarcoma (OS) dataset, genomic data were prepared as described by Valle-Inclan et al.[20]

For the translocation-associated sarcoma datasets, genomic data were[29] analyzed as described by Prendergast et al.[46]

Copy number analysis

We obtained ploidy estimates and copy-number profiles from WGS data using three widely used algorithms: Battenberg,[7] PURPLE (3.8),[43] and FACETS (0.6.2).[17]

For the OS datasets, FACETS and Battenberg were run using default settings. PURPLE copy number data for the OS dataset were obtained from published data as described.[20]

For the USARC dataset FACETS was benchmarked on five representative samples. Using default settings, FACETS was unable to model ploidy values for all five samples, therefore after optimisation, FACETS was run on the USARC dataset using cval 1500, SNP neighborhood size 1000, and min.nhet 9. For Battenberg, the default settings were used. For PURPLE, the prerequisite softwares AMBER and COBALT were run to obtain B-allele frequency (BAF) and read-depth ratios for tumor samples, after which PURPLE was run using default settings and only mandatory arguments.

Manual refitting of CNA profiles

Manual refitting of CNA profiles was performed by two experts (MT and MF) in copy number analysis. USARC cases were refitted blind to experimental ploidy measurements, based on SNV clustering, the presence of half-integer copy-number states, the fraction and size of homozygous deletions, and the fraction of loss of heterozygosity.

Correction of ploidy and WGD estimates

The WGS-derived ploidy and purity estimates were derived using the previously described Battenberg algorithm.[7,28] WGS-derived ploidies were compared to those from FC. Samples were considered 'discrepant' if the FC ploidy of the largest tumor population differed by ≥ 1N from the WGS ploidy or if the difference affected WGD status as defined below. For tumors with multiregional sampling, the most frequently occurring ploidy solution across regions was taken as the tumour-wide consensus. For each discrepant sample, a ploidy range approximately ±1.0N from the FC estimate and excluding the original Battenberg ploidy was provided to the CNA caller for ploidy re-estimation. For example, if Battenberg originally selected 2.0N and FC estimated 4.0N, the new range would be 3.0*N*–5.0N. Where the discrepancy between the WGS-derived ploidy and the FC-derived was smaller than 1N, the acceptable ploidy range was reduced to approximately 0.5N above and below the FC ploidy estimate. Giving a range rather than an exact

ploidy value theoretically allows the algorithm to account for small variations in tumor composition between the tumor section used for WGS and the section used for FC and other small biological variations.

WGD was identified by plotting FC-derived ploidy against the proportion of the genome with a loss of heterozygosity (propLoH) as estimated by Battenberg. Tumors were defined as WGD using the following formula as described previously:

$$Ploidy > -2 \times propLoH + 2.9$$

GISTIC analysis of gene amplification and deletion profiles

To identify significant amplifications and deletions within tumor datasets, we employed the Genomic Identification of Significant Targets in Cancer (GISTIC) version 2.0.3. GISTIC analysis was performed using segmented LogR data generated by Battenberg analysis of WGS data. Allele-specific copy numbers (nMaj and nMin) from the clonal tumor fraction were summed to compute total copy number per segment. GISTIC-compatible log2 ratios were then calculated using the formula log2[(nMaj + nMin)/2], where a diploid genome corresponds to a log2 ratio of 0. Homozygous deletions (nMaj+nMin = 0) were assigned a value of −2 to denote deep deletions. Gene-level analysis was performed (-genegistic 1). Both broad and focal copy-number changes were assessed. Broad regions were defined as alterations involving ≥98% of the chromosome arm (-brlen 0.98), while focal alterations were identified using the ‘extreme’ genomic coverage model (-gcm extreme). Arm-level peel-off was enabled (-armpeel 1). The deletion and amplification thresholds were set to 0.25 (-ta 0.25 -td 0.25) The significance level was set to a relatively high threshold of 0.9 (default 0.75). The sample center (-scent) option was set to default (median). All other GISTIC parameters were set to default settings.

DPClust subclonal SNV clustering

DPClust v2.2.8[44] was run on tumor samples using default metrics. Consensus somatic SNV files were generated based on legacy data[45] from SNV callers Strelka2[51] and Mutect2.[52] An SNV was included in the dataset if it was present in both callers.

Cell segmentation

We used CellViT[53] to perform nucleus segmentation on 14 WSI. Following segmentation, nucleus area was calculated using the python package numpy to extract nuclear area. Following this, a large area of viable tissue was identified on the WSI from which five random boxes measuring 100,000 squared pixels were selected. The mean area in pixels squared was calculated from all nuclei within the five boxes.

QUANTIFICATION AND STATISTICAL ANALYSIS

Unless otherwise specified, statistical analyses were performed using R (v4). To assess the concordance between CNA caller ploidy estimation, the concordance between FC and IC, and the concordance of Battenberg ploidy and FC-derived ploidy, Pearson correlation coefficients were calculated. To assess the agreement between the two manual CNA refits, intraclass correlation coefficient ICC(3,k) was calculated. Recurrent copy-number amplifications and deletions were identified using GISTIC2.0 with the thresholds described above. Unless otherwise specified, statistical significance was defined as $p < 0.05$.

Cell Reports Methods, Volume *6*

Supplemental information

Improving computational tumor ploidy estimation in complex cancer genomes through flow-cytometry-guided calibration

Thomas Butters, Dahmane Oukrif, Punn Tannirandorn, Ianthe AEM van Belzen, Jianan Chen, Christopher Davies, Runtian Lin, Solange De Noon, Dalil Taher, Chloe Cutler, Mark Markov, Anna Tollit, Fatine Oumlil, Christopher D. Steele, Matthew Fittall, Yanping Guo, Isidro Cortés-Ciriano, Peter Van Loo, Adrienne M. Flanagan, Nischalan Pillay, and Maxime Tarabichi

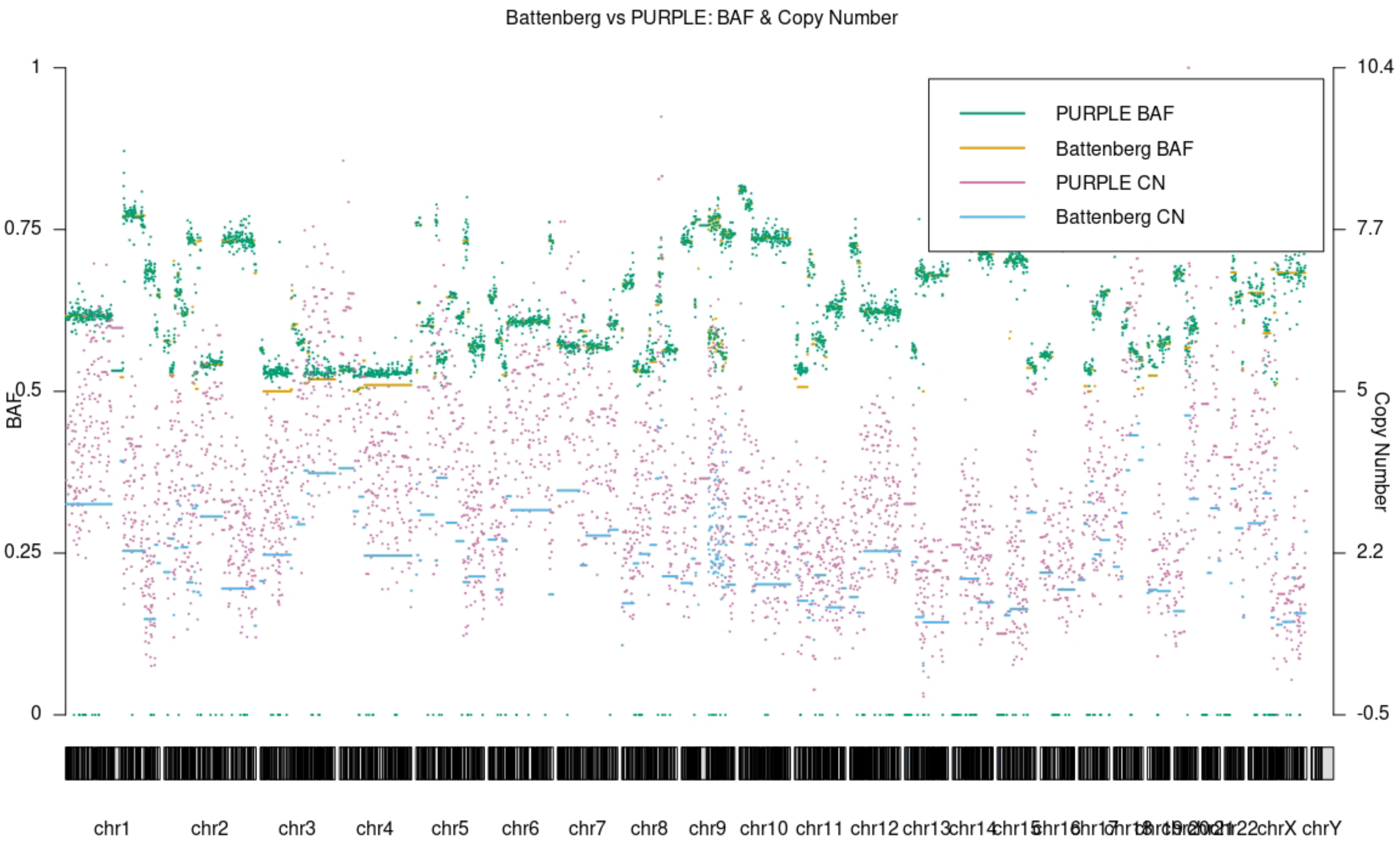

Supplementary Figure 1. Comparison of mirrored BAF and copy number between Battenberg and PURPLE on USARC-35, related to Figure 1.
The BAF are similar between the two CNA callers, however, due to the hyper-segmentation of the genome by PURPLE, the copy number is less concordant.

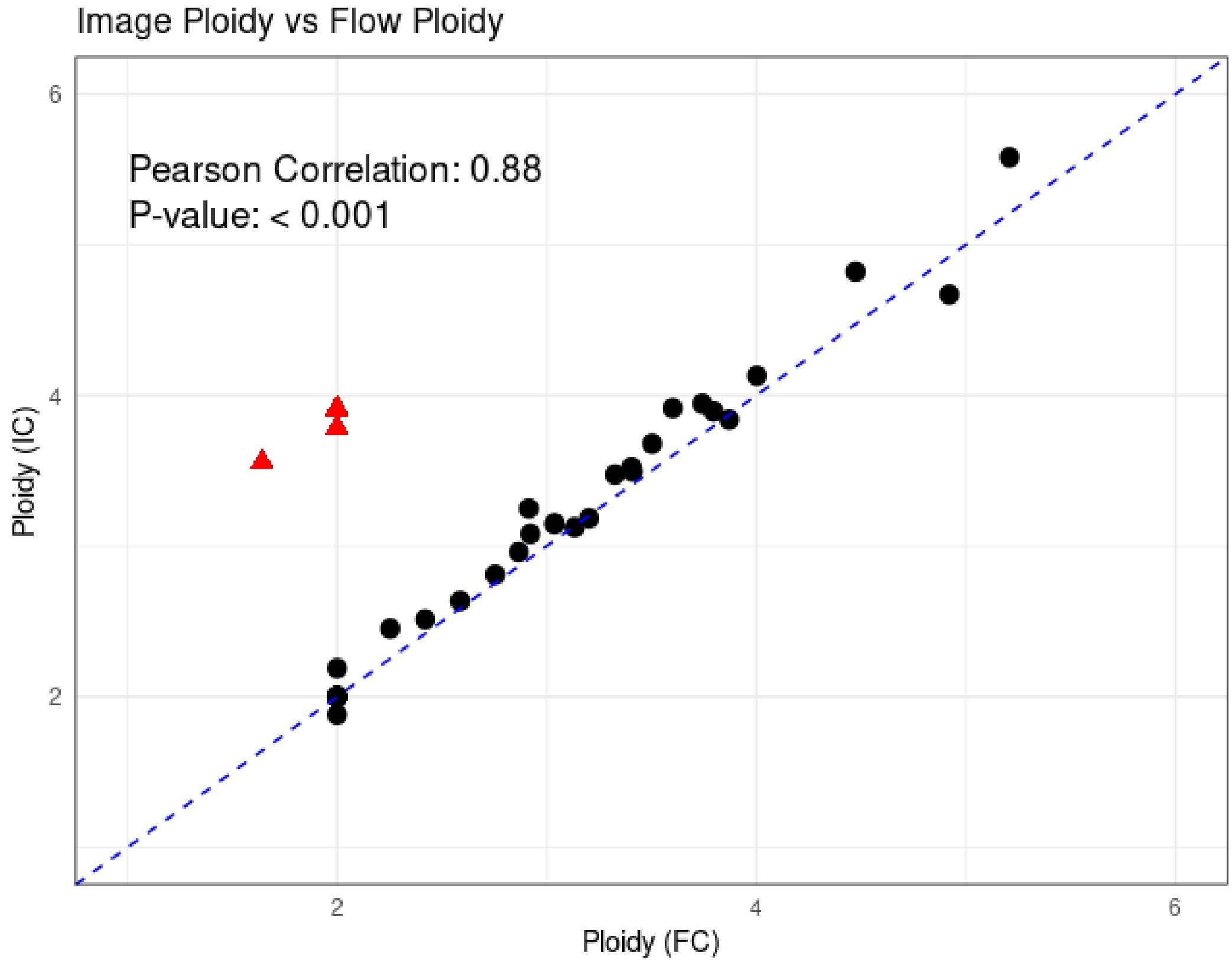

Supplementary Figure 2. Correlation between ploidy estimates derived by image cytometry (IC) and flow cytometry (FC), related to Figure 2.
Linear regression showing the correlation of ploidy estimates derived by IC vs FC of the primary tumour populations of USARC and OS samples. There is a very strong

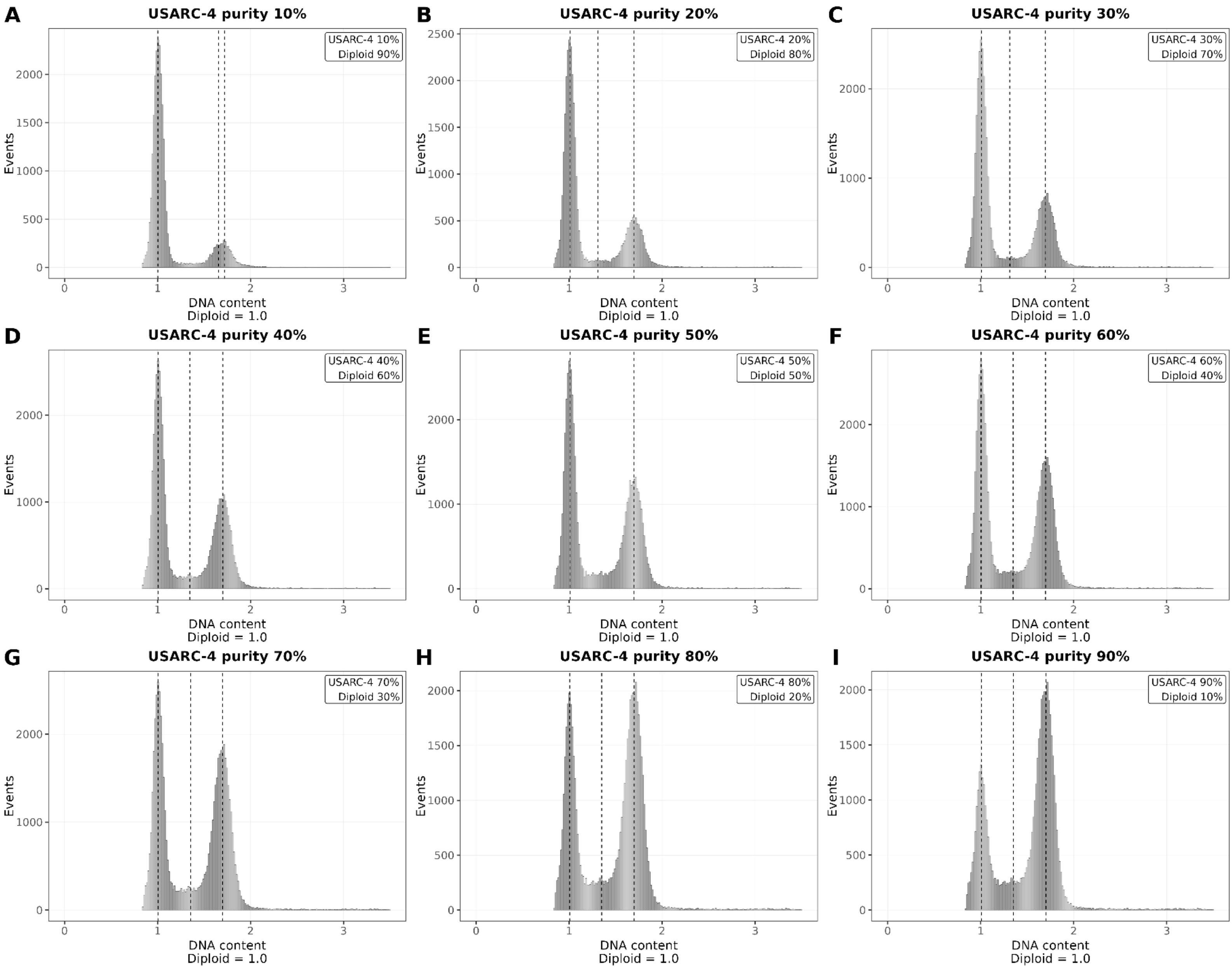

Supplementary Figure 3. Simulated USARC-4 tumour purity sweep, related to Figure 2.
Histograms show normalised DNA-content distributions generated by mixing USARC-4 tumour events with diploid control events at increasing tumour fractions from 10% to 90% (A–I). DNA content was normalised such that the diploid peak is centred at 1.0. Dashed vertical lines indicate density-detected peaks, and inset labels show the tumour and diploid proportions used for each simulated mixture.

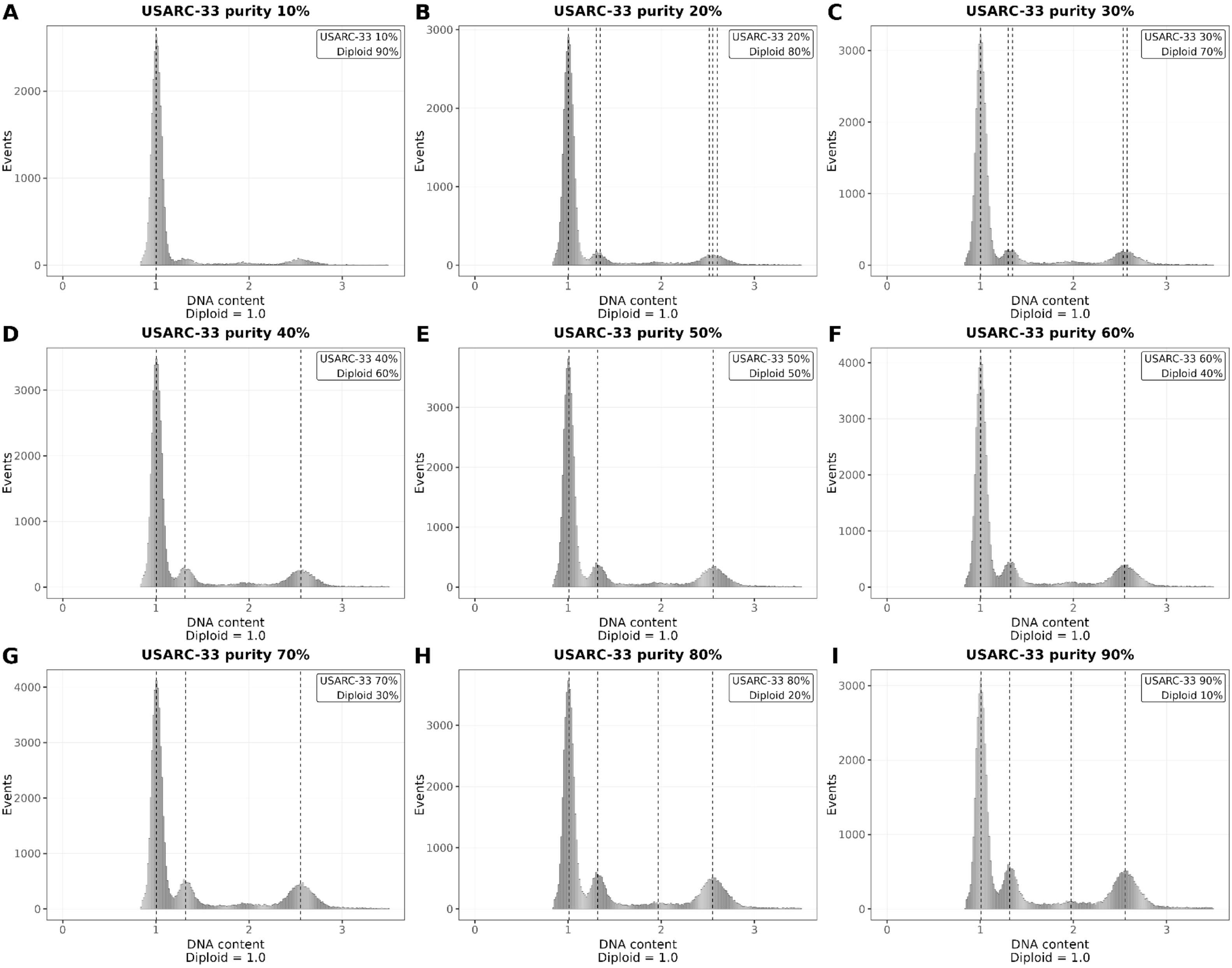

Supplementary Figure 4. Simulated USARC-33 tumour purity sweep, related to Figure 2.
Histograms show normalised DNA-content distributions generated by mixing USARC-33 tumour events with diploid control events at increasing tumour fractions from 10% to 90% (A–I). DNA content was normalized such that the diploid peak is centred at 1.0. Dashed vertical lines indicate density-detected peaks, and inset labels show the tumour and diploid proportions used for each simulated mixture.

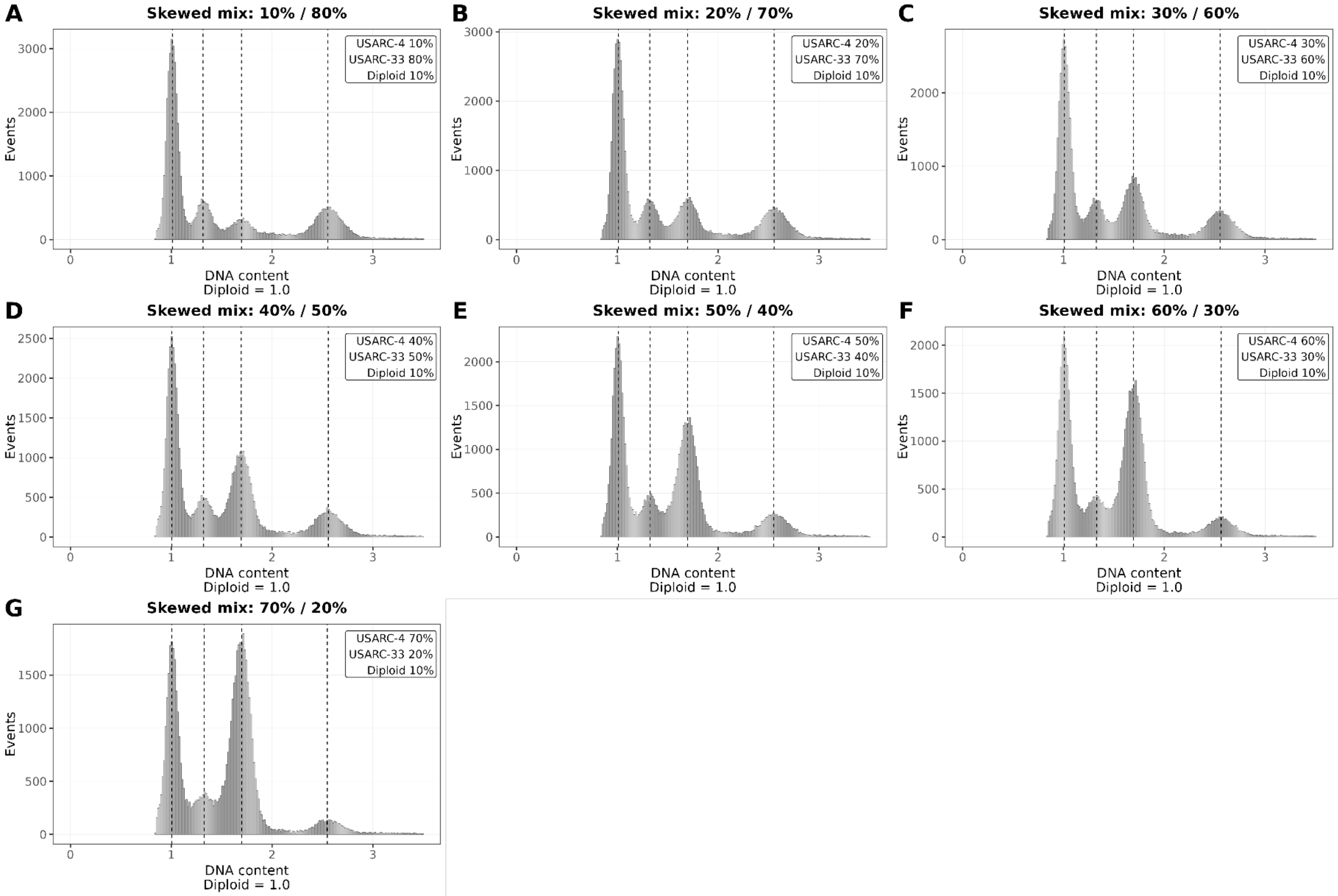

Supplementary Figure 5. Skewed simulated USARC-4 and USARC-33 tumour mixtures, related to Figure 2. Histograms show normalized DNA-content distributions generated by mixing USARC-4 and USARC-33 tumour events at reciprocal proportions while maintaining a fixed 10% diploid component (A–G). USARC-4 increases from 10% to 70%, while USARC-33 decreases from 80% to 20%. DNA content was normalized such that the diploid peak is centered at 1.0. Dashed vertical lines indicate density-detected peaks, and inset labels show the mixture proportions used for each simulated sample.

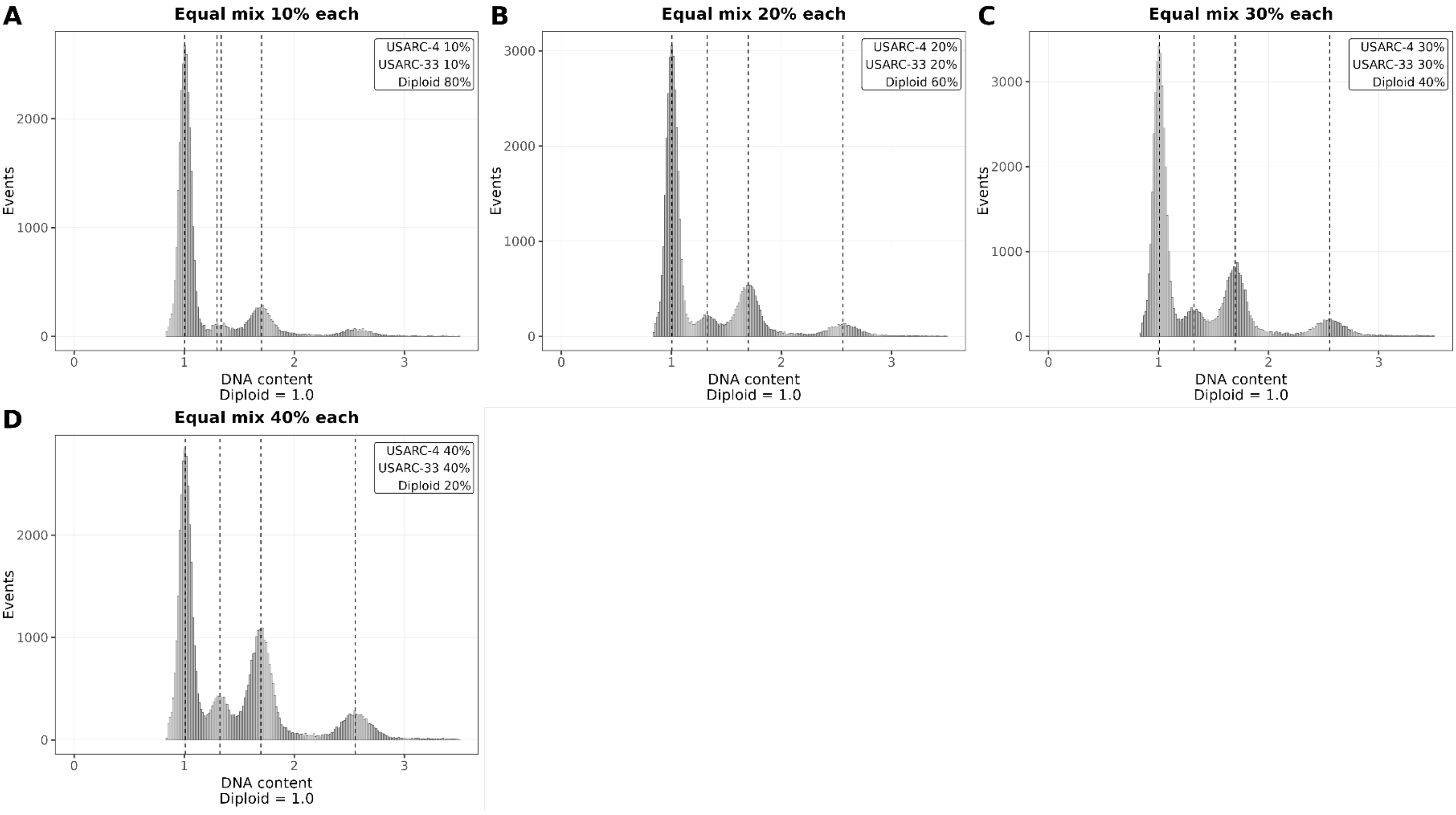

Supplementary Figure 6. Equal simulated USARC-4 and USARC-33 tumour mixtures, related to Figure 2. Histograms show normalized DNA-content distributions generated by mixing USARC-4 and USARC-33 tumour events at equal proportions, ranging from 10% each to 40% each (A–D), with the remaining events comprising the diploid component. DNA content was normalized such that the diploid peak is centered at 1.0. Dashed vertical lines indicate density-detected peaks, and inset labels show the USARC-4, USARC-33, and diploid proportions used for each simulated mixture.

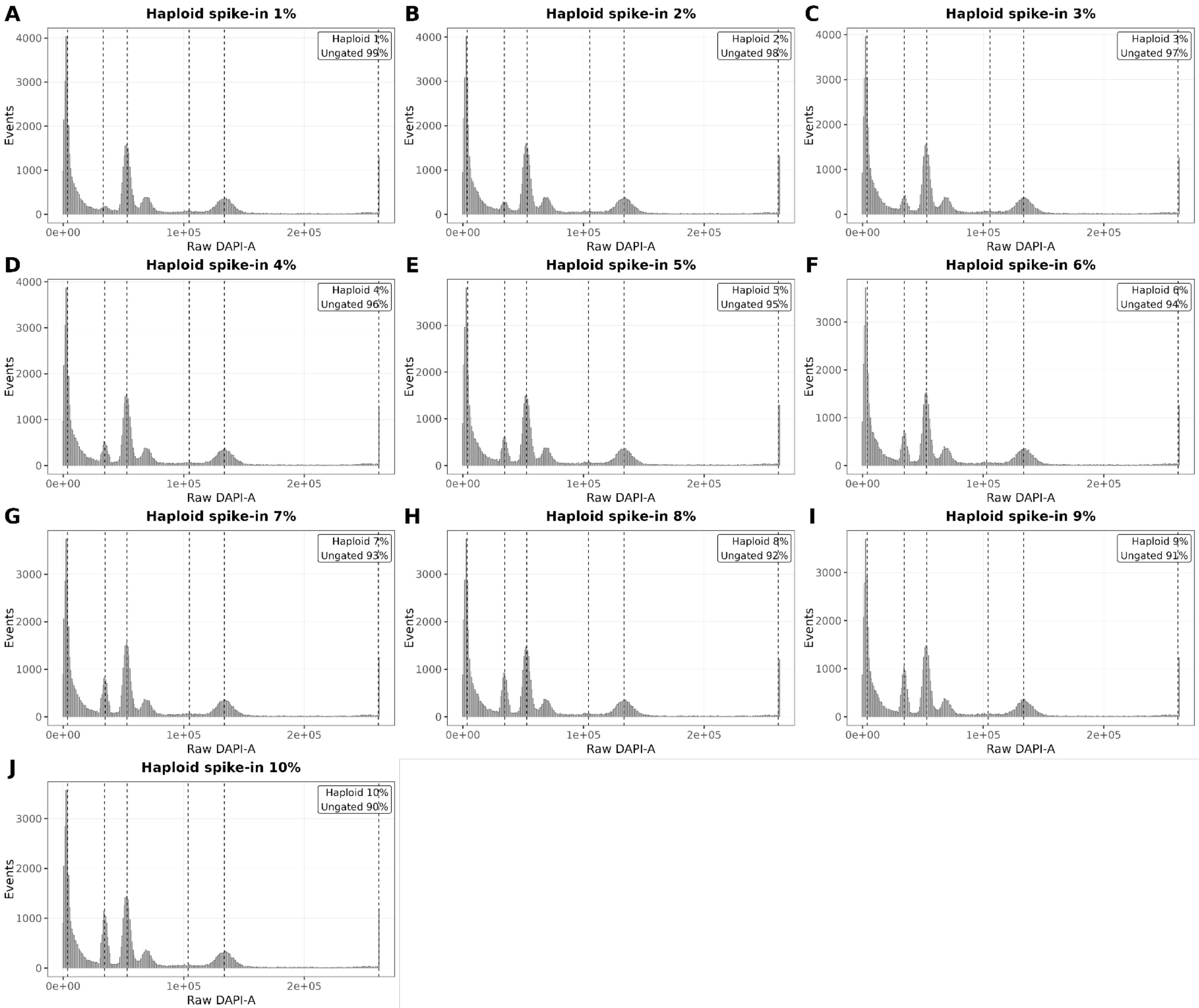

Supplementary Figure 7. Synthetic haploid spike-in sweep in ungated 3269 events, related to Figure 2. Histograms show raw DAPI-A distributions generated by adding a synthetic haploid-like population, derived by halving the first gated 3269 tumour peak, into the unmodified ungated 3269 background at spike-in fractions from 1% to 10% (A–J). Dashed vertical lines indicate density-detected peaks, and inset labels show the synthetic haploid and ungated proportions used for each mixture.

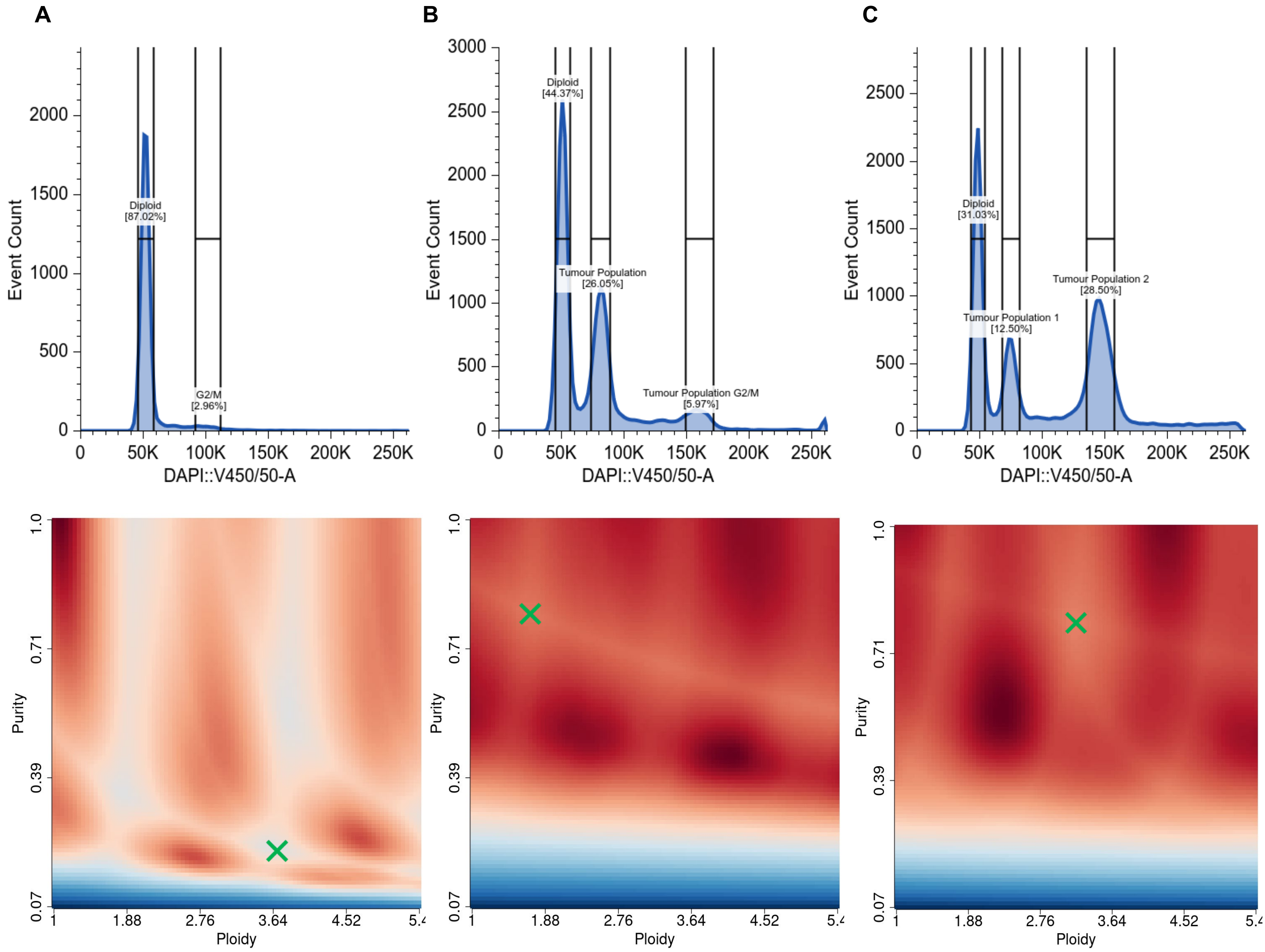

Supplementary Figure 8. Examples of discordant ploidy solutions between flow cytometry and Battenberg in USARC samples, related to Figure 2.

A) An example of a USARC sample which is near-diploid by FC (top), but has been erroneously given a ploidy of 3.68N by Battenberg on the sunrise plot (bottom) as indicated by a green cross. There is an equivalent solution at a ploidy of approximately 1.9N at a higher purity estimate. B) An example of a USARC sample which has a ploidy of 3.2N by FC (top), but has been erroneously given a ploidy of 1.92N by Battenberg on the sunrise plot (bottom). There is an equivalent solution at a ploidy of approximately 3.5N at a lower purity estimate. C) An example of a USARC sample which has multiple aneuploid tumour populations with ploidies of 3.1 and 6.0 respectively. Battenberg has chosen a ploidy solution equivalent the first aneuploid population, but not the largest tumour population. Ploidy solutions exist for this tumour at a ploidy of approximately 6.6 (not-shown).

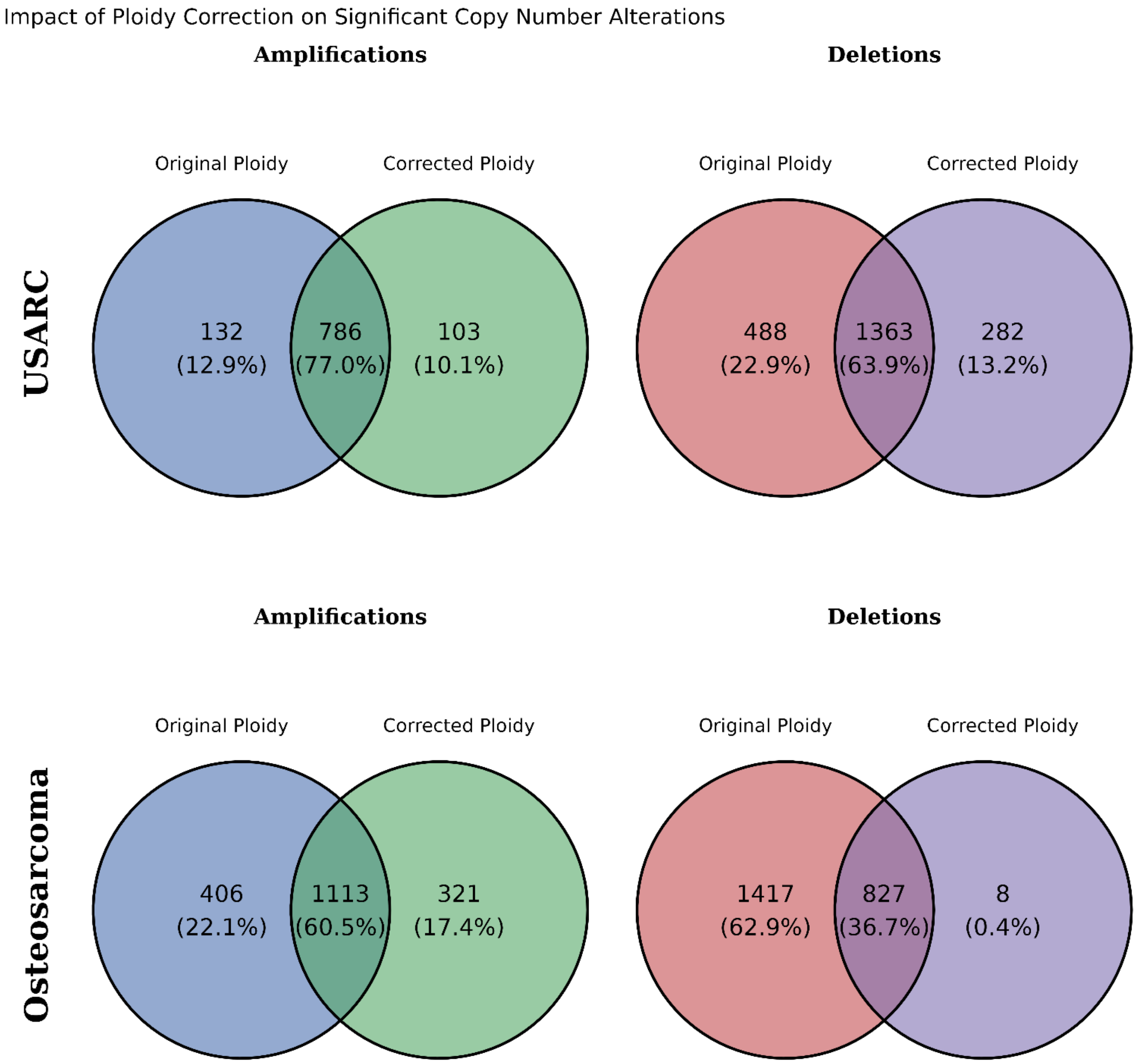

Supplementary Figure 9. Impact of ploidy correction on significant copy number alteration calls in USARC and osteosarcoma cohorts, related to Figures 3 &4.
Venn diagrams compare significant copy number alterations identified using original ploidy estimates versus ploidy-corrected estimates. Results are shown separately for amplifications and deletions in the USARC cohort (A) and the osteosarcoma cohort (B). Numbers indicate the count of significant alterations unique to the original ploidy analysis, shared between both approaches, or unique to the corrected ploidy analysis. Ploidy correction retained the majority of amplification calls in both cohorts, while a larger shift was observed for deletion calls, particularly in osteosarcoma, where most deletions were unique to the original ploidy analysis.

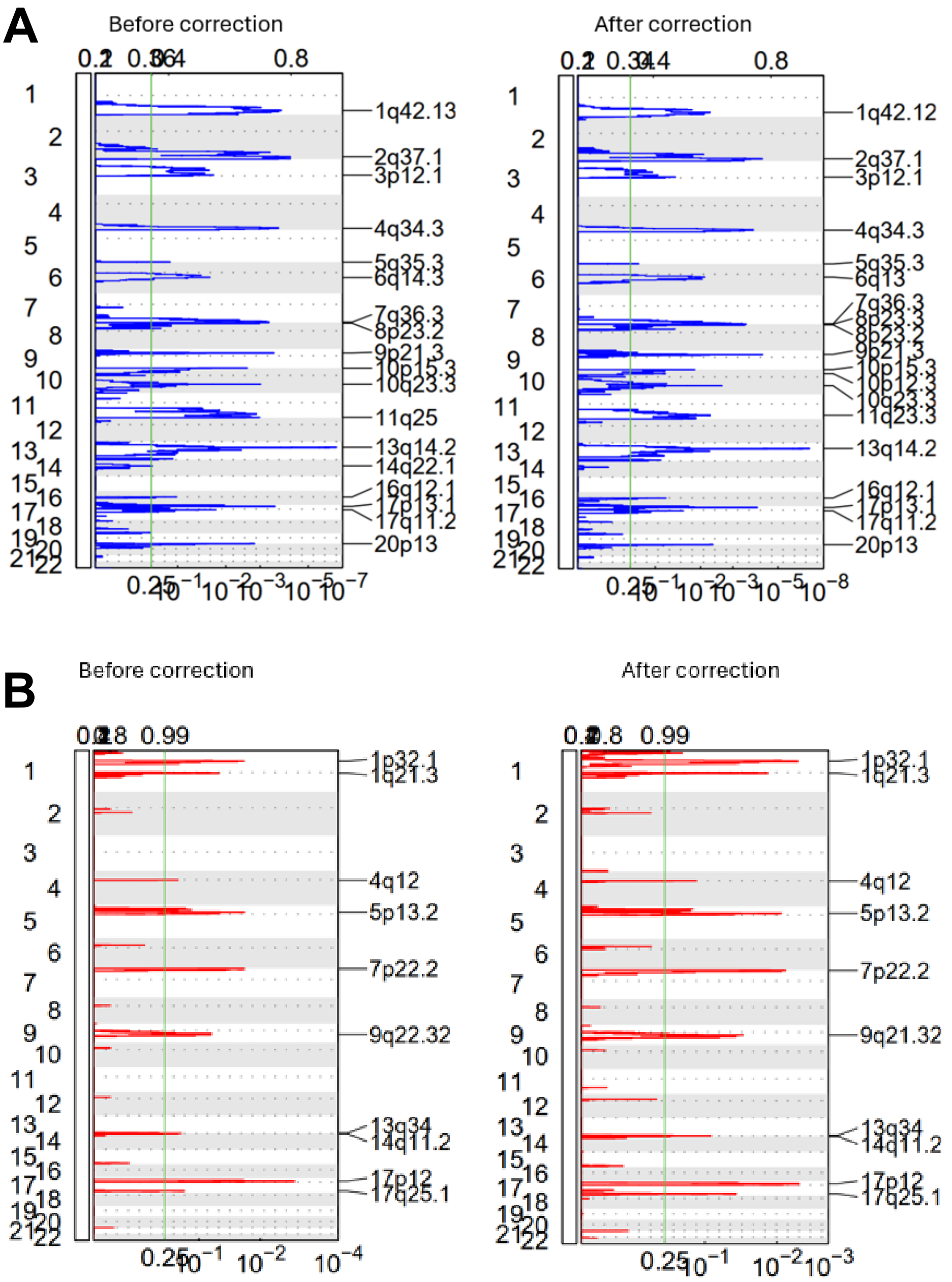

Supplementary Figure 10. Effect of ploidy correction on GISTIC copy-number calls, related to Figures 3 &4..
Genome-wide GISTIC deletion (A) and amplification (B) profiles are shown before and after ploidy correction. The x-axis shows GISTIC significance, reported as −log10(q-value), and the y-axis shows chromosomal position across autosomes 1–22. Vertical green lines indicate the significance threshold, and selected significant peaks are labelled by cytoband. Ploidy correction altered the significance of some recurrent events, with additional arm-level or focal peaks crossing the GISTIC threshold after correction.

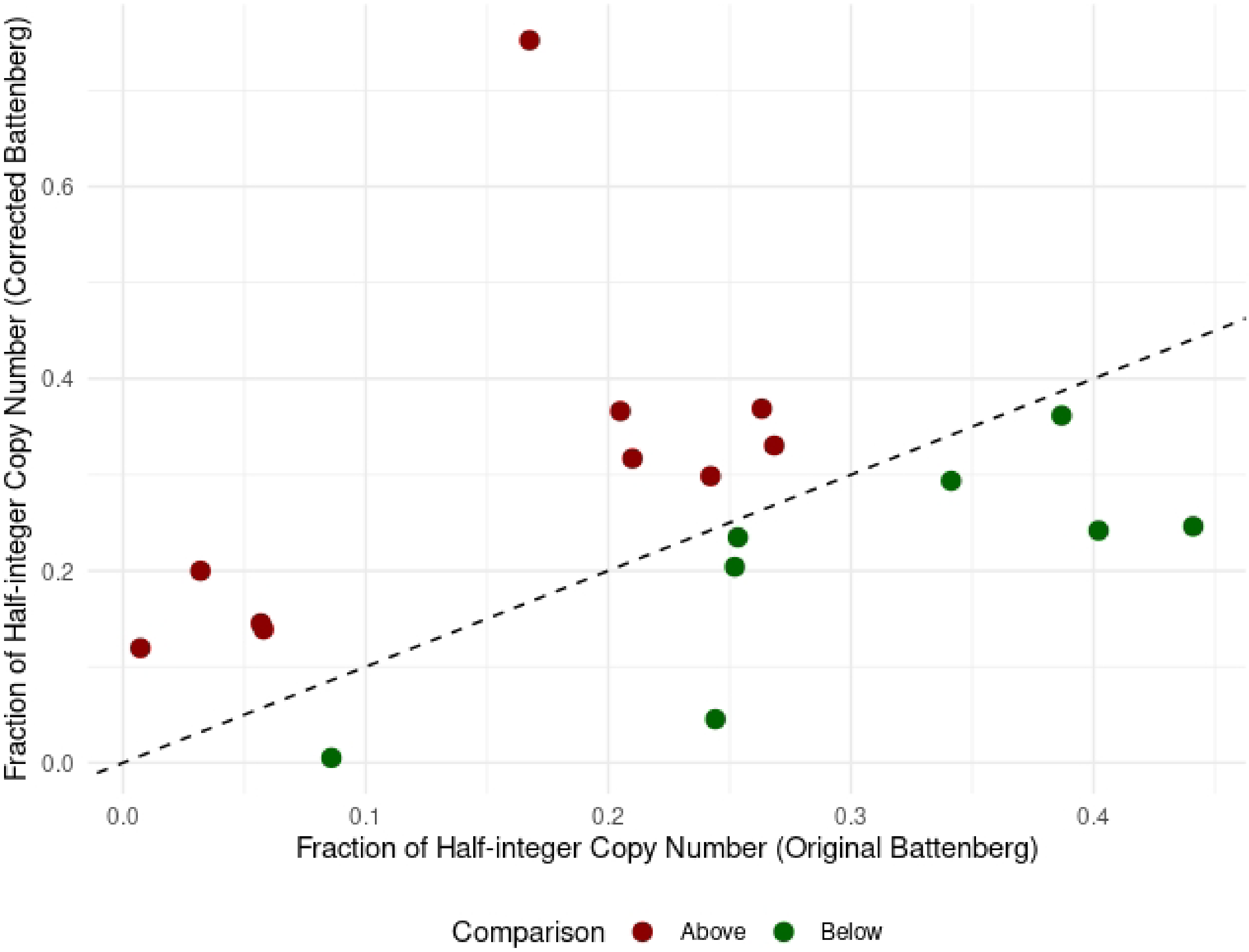

Supplementary Figure 11. Impact of ploidy correction on the proportion of half-integer copy number states, related to Figure 4.
Scatter plot comparing the proportion of each tumour genome lying at half-integer values using Battenberg copy number modelling before and after ploidy correction. Green points lying below the y = x line represent samples where the original Battenberg profiles harbour a greater proportion of half-integer values. Red points lying above the y = x line represent samples where the corrected Battenberg profiles harbour a greater proportion of half-integer values. The plot demonstrates that using the presence of half-integer values to correct ploidy in-silico, is uninformative for the USARC dataset.

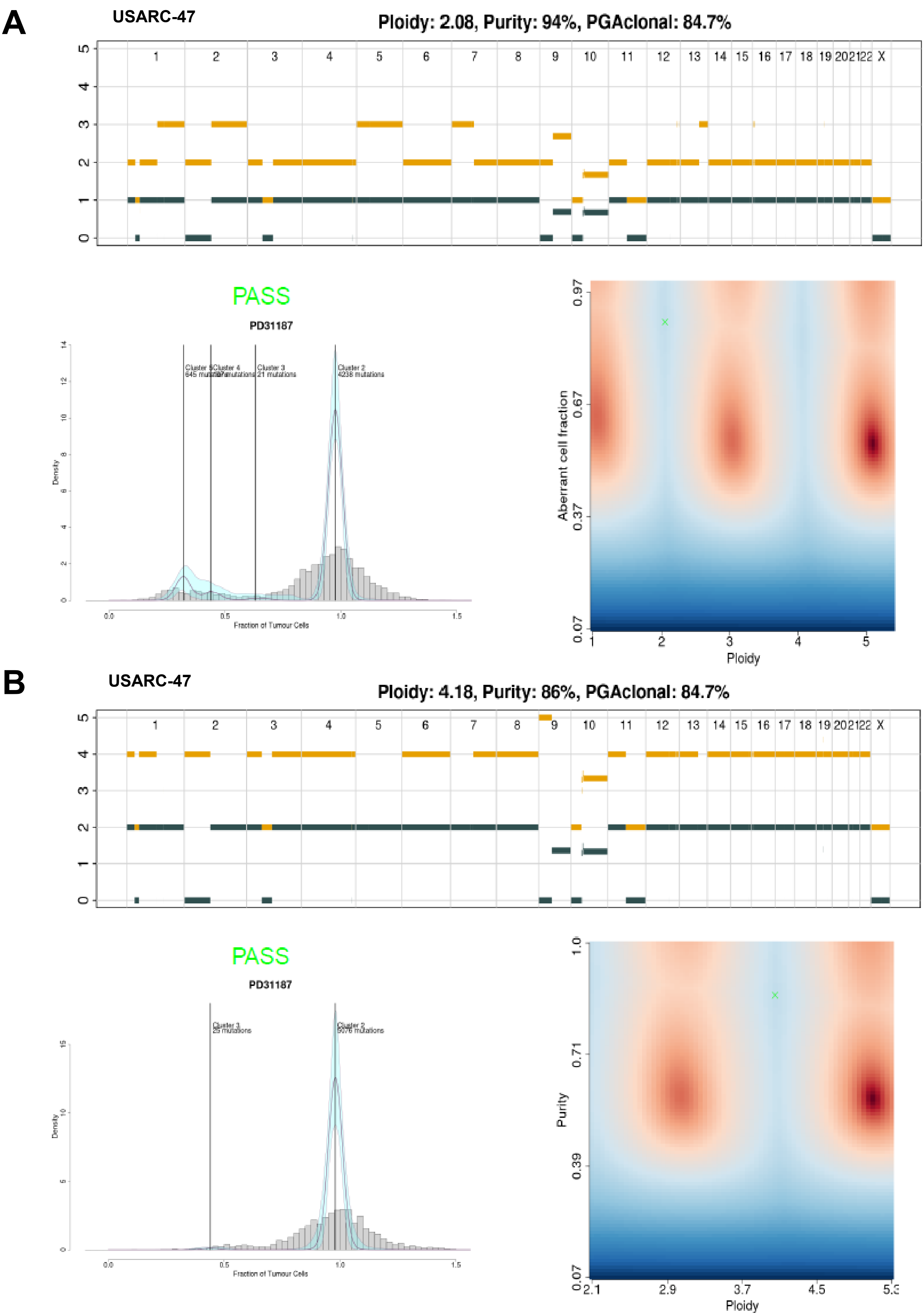

Supplementary Figure 12. SNV clustering analysis of USARC sample USARC-47 before and after flow cytometry ploidy correction, related to Figure 4..

SNV clustering analysis of USARC sample USARC-47 before (A) and after (B) ploidy correction by FC. Each figure consists of copy number profile (top), DPClust SNV clustering analysis (bottom left), and sunrise plot (bottom right). The CCF of the clonal SNV peak does not change before and after correction, and this is most likely due to the presence of multiple possible purity values for each ploidy solution (evident on sunrise plot).

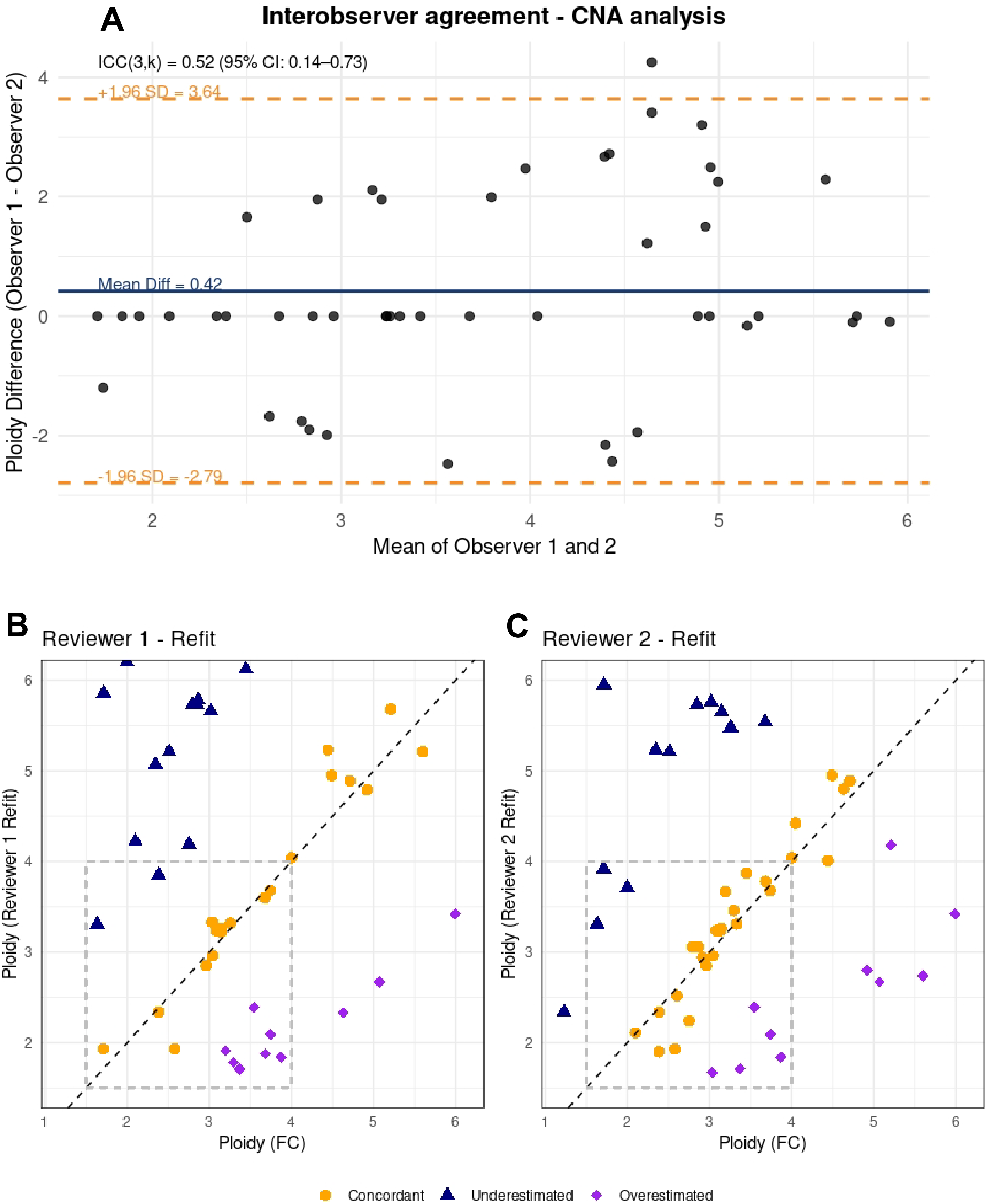

Supplementary Figure 13. Interobserver agreement and correlation with flow cytometry for refitted Battenberg ploidy estimates, related to Figure 4..

(A) Bland-Altman plot showing low-moderate interobserver agreement between refitted Battenberg ploidy estimates generated by reviewer 1 and reviewer 2. (B) Correlation between FC and refitted Battenberg ploidy estimates generated by expert 1 (B) and expert 2 (C). There is a discrepancy of 51% and 38% respectively between reviewers 1 and 2 and the FC ploidy.

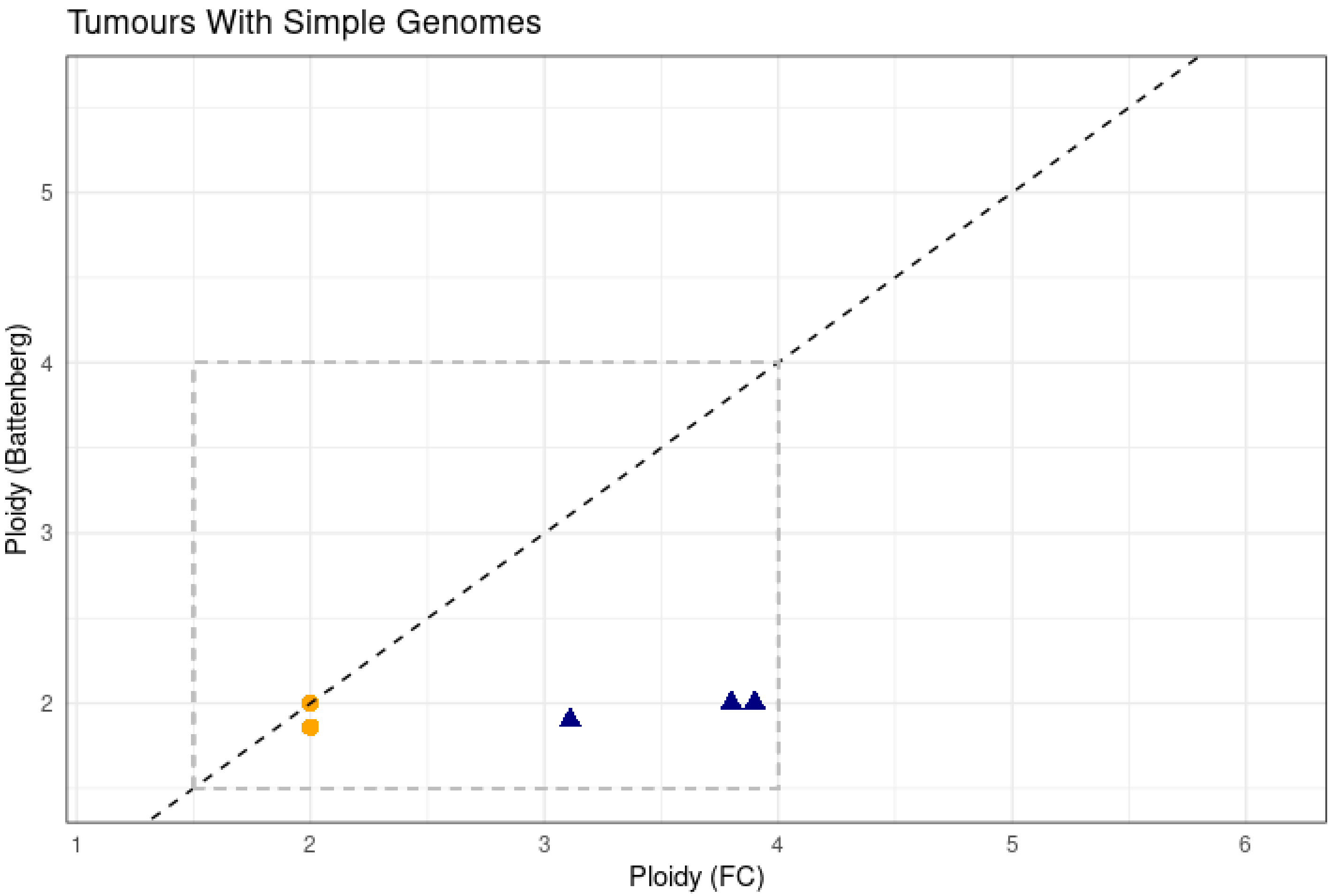

Supplementary Figure 14. Correlation between flow cytometry and Battenberg ploidy estimates in tumours with simple genomes, related to Figure 4..

The figures shows the correlation between FC-derived ploidy estimates of the primary tumour peaks, and Battenberg-derived estimates of tumour ploidy in tumours with relatively simple genomes including SS, LGFMS, SFT, and LG-OS. There is a 11% discrepancy. 3/27 samples (navy) were underestimated compared with the FC ploidy.

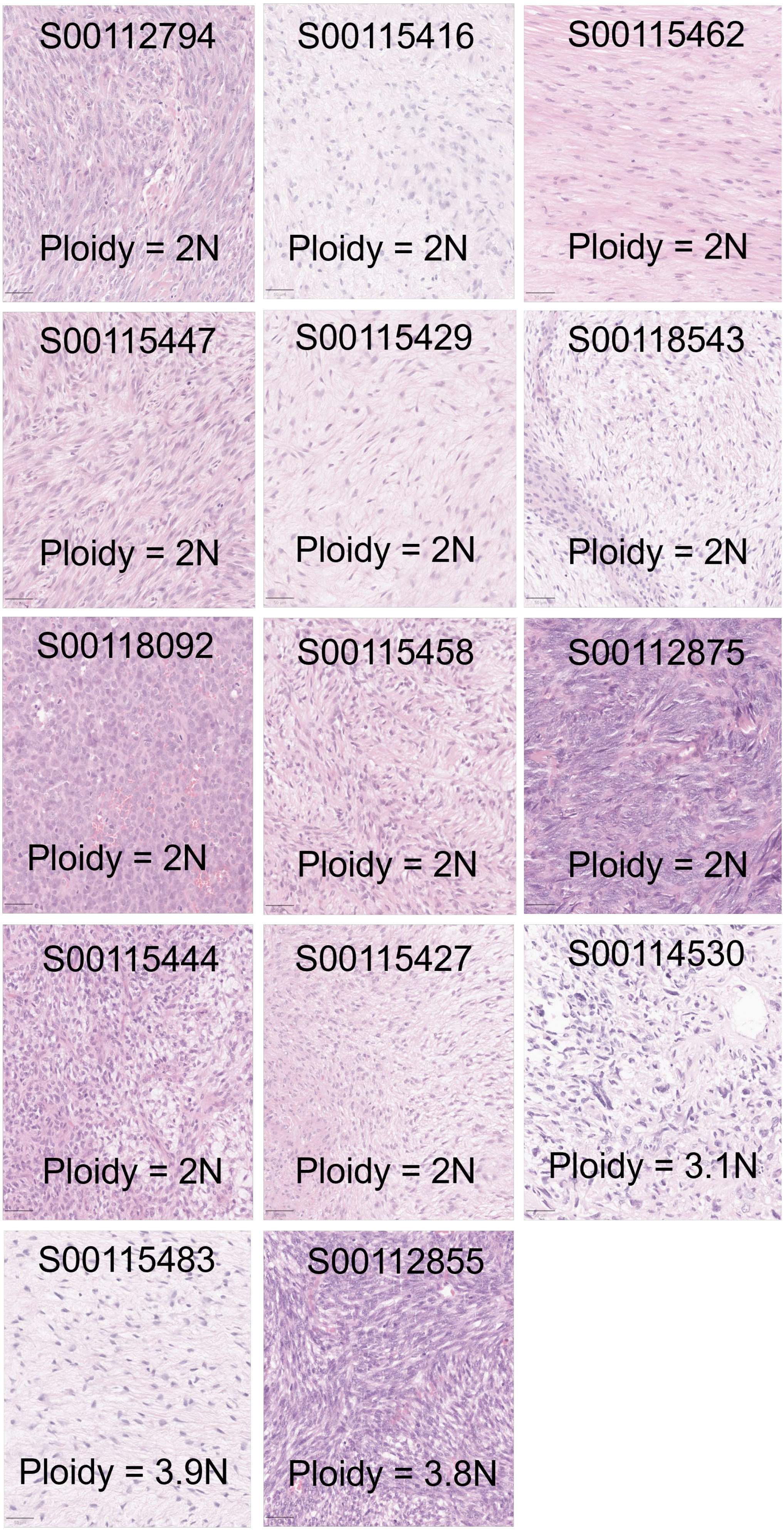

Supplementary Figure 15. Example tiles from H&E scanned whole-slide images of chromosomally simple tumours, related to Figure 4..

Example tiles from H&E-scanned WSI of chromosomally simple tumours at x40 magnification, with relevant FC-derived ploidy measurements. Scale bars in the bottom left of each tile measure 50 μm.

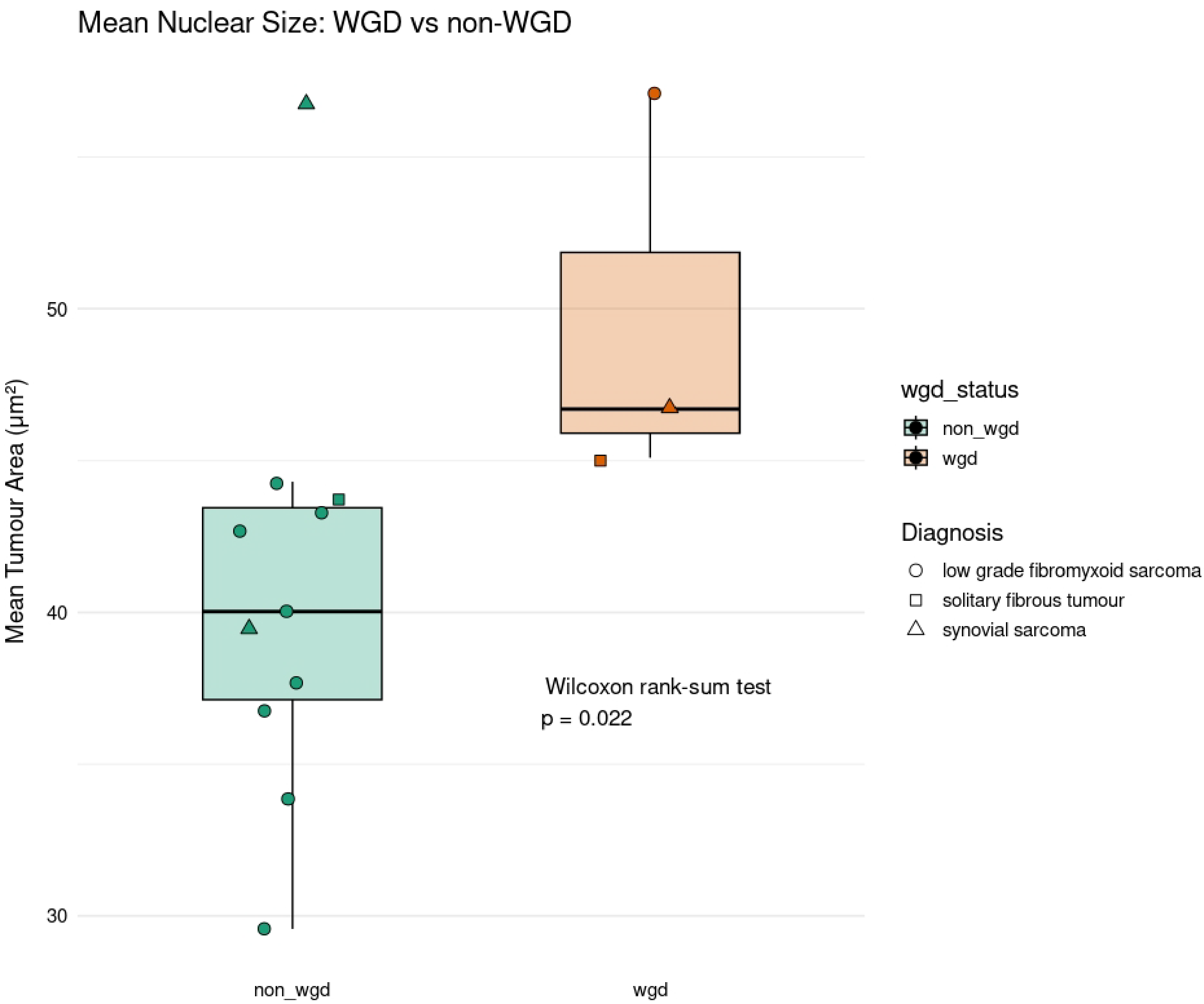

Supplementary Figure 16. Mean nuclear size of translocation-associated sarcomas grouped by whole-genome doubling status, related to Figure 4..

The figures shows the mean nuclear size of a subset of 14 translocation-associated sarcomas with relatively simple genomes. By CellVit analysis, the WGD tumours (identified through FC) have a greater mean nuclear size than all but one diploid sarcoma.

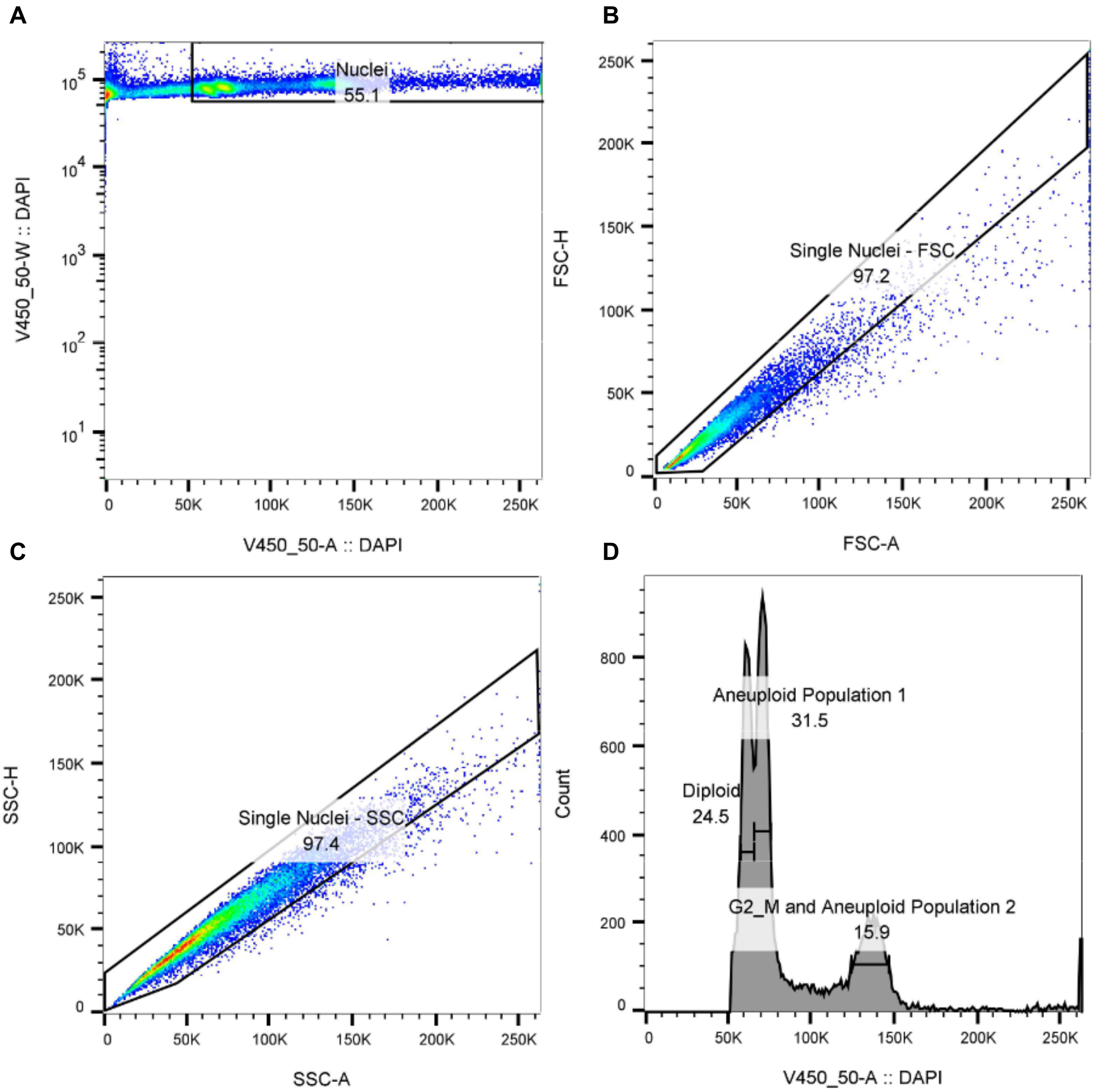

Supplementary Figure 17. Flow cytometry gating strategy for isolating nuclei and excluding debris and doublets, related to STAR Methods.
A) Using DAPI.width plotted against DAPI.area, the debris is excluded from the nuclei by identifying the first ‘hotspot’ which does not touch the y-axis. B+C) Doublets are excluded by creating hierarchical gates with FSC and SSC using the previously gated ‘nuclei’ population. Nuclei which fall off a linear relationship between area and height are considered as doublets. D) Ploidy histogram showing an aneuploid tumour sample. The first peak is the internal diploid control population. The second peak is the first aneuploid population. The third peak is the G2/M phase of the first aneuploid population. As the third peak has a population of greater than half of the aneuploid population, and a relatively small S-phase, it is possibly a mixture of G2/M nuclei, and a second aneuploid population.

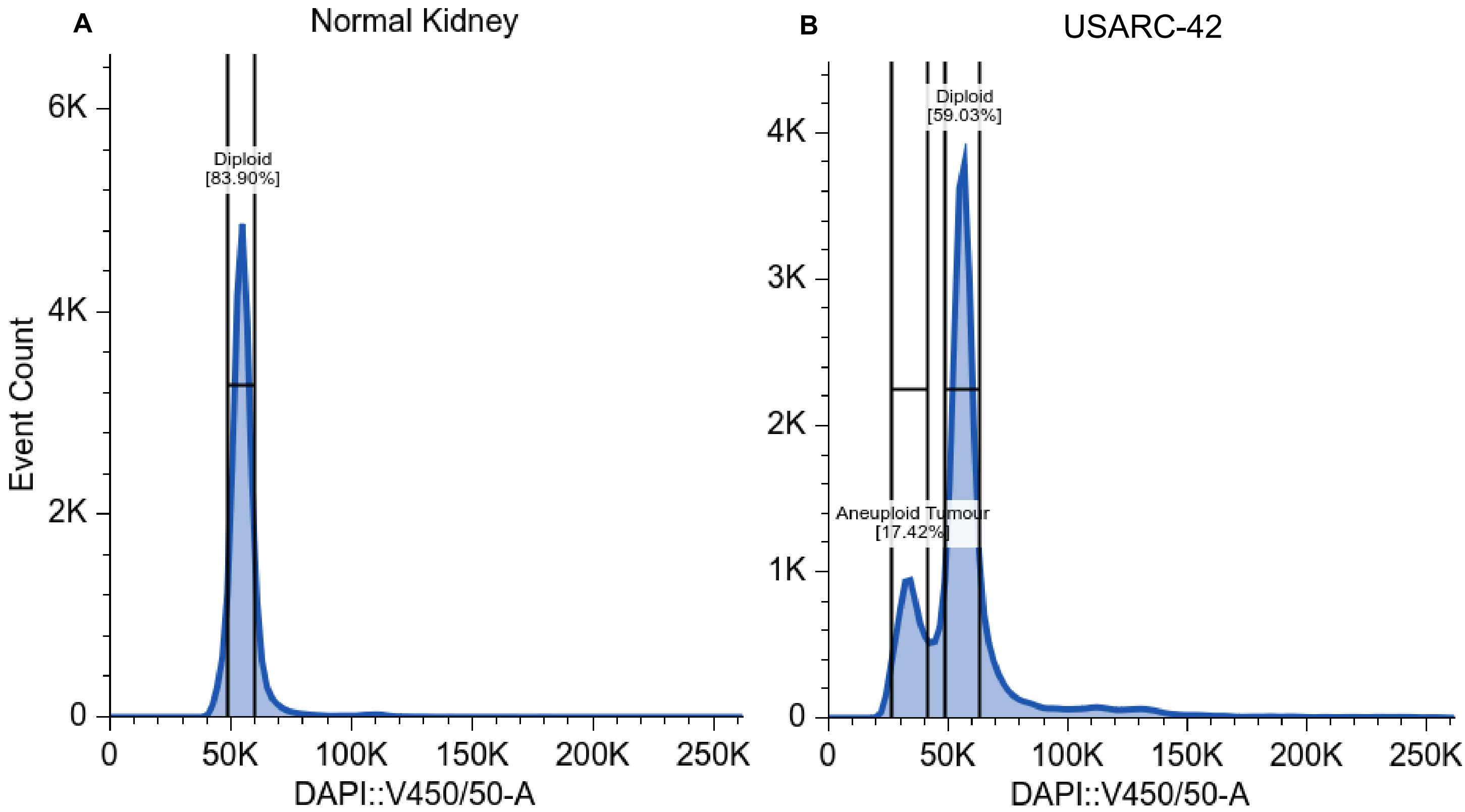

Supplementary Figure 18. Identification of a pseudo-haploidised tumour population in USARC-42 using an external diploid control, related to STAR methods.
Ploidy histogram of USARC-42 which demonstrates a pseudo-haploidised population of tumour nuclei at a ploidy of 1.2N. This haploid tumour exemplifies the importance of using an adequate and standardised external control. At first glance, the aneuploid population may be perceived as the diploid population in the tumour sample as it lies leftmost on the histogram. However, the second peak in the tumour sample lies at a MFI of 57,083AU which is very similar to the external diploid control sample (MFI 55,468AU) compared with the pseudo-haploidised peak (MFI 34,707AU).
